# DNA methylation analysis of phenotype specific stratified Indian population

**DOI:** 10.1186/s12967-015-0506-0

**Published:** 2015-05-08

**Authors:** Harish Rotti, Sandeep Mallya, Shama Prasada Kabekkodu, Sanjiban Chakrabarty, Sameer Bhale, Ramachandra Bharadwaj, Balakrishna K Bhat, Amrish P Dedge, Vikram Ram Dhumal, GG Gangadharan, Puthiya M Gopinath, Periyasamy Govindaraj, Kalpana S Joshi, Paturu Kondaiah, Sreekumaran Nair, SN Venugopalan Nair, Jayakrishna Nayak, BV Prasanna, Pooja Shintre, Mayura Sule, Kumarasamy Thangaraj, Bhushan Patwardhan, Marthanda Varma Sankaran Valiathan, Kapaettu Satyamoorthy

**Affiliations:** Division of Biotechnology, School of Life Sciences, Manipal University, Manipal, Karnataka 576104 India; Department of Rognidana/Shalyatantra, Shri Dharmasthala Manjunatheshwara College of Ayurveda, Udupi, Karnataka India; Department of Biotechnology, Sinhgad College of Engineering, Pune, Maharashtra India; Centre for Clinical Research, Foundation for Revitalization of Local Health Traditions, Bangalore, Karnataka India; CSIR-Centre for Cellular and Molecular Biology, Hyderabad, Andhra Pradesh India; Department of Molecular Reproduction, Development and Genetics, Indian Institute of Science, Bangalore, Karnataka India; Department of Statistics, Manipal University, Manipal, Karnataka India; Interdisciplinary School of Health Sciences, University of Pune, Pune, India

**Keywords:** DNA methylation, MeDIP, CpG Island, *Prakriti*, Ayurveda

## Abstract

**Background:**

DNA methylation and its perturbations are an established attribute to a wide spectrum of phenotypic variations and disease conditions. Indian traditional system practices personalized medicine through indigenous concept of distinctly descriptive physiological, psychological and anatomical features known as *prakriti*. Here we attempted to establish DNA methylation differences in these three *prakriti* phenotypes.

**Methods:**

Following structured and objective measurement of 3416 subjects, whole blood DNA of 147 healthy male individuals belonging to defined *prakriti* (*Vata*, *Pitta* and *Kapha*) between the age group of 20-30years were subjected to methylated DNA immunoprecipitation (MeDIP) and microarray analysis. After data analysis, *prakriti* specific signatures were validated through bisulfite DNA sequencing.

**Results:**

Differentially methylated regions in CpG islands and shores were significantly enriched in promoters/UTRs and gene body regions. Phenotypes characterized by higher metabolism (*Pitta prakriti*) in individuals showed distinct promoter (34) and gene body methylation (204), followed by *Vata prakriti* which correlates to motion showed DNA methylation in 52 promoters and 139 CpG islands and finally individuals with structural attributes (*Kapha prakriti*) with 23 and 19 promoters and CpG islands respectively. Bisulfite DNA sequencing of *prakriti* specific multiple CpG sites in promoters and 5′-UTR such as; *LHX1* (*Vata prakriti*), *SOX11* (*Pitta prakriti*) and *CDH22* (*Kapha prakriti*) were validated. *Kapha prakriti* specific *CDH22* 5′-UTR CpG methylation was also found to be associated with higher body mass index (BMI).

**Conclusion:**

Differential DNA methylation signatures in three distinct *prakriti* phenotypes demonstrate the epigenetic basis of Indian traditional human classification which may have relevance to personalized medicine.

**Electronic supplementary material:**

The online version of this article (doi:10.1186/s12967-015-0506-0) contains supplementary material, which is available to authorized users.

## Introduction

DNA methylation at cytosine bases of CpG dinucleotide is an epigenetic phenomenon widely used to regulate gene expression in humans [1]. CpG islands are often present in 5′-upstream region of genes and methylation of these is associated with condensed chromatin and repressed gene transcription [2,3]. As a heritable change, DNA methylation plays an important role in embryonic development, genomic imprinting and X-chromosome inactivation [4,5]. Alterations in DNA methylation is known to be affected by number of environmental factors to influence human diseases and is also associated with genetic events that leads to several types of cancer [6]. Growing evidences suggest that enzymatic machineries such as DNA methyltransferases (DNMTs) are responsible for creating and maintaining patterns of DNA methylation in response to environmental factors and during developmental programs [7,8].

The broad classification of phenotypic variations based on physiological, anatomical and psychological evaluation is being practiced in traditional Indian system of medicine Ayurveda [9,10]. Ayurveda suggests that principles of these evaluations are the basis of individuals life course and health events established at birth or *prakriti* which can be identified as *Vata*, *Pitta* and *Kapha* phenotypes with discrete characteristics [11,12]. The human classifications based on the body constitution as *Vata*, *Pitta* and *Kapha prakriti* in Ayurveda has formed the basis of disease management and for practicing traditional personalized medicine [13]. Several studies have attempted to associate single nucleotide polymorphisms (SNPs) to identify genetic basis of *prakriti* classification such as for HLA alleles [14], fast and slow metabolizing activities of defined *CYP2C19* SNPs [15], inflammatory and oxidative stress SNP markers in rheumatoid arthritis[16], SNPs for external environmental adaptation such as *EGLN1* [17], risk factors for coronary artery diseases [18] and variations in platelet aggregation among *prakriti* phenotypes [19]. Gene expression analyses performed among the three *prakriti* have also identified biochemical and hematological differences [20]. Previously we have showed differences in expression of cluster differentiation markers CD14, CD25 and CD56 among *prakriti* phenotypes by immunophenotyping [21].

Phenotypic variations observed across vast human diversity cannot be explained by genetic factors alone and may encompass several epigenetic phenomenon. In a genome wide study, Heyn et al. [22] have successfully reported the identification of DNA methylation markers in three human populations namely, Caucasian-Americans, African-Americans and Hans-Chinese Americans and have suggested to contribute to natural human variations. Therefore, DNA methylation may be involved in human developmental plasticity, adaptation and manifestation of distinct phenotypes. We studied the association of natural DNA methylation variations in the context of human classification based on principles of *prakriti* to understand phenotypically described sub-groups. We report that *prakriti* based classification of individuals provide genome-wide distinct DNA methylation signatures which may help in deciphering the basis for human variation description.

## Methods

### Study participants

The protocol was approved by institutional ethics committee and samples were screened after obtaining written informed consent, from all the participants. The Institute of Ayurveda and Integrative Medicine (I-AIM), Bangalore, received a written ethical approval from the IAIM- Institutional Ethical Committee (IEC), Shri Dharmasthala Manjunatheshwara College of Ayurveda (SDMCA), Udupi, obtained written ethical consent from Shri Dharmasthala Manjunatheshwara College of Ayurveda, Udupi, Institutional Ethical committee and Sinhgad College of Engineering, Pune, obtained the ethical consent from the Ethics Committee of Interdisciplinary School of Health Sciences, Pune University.

Screening and selection for dominant *prakriti* phenotyping was performed by double blinded method in three different cross-sectional cohorts using classical ayurvedic parameters [9,10] and a validated software called Ayusoft (Ayusoft, C-DAC. Pune). Qualitative subjective assessment of *prakriti* by ayurvedic physician and was arbitrated by the use of Ayusoft, thus providing a quantitative approach of *prakriti* determination. Detailed background, phenotypes of *prakriti* and methodology for the selection of subjects are described in our previous publication (Additional file 1) [23]. Briefly, 3416 healthy male volunteers of age group between 20–30 years were screened after receiving the institutional consent. Among them, 971 volunteers showed one of the three *prakriti* predominantly (≥ 60%), from whom whole blood genomic DNA was isolated by standard procedure.

### Global methylation analysis by reverse-phase HPLC

Reverse-phase high performance liquid chromatography (RP-HPLC) method was employed with minor changes for global methyl cytosine (mC) estimation as described previously [24,25]. In brief, 1.0 μg of genomic DNA was treated with 1U of DNaseI (New England Biolabs, USA), denatured at 100^°^C for 10 minutes and rapidly cooled in ice followed by the addition of 1.0 U of Nuclease P1 (Sigma-Aldrich, Canada) and 2.0 U of calf intestinal phosphate (New England Biolabs, USA). The 5-mC content was estimated in duplicate by injecting the sample to the RP-HPLC using C18 (Grace Vydac, Hesperia, CA, USA) columns. Isocratic delivery of mobile phase consisted of 50 mM potassium dihydrogen phosphate (pH 3.5) and methanol mixed in 9:1 ratio respectively, with flow rate of 1 mL/min. The percentage of 5-mC was calculated by using the formula [(5-mC peak area)/(C peak area /5-mC peak area)] /100 (where C is Cytosine and 5-mC is 5-methyl Cytosine)].

### Methylated DNA immunoprecipitation (MeDIP) Microarray

Briefly, 10 μg of whole blood genomic DNA was sonicated by using a 2 mm probe at amplitude 40 for 30 cycles with 15 seconds on and off, to obtain fragment sizes of 100–800 base pairs. About 3 μg of sheared DNA (INPUT) was immunoprecipitated overnight with anti-5-mathyl cytosine antibody (Diagenode, Belgium) along with positive and negative control DNA provided in the Diagenode kit. Enriched methylated fragments were purified by standard phenol-chloroform-ethanol precipitation [26]. The quality analysis of enriched immunoprecipitated (IP) methylated fragments was evaluated by real time PCR with positive, negative, *GAPDH* and *AX1* primers (Diagenode, Belgium) [27]. INPUT and IP were differentially labeled with cyanine3 and cyanine5 (Amersham Biosciences, USA) by indirect method using Bio-prime Array CGH kit (Invitrogen, USA) [28,29]. Equal concentrations of labeled INPUT and IP DNA were co-hybridized onto the Agilent 244K Human CpG Island, high density oligonucleotide array at 65°C for 40 hours with the continuous rotation at 18 rpm as per the Agilent MeDIP protocol version 1.1 (Agilent Technology, USA). The slide was washed using wash buffers (Agilent Technologies, Santa Clara, CA, USA), dried and scanned using G2505B DNA microarray scanner (Agilent Technology, USA) with Sure Scan High resolution technology (Additional file 1).

### Microarray Data Analysis

Total of 147 randomly selected male volunteers of dominant *prakriti* (*Kapha* = 52, *Pitta* = 48 and *Vata* = 47) were used for data analysis. Feature extraction software v10.1 was used to obtain background corrected, loess normalized, and logarithmic converted intensity values for green and red channels for each probe. Logarithmic difference of red to the green signal is the measure of methylation, generally represented as log ratio or (INPUT/IP) or “M-value” (methylation) for a given probe. Subsequent quality analysis was performed using R v2.15 with Bioconductor package limma. Quantile normalization was adopted for between array correction to remove batch effect and to equalize the overall signal intensities. Statistical significance within and between the groups was evaluated by Benjamini-Hochberg false discovery rate (FDR), as reported in the literature [30-32] FDR correction was implemented to control the spurious errors from multiple comparison tests and threshold of 0.05 and 0.2 was robust to call three *prakriti* specific differentially methylated regions. Quantile normalization and statistical test with FDR correction was performed using Gene Spring v12.6.

The differential methylated regions were identified at the probe level by both intra and inter-group analysis using relevant statistical measure. Intra-array probe level analysis was performed for the three *prakriti* using single sample t-statistics with Benjamini and Hochberg FDR correction to filter, most significant probes (p ≤ 0.05) with fold change of ≥1.5. Inter *prakriti* analysis was performed to find out the differentially methylated probes using analysis of variance with Tukey-post hoc test. Subsequently, differential methylated probes were identified with a fold change difference of ≥1.2 and 20% Benjamini and Hochberg FDR correction. The cross comparison of the intra-*prakriti* with inter-*prakriti* analysis was performed to find the *prakriti* specific differentially variable probes (mPSR). The differential methylated significant probes from the comparative analysis were hierarchically clustered using Pearson centered, Ward’s distance matrix. The identified mPSRs were analyzed across *prakriti* using Manhattan linkage and Ward’s distance matric. Intra-*prakriti* analysis by t-statistics with FDR correction and fold change analysis helped to find potential and, consistent methylated probes, whereas the ANOVA analysis identified differentially methylated probes. The identified mPSRs were analyzed with reference to CpG Islands and we designated differentially methylated if a) it contained at least one significant probe which fulfilled two criteria of probe level analysis and b) it should contain two or more methylated probes with fold change of ≥1.5 and p ≤ 0.05 in one of the three *prakriti* groups. For validation purpose, we have considered such differentially methylated CpG islands.

### Association of differential mPSRs with genomic regions

The probe sequences were annotated using Agilent e-array technology file and Galaxy genome browser into different genomic regions based on the distance from TSS of the gene. The probes were mapped to 5′-UTR and 3′-UTR regions using RefSeq gene coordinates. The enrichment of mPSRs at chromosomes, CpG Island, CpG shores and different genomic position was statistically analyzed by Chi-square test (p < 0.05). To understand the association of histone modification and CpG methylation, we used EpiExplorer and the statistical significance was estimated by the Fischer Exact test with FDR correction [33]. Functional relevance for *prakriti* specific methylated gene sets was constructed by Gene Ontology (GO) term, Kyoto Encyclopedia of Genes and Genomes (KEGG) pathway enrichment analysis by DAVID [34] and Funrich v2.1 (http://www.funrich.org/) tools with default parameters. The biologically enriched significant terms were deciphered by analyzing the *prakriti* specific methylated genes against the whole genome with hyper geometric test and p ≤ 0.05 without FDR correction was considered.

### Validation by bisulfite sequencing (BS)

Bisulfite conversion of whole blood DNA was performed using the EZ DNA Methylation-Gold kit (Zymo Research, Orange, CA, USA) according to manufacturer’s protocol. In brief, about 1.5 μg of total blood genomic DNA for each dominant *prakriti* was treated sodium bisulfite and bisulfite specific PCR was performed. The amplicons were purified and sequenced using BigDye terminator cycle sequencing kit (ABI, USA) [35]. The quality of the sequencing results and absolute methylation at CpG position were analyzed using ESME software [36]. Two way ANOVA with Bonferroni multiple correction was used to account for the significance (p < 0.05). For each selected *prakriti* specific differentially methylated region, 20 dominant *prakriti* samples were chosen for validation. The primer list along with chromosomal coordinates and PCR conditions are provided in the supplementary information (Additional file 2: Table S1).

## Results

### Analysis of global methylation variation

Global DNA methylation estimation (5-mC) was performed for 36 samples randomly selected (n = 12 for each *prakriti*), using reverse phase HPLC methodology. We observed a significantly higher level in global methyl cytosine content (p < 0.05) in *Vata* when compared with *Pitta* and *Kapha prakriti* (Additional file 3: Figure S1).

### Intra-*prakriti* methylation analysis

**Figure 1:**
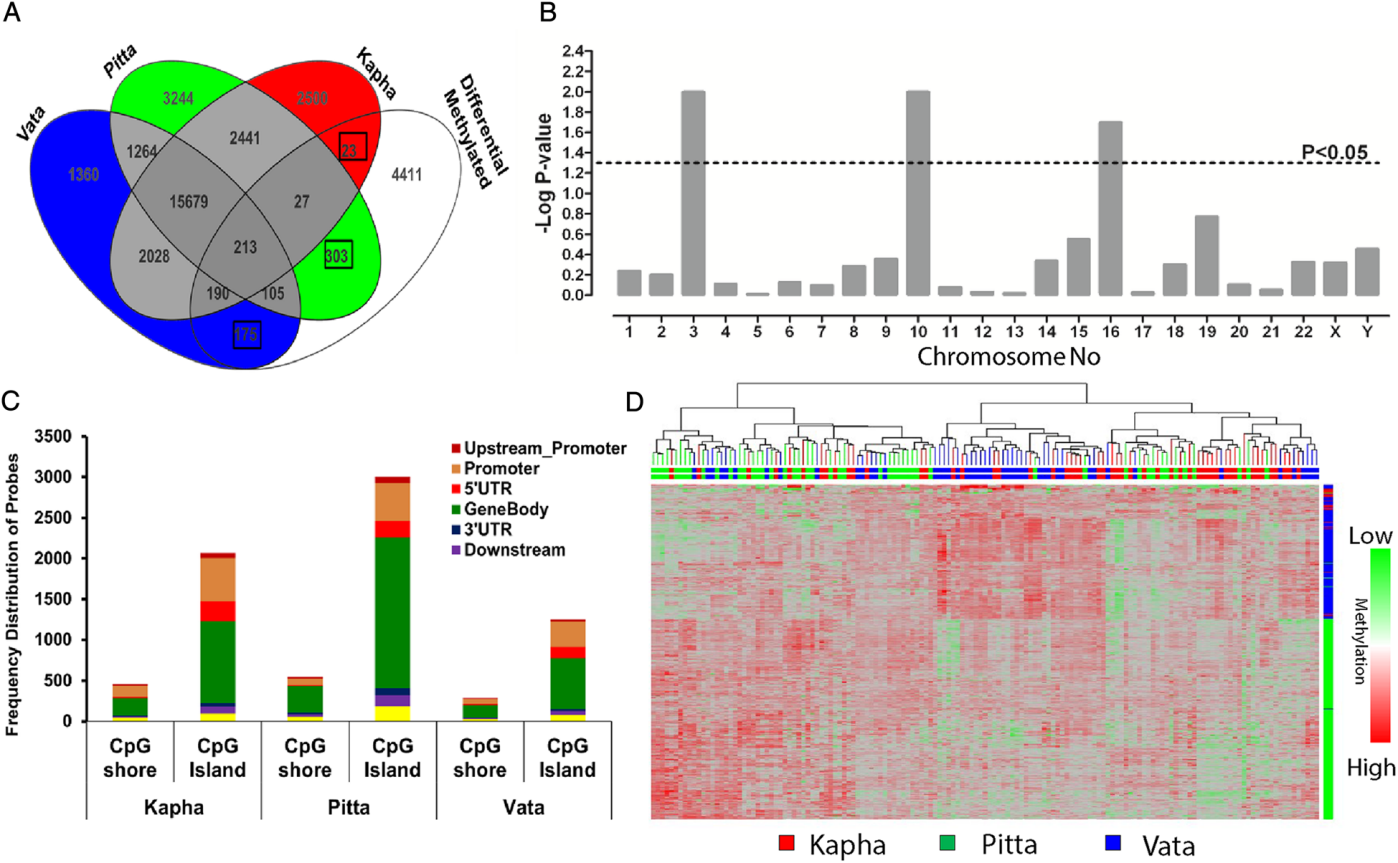
Comprehensive differential DNA methylation analysis in *prakriti*. **(A)** Venn diagram for the identification of *prakriti* specific methylated probes. The numbers indicated in the black boxes represent the identified 501 mPSRs in which 23, 303 and 175 probes were found to be more methylated in *Kapha*, *Pitta* and *Vata* respectively. **(B)** Distribution of uniquely significant methylated probes with respect to CpG islands and CpG shores. The probes are classified on the basis of its position from TSS, as upstream promoter (−10Kb to -1Kb), promoter (−1Kb to transcription start site), 5′UTR, gene body (within the genic region), 3′UTR, Downstream (Up to 10Kb from the end of gene coordinates) and Intergenic. **(C)** Genome wide distribution of 501 mPSRs. Bar height indicate the degree to which each chromosome contains an unexpectedly high number of differentially methylated probes. Bar height is the –log10 values of fisher exact p-value derived from no FDR correction. The height of bars higher than black dashed line was considered to be significant (p ≤ 0.05). **(D)** Hierarchical cluster analysis of 501mPSR. M-values scale represents the relative methylation. Column represents the samples and rows represented by 501mPSRs. The arrays and probes belonging to *Kapha*, *Pitta* and *Vata* are shown in red, green and blue respectively.

The intra-*prakriti* methylated probes were identified by providing a cutoff of fold change (FC) ≥1.5 with 5% FDR, based on the previous reports [30,37]. This analysis ensured the consistency of M-values across the *prakriti* groups and observed a total of 23,101, 23,276 and 21,014 significantly methylated probes in *Kapha*, *Pitta* and *Vata prakriti* respectively. Venn analysis showed a total of 2523, 3547 and 1535 methylated probes uniquely representing *Kapha*, *Pitta* and *Vata prakriti* respectively (Figure 1A). The distribution of probes based on their genomic location attributed to the nearest TSS site of a gene suggested a higher number of probes were associated with the gene body in *Pitta prakriti* as opposed to promoter associated probes in *Kapha prakriti* (Figure 1B). The Chi-square analysis on the distribution of methylated probes showed higher frequency at gene body, 5′UTR and 3′UTR, in case of *Pitta* (OR_p_ = 3.56, P < 0.001) as compared to other two *prakriti*s. A significant decrease in promoter methylated CpG probes in all three *prakriti* (OR_k_ = 0.88, OR_p_ = 0.79 and OR_v_ = 0.86, p < 0.001) was also observed (Additional file 4: Figure S2).

We identified, 735 (639 genes) *Pitta* specific, 561 (509 genes) *Kapha* specific and 305 (262 genes) *Vata* specific methylated CpG islands, represented by two or more methylated probes respectively. Evaluation of multiprobe methylated CpG Islands in the *prakriti* across the genome with respect to extended promoter (−10000 bp to 500 bp from TSS) and gene body (inside the gene coordinates) showed variations in *Kapha* and *Pitta prakriti* (Additional file 5: Figure S3). In order to integrate fold change and P-value significance cut-off for the inclusion of multiprobe methylated CpG Island, a preliminary validation of frequently methylated CpG Island at the promoter of an imprinted gene, neuronatin (*NNAT*), was performed in all three *prakriti* samples (n = 20). Results showed an average methylation level of about 40-60% in individual CpG sites across all the samples from three different *prakriti* (Additional file 6: Figure S4).

It is reported that 70% of CpG content of whole genome is represented in CpG Islands and about 50% of CpG islands encompasses promoter regulatory element to facilitate the expression variations among the individuals [38]. We show, the distribution of LogR values to be less near TSS compared to upstream and downstream regions, suggesting that the majority of the CpG islands near promoter regions were unmethylated. A comparative analysis of methylated promoter associated genes with the whole blood gene expression analysis data (http://biogps.org/downloads/) [39] revealed an inverse correlation confirming our results (Additional file 7: Figure S5).

### Differential methylation variation in *prakriti*

All 147 arrays irrespective of *prakriti* were subjected to quantile normalization. Analysis of variance (ANOVA) with Tukey post-hoc test at Benjamini Hochberg FDR of 20% correction was performed to enumerate differential methylated probes among *prakriti* samples. A total of 5447 differentially methylated probes were identified with fold change difference of ≥1.2 and FDR correction at P-value 0.2. These intra-prakrti probes were compared against significantly methylated probes of individual *prakriti* analysis. In all the, *Kapha* versus *Pitta*, *Pitta* versus *Vata* and *Vata* versus *Kapha* analysis, we observed 665 (*Kapha* vs *Pitta*), 755 (*Pitta* vs *Vata*) and 339 (*Vata* vs *Kapha*) probes to be differentially methylated. The supervised cluster analysis (Pearson’s distance metric and Ward’s linkage) of these differentially methylated probes (Additional file 8: Figure S6) indicated a distinct pattern of clusters of *prakriti* phenotypes. We determined a total of 501 *prakriti* specific differential methylated regions (mPSRs), represented by 303 probes overlapping with 269 CpG islands in *Pitta prakriti*, followed by *Vata prakriti* with 175 probes representing 139 CpG islands and finally *Kapha prakriti* with 23 probes and 19 CpG islands (Figure 1A) (Additional file 9: Table S2). The mPSRs were scattered across the genome and significantly (Chi-Square, p < 0.05) overrepresented at chromosome 3, 10 and 16 (Figure 1C). The supervised cluster analysis with Manhattan distance matrix and Ward’s linkage showed a prominent cluster of *Pitta* samples (Figure 1D) suggesting mPSRs could distinguish between *prakriti*. We did not find three prominent clusters of each *prakriti* due to a) selection of normal healthy individuals with > 60% of one *prakriti* and b) variation due to the influence of second dominant *prakriti* phenotype.

Further the mPSRs were significantly (p ≤ 0.05) enriched in the different genomic regions such as promoter and gene body associated with both CpG islands and shores (Additional file 10: Table S3). The crosstalk between DNA methylation and histone modifications illustrate influence of chromatin changes and has several implications including its role in normal variation, development, somatic cell reprogramming, and tumorigenesis [40]. To understand the possible variations, we compared the mPSRs with different regulatory regions using Epiexplorer tool [33]. We observed that the regions of H3K9ac, H3K27ac, H3K4me1, 2 and 3 and DNaseI hypersensitive regions were significantly (p≤0.05) augmented, underlining the effect of DNA methylation through intricate chromatin regulation. In addition to this, there was a significant (p ≤ 0.05) enrichment in the CTCF insulator sites (Additional file 11: Table S4).

### Association of mPSRs with Lymphocyte and BMI

It is established that differences in cell types may also possess non-overlapping DNA methylation signatures within population [41]. Hence, we systematically tested for such regions by comparing the mPSRs with the additional lymphocyte microarray data. Lymphocyte DNA from 11 healthy individuals was analyzed in a similar way as that of intra-*prakriti* analysis. The unmethylated significant probes of lymphocyte were compared with methylatd significant probes of whole blood data and also with uniquely represented methylated probes. However, we found very few probes showing such variations among the *prakriti*. Further, it was interesting to note that the methylation values of 501 identified mPSRs of whole blood data were positively significantly correlated with lymphocyte data (Spearman correlation R = 0.15 and p = 0.0004), indicating that the identified *prakriti* specific regions were distinct and were not influenced by variation in the blood cell types tested (Additional file 12: Figure S7).

**Figure 2:**
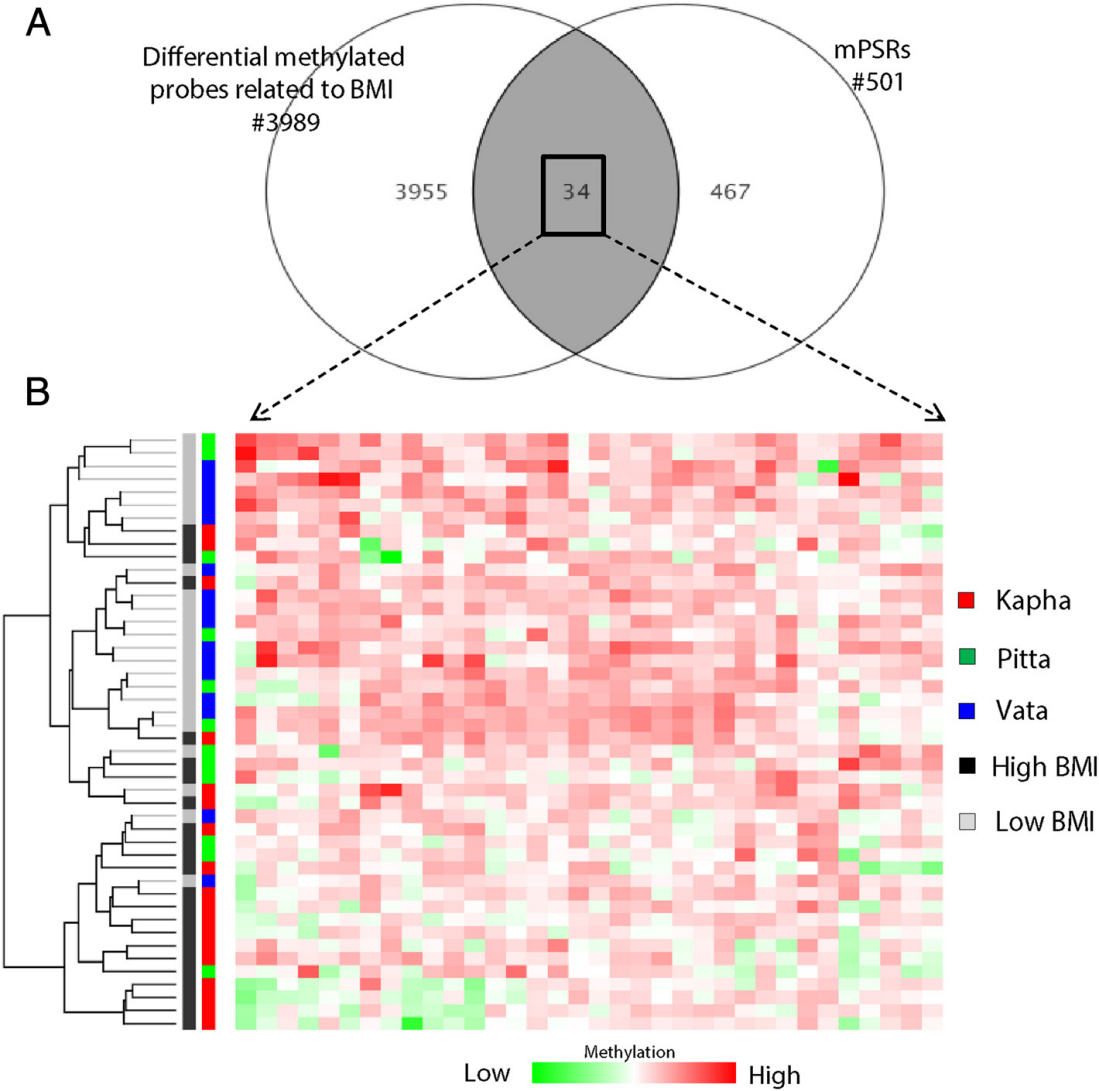
DNA methylome analysis of *prakriti* and BMI **(A)** Comparative analysis of differential methylated probes of BMI and mPSRs **(B)** Hierarchical cluster analysis for the significant differential methylated probes commonly existing among *prakriti* and BMI. M-values were scaled such that red indicates higher methylation in a row and green indicates lower methylation. Clustering was performed using Ward’s hierarchical clustering algorithm with Pearson correlation distance as the distance metric. Rows correspond to samples and columns to probe regions.

In a large population based study, we have previously shown an association between BMI and *prakriti* [23]. Hence, mPSRs were tested for their correlation to BMI by categorizing the hybridized samples into two groups: a) individuals with BMI less than 18 as low BMI (n = 23) and b) individuals with BMI greater than 25 as high BMI (n = 23). The arrays were quantile normalized and paired t-test with no correction method was used to identify differential methylated sequences. We found a total of 3989 probes with greater than ±1.2 fold change difference between high BMI and low BMI (p < 0.05) groups (Additional file 13; Figure S8). Among 501 mPSRs, 34 were common to differentially methylated BMI associated regions and were cluster distinctly with BMI as well as *prakriti* as well (Figure 2A and B). The genes corresponding to the 34 mPSRs were functionally associated with cellular adhesion or in the maintenance of cellular structural integrity and thus correlated with *Kapha prakriti*, characterized as energy of structure. Therefore cadherin22 precursor (*CDH22*) gene was selected for validation, to test the association of BMI group and *prakriti*.

### Functional relevance

The functional relevance of the identified *prakriti* specific methylated genes was analyzed using DAVID [34]. The mPSR associated genes were analyzed and top significant enriched gene ontology and pathway terms with a p ≤ 0.2 were represented in table S5 (Additional file 14: Table S5). We anticipated that the observed differential methylated genes at different genomic regions may influence gene expression leading to phenotypic variation among *prakriti*. Although promoter methylation is known to show inverse correlation of expression, recent reports have suggested the significance of the gene body methylation and its role in gene expression regulation [42]. The rationale of the present study was to test the hypothesis for the *prakriti* specific methylation variation in the whole blood DNA. Considering these, we chose all the mPSRs genes and attempted to associate to different attributes as energy of structure, metabolism and motion for *Kapha*, *Pitta* and *Vata* respectively [11,43,44].

Several methylated sequences of Vata prakriti were found to be represented in diverse biological processes such as cell communication, transcription, various signal transduction pathways and embryo morphogenesis. The associated genes are involved especially in neuronal development (*HOXB1*, *LMX1B*, *LHX1*, *LHX5*, *EN2*; p ≤ 0.05). In addition, we observed association of *NFIX* gene with low BMI which is one of the characteristic features of *Vata* prakriti. Pitta prakriti showed enrichment of mPSRs for metabolism related Gene Ontology terms and pathways. The biological process such as regulation of hormone secretion (*UNC13A*; p = 0.01), regulation of nucleic acid metabolism (*SKI, TP73, RUNX3, LMX1A, ZNF496, ZNF672, SOX11*; p = 0.03) and several others were significantly enriched. Apart from these, signal transduction pathways such as electron transport system, *NOTCH, SIP, AKT, BCR* signaling associated genes were also enriched in *Pitta* prakriti. Interestingly, we have observed regulation of *OCA2*, *MC1R* genes, which are associated with white to blonde or red hair colour in *Pitta* phenotype. Kapha mPSR associated genes were significantly enriched in cell growth/maintenance (*PRELP*, *COL2A1*, *ACTR10*, *CDH22*; p = 0.03), cytoskeleton anchoring activity (*PRELP*; p = 0.03) and cellular adhesion (*CDH22*, *CADM1*; p = 0.05) function.

### Microarray validation

Experimental validation on *prakriti* specific hypermethylated genes such as *LHX1*, *SOX11* and *CDH22* was performed. LHX1- a LIM homeobox 1, is a member of LIM domain which has cysteine-rich zinc-binding domain function as transcription regulator. The gene essentially involved in the development of retinal, urogenital system and localization and differentiation of tissues [45]. Recent report highlighted the disorganized circadian rhythm due to *LHX1* expression deficiency in suprachiasmatic nucleus [46]. Individuals with *Vata prakriti* have irregular sleeping and eating habits which is in accordance with the fragile circadian rhythm, and may be associated by LHX1 promoter methylation. The average methylation of the amplicon was found to be more in *Vata* in comparison to *Pitta* and *Kapha*. The post-hoc analysis with Bonferroni correction revealed a significant (p < 0.05) higher methylation at CpG site 47, 55 and 65 in *Vata prakriti* (Additional file 15: Figure S9).

*Pitta* methylated, *SOX11*, an intron less gene encodes a member of the SOX (SRY-related HMG box) family of transcription factors mainly involved in the regulation of embryonic development and in the determination of the cell fate, present in the chromosome 2p25.2. The protein encoded is a transcriptional regulator possessing diverse functions namely, visual [47], renal system development and in tumorigenesis [48]. Individuals with *Pitta prakriti* governs body metabolism, temperature regulation and development/proper function of eyes. Although, these provide only a causal relationship between the methylation and phenotype, a functional experimental study is essential. We observed that overall average methylation at all CpG sites was found to be higher in the *Pitta prakriti* when compared to other two (Additional file 16: Figure S10).

**Figure 3:**
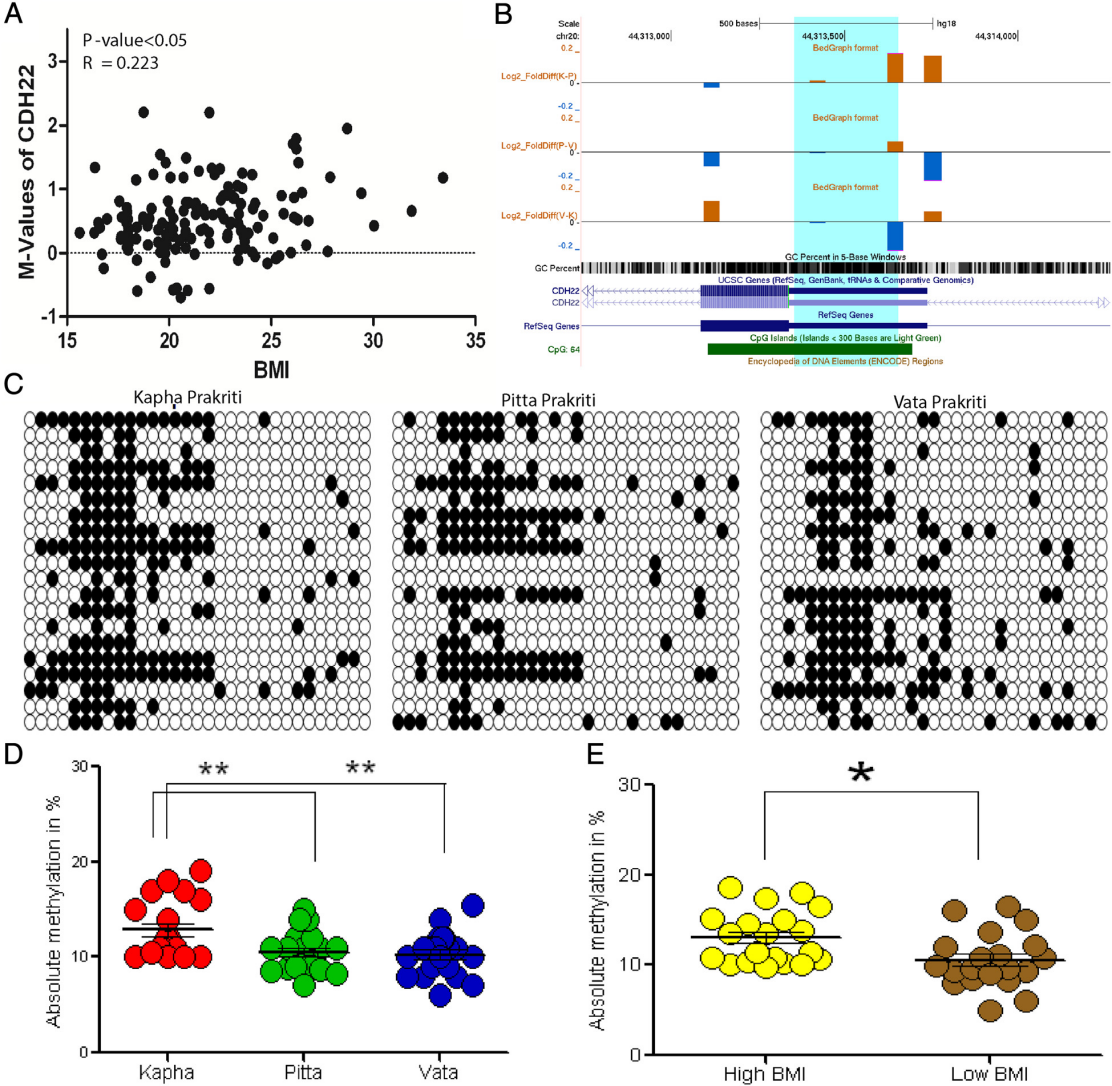
DNA methylation profiling at cadherin 22, precursor (*CDH22*) in *prakriti* and BMI. **(A)** Scatter plot illustrating positive, significant correlation (R = 0.22 and p ≤ 0.05) between the methylation values of *CDH22* and BMI. **(B)** Distribution of log fold change differences of *CDH22*, 5′UTR associated CpG island. The differences with positive values in yellow and negative with blue indicate level of methylation variation between *prakriti*. The height of the individual bars represents the extent of methylation differences. Shaded in blue is a probe region showing a significant difference at p ≤ 0.2 between *prakriti*. The offset scale for log2 fold differences was set to −0.2 to 0.2. **(C)** Bisulfite genome sequence analysis. Methylation cut off >15% was considered to call methylated sites and are represented in black circles. The circles in white were considered as unmethylated with respect to the cut-off chosen. Rows indicate the sample and columns represent individual CpG site. **(D)** and **(E)**, is the cumulative CpG site methylation variation in *prakriti* and BMI respectively. Each circle represents the overall methylation of amplicon for an individual. The methylation values in the groups is indicated with mean ± standard error and *refer to the significance (*for p ≤ 0.05 and **for p ≤ 0.01).

A *Kapha* specific and BMI associated cadherin family protein, cadherin 22 precursors (*CDH22*) was validated. The gene is mainly involved in the morphogenesis and tissue formation in neural and non-neural developmental process. The recent reports suggested that several known SNPs in the *CDH22* were associated with type II diabetes and higher BMI [49]. A differentially methylated probe’s M-value is significantly positively correlated to the BMI (Spearmann correlation R = 0.22 and p < 0.05) (Figure 3A). The *CDH22* amplicon was 301 bp in length, present at the 5′UTR of the gene, encompassing a total of 40 CpG sites out of which 33 CpG sites were analyzed in all the three *prakriti*. The fold change difference between *prakriti* underlined a higher methylation level for *Kapha prakriti* which was in concordant with the direct BS (Figure 3B and C). Overall, amplicon methylation was significantly (p < 0.05) high in *Kapha* and high BMI when compared to other *prakriti* and low BMI respectively. Apparently, absolute methylation of at individual CpG sites of individuals with *Kapha prakriti* and high BMI was found to be similar as opposed to individuals with *Vata prakriti* who have low BMI (Figure 3D and E).

## Discussion

DNA methylation plays central role in gene regulation and may influence variations in human phenotypes. Although DNA methylation is reported to be associated with several complex diseases including cancer [50,51], only limited studies have demonstrated its association with variations in the normal phenotypes. Ayurveda, an ancient Indian traditional medicine, classifies human population into three distinct *prakriti* phenotype as *Vata*, *Pitta* and *Kapha prakriti* [10,13]. The *prakriti* is a relative proportion of enumerated characteristics and may remain constant throughout the life span of an individual [52]. Ethnicity, familial characteristics, and habitat appear to influence phenotypic variability through their effect on *prakriti*. Similar factors also appear to contribute to inter-individual variability at the genetic or epigenetic levels [53].

However, precise molecular basis for the manifestation of such an effect remains to be understood. In the present study, we made an attempt to identify the methylation signatures associated with different *prakriti*s and the potential impact on determining distinct *prakriti* specific phenotypes. Our study showed that large proportions of DNA methylation patterns are common between *prakriti*s; observed differences in methylation signatures between *prakriti* suggests the presence of different mechanisms that may influence and sustain the expression of key regulator genes involved in the manifestation of distinct phenotypes.

From, intra-*prakriti* analysis, *Pitta prakriti* showed higher number of methylated CpG islands in gene body region, while promoters regions were more methylated in *Kapha prakriti*. CpG island associated within and outside the promoters as well as in the gene body can directly or indirectly affect the rate of transcription [3,54]. Methylation profile within individual CpG island is random and stochastic [55], therefore averaging the M-values or Z-score values for each CpG islands may not be sufficient to identify the differentially methylated CpG islands. Hence we used a simple methodology to identify differentially methylated signatures in the *prakriti* by considering methylated multiple probes associated CpG islands represented by a defined number of differentially methylated probes. Further, fold change cut-off of ≥1.5 for the methylated probe was used and as a proof of concept, a commonly methylated promoter region of imprinted *NNAT* gene was validated. To identify differential methylation between *prakriti*, ≥1.2 fold change cut-off, with FDR correction of 0.2 was provided as reported earlier [30]. We have also validated promoters and 5′UTR of three genes-*LHX1*, *SOX11* and *CDH22* which are found to be hypermethylated in *Vata*, *Pitta* and *Kapha prakriti* respectively. Validation of *LHX1*, *SOX11* and *CDH22* in a panel of *prakriti* samples showed concordance with the findings from microarray data.

Differences in DNA methylation patterns have been reported among various cell types in the human peripheral blood [41]. The comparative analysis of DNA methylation profile of lymphocyte and total blood DNA in specific to *prakriti* were minimal and do not influence much on the mPSRs. Thus, in this preliminary study, the 501 different mPSRs identified were sufficient to classify the individuals based on the *prakriti*; thereby providing a molecular evidence for the ancient concept of Ayurveda. The distribution of differentially methylated regions between the *prakriti* as well as within the *prakriti* were not uniform, suggesting that stochastic DNA methylation differences in *prakriti* may not be limited for the short-listed genes, but may influence multiple functional gene networks in multiple chromosomes. Recent reports have suggested that gene body DNA methylation may be involved in the regulation of gene expression either directly or indirectly [3]. The function of gene body DNA methylation remains unclear and recent studies have reported the potential for how gene body DNA methylation affects gene expression [56,57]. The gene-body-specific methylation has also been reported to be associated to replication timing and histone modification [58,59]. Our results have shown the enrichment of methylated CpG islands in gene body regions for *Pitta* and promoter regions for *Kapha prakriti*. This suggests that complex epigenomic variations within *prakriti* are regulated by genomic position of CpG methylated sequences.

Unlike *prakriti*, DNA methylation reprogramming events may also be determined during gestation, development and pregnancy. Several reports suggested that DNA methylation of cord, placental and whole blood were influenced by the season of conception, maternal diet, starvation, maternal stress by depression and anxiety, smoking and socio-economic conditions. Some of these have been implicated in stable phenotypes like low birth weight, height and obesity [60,61]. Therefore the identified mPSRs were correlated with BMI and showed the association of *Kapha prakriti* with high BMI and *Vata* with low BMI, which is in consistency with our earlier report [23]. Global 5-mC content between *Kapha* and *Pitta* there was no significant difference indicating that such similarity may be limited at the global level. Our study shows the importance of both global and gene specific methylation associated with *prakriti* phenotypes.

Gene ontology and pathway analysis for the mPSRs suggests the enrichment of gene related to characteristics of specific *prakriti*. The *Kapha* specific methylated genes were enriched in cellular adherence, protein localization and transport. The *Pitta* associated methylated specific genes were enriched in the various transcriptional DNA binding motifs, developmental morphogenesis and B-cell, T-cell receptor signaling pathway. *Vata* specific methylated genes were found to be associated with DNA sequence specific binding proteins involved in transcription regulation, nervous system development, metal ion binding motifs regulating cellular motion and cell-cell signaling. Thus the identified methylated genes may either directly or indirectly have an influence on the *prakriti* phenotypes. For example, *Kapha* specific mPSRs genes found enriched in cell adhesion molecules (*CDH22, CADM1, CDH11*) (p<0.05), there by regulate structure and integrity of the cells. Further the concordance of *CDH22* methylation validation was evident to find the association of *Prakriti* and BMI. The mechanisms and causes which sustain these changes in relation to *prakriti* determination needs further investigation and also demands for the case–control or retrospective large scale epigenome wide studies based on *prakriti*. However, we could able to demonstrate presence of the methylation signatures correlating to *prakriti* and its distinct phenotype specifically the BMI as an example. Thus, our results provide evidence for the identified genes for the specific *prakriti* attribute directly or indirectly to bring about defined phenotypes and suggest the impact of DNA methylation on specific *prakriti*.

## Conclusion

Therapy in Ayurveda is based on the premise of maintaining human body or any living cell as an open system, which can partake with the external environment. *Prakriti* may govern functions at cellular, tissue and at system levels maintaining equilibrium determined by genes which in turn are regulated by epigenetic cross talk especially through DNA methylation. These may provide basis to explore whether the DNA methylation programmed at the time of birth of an individual that will mimic the *prakriti* constitutions. This is suggested on the basis of genetic, biochemical, hematological or anatomical features already identified for the specific *prakriti*. Therefore, our study on the epigenetic basis of *prakriti* is a step towards deciphering diagnostic and prognostic utilities of traditional medicine by identifying genome-wide DNA methylation variants. In summary, we have identified DNA methylation signatures that distinguish the three major *prakriti*, which are responsible for imparting variations in humans. Taken together, DNA methylation is probably coupled to chromatin regulation as a contributor to different *prakriti* phenotypes such as *Vata*, *Pitta* and *Kapha*. The differentially methylated regions identified in our study needs further investigation as they may provide insight into epigenotype–phenotype correlation and that underlining molecular mechanisms of *prakriti* manifestations.

## Additional files

Additional file 1:
**Background information and supporting methods.**
Additional file 2: Table S1. Bisulfite Specific primers and PCR conditions. Additional file 3: Figure S1. Global methylation estimation (5-mC) using RP-HPLC method. Each dot represents the total methylation cytosine content of the individual *prakriti* sample. Within the *prakriti* groups mean ± standard error was shown in black lines. The aster sign indicates the P-value significance. ***p≤ 0.01, ***p*≤ 0.05. Additional file 4: Figure S2. Enrichment analysis of methylated *prakriti* associated regions in different genomic regions. Distribution of different genomic regions categorized based on the distance from TSS. Chi-square test was performed for the significance p ≤ 0.001 shown in three asters and p ≤ 0.01 shown in two aster signs respectively. Additional file 5: Figure S3. Distribution of CpG islands represented with two or more significantly methylated probes in three *prakriti*. (A) Venn diagrams showing uniquely methylated CpG island represented by two or more significantly methylated probes. (B) and (C) is the distribution of identified unique CpG islands in promoter ( −10kb to +500bp) and gene body (within genic coordinates as per RefSeq gene coordinates). The promoter specific methylated CpG islands are more represented in *Kapha prakriti* and gene body associated CpG islands in *Pitta prakriti*. Additional file 6: Figure S4. Bisulfite genome sequence analysis of *NNAT* promoter, a commonly methylated region. (A) Genomic location of the region selected for bisulfite specific PCR. (B) The individual CpG sites and its position in *NNAT* promoter region is shown. (C) Individual CpG sites absolute methylation in three *prakriti*. The color indicates the extent of methylation at individual CpG sites. (D) Overall methylation of the whole amplicon in individuals represented by mean ± standard error. (E) Methylation level at individual CpG sites represented by mean ± standard error. Additional file 7: Figure S5. Whole blood DNA methylation profile and its correlation with whole blood gene expression profile. (A) Variation in M-values with respect to distance from TSS. All significant methylated probes of within *prakriti* analysis were smoothened and represented in lines and the spread in the color to indicate the range of M-values in the *prakriti*. (B) Correlation analysis between M-value and whole blood expression. Whole blood expression values downloaded from BioGPS website and respective promoter associated genes M-values were converted to percentile values which is indicative of a fair negative correlation between DNA methylation and gene expression. Additional file 8: Figure S6. Heat map and clustering of *prakriti* differential methylation analysis (A), (B) and (C) are the supervised cluster analysis of significant (p≤0.2) identified specific to CpG loci, co-segregate as specific cluster in *Kapha* vs. *Pitta*, *Pitta* vs. *Vata* and *Vata* vs. *Kapha* respectively. Distinct co-segregation of differential methylated CpG sites in a *prakriti* specific manner was identified. Additional file 9: Table S2. List of 501mPSRs. Additional file 10: Table S3. Enrichment analysis of 501 differential methylated prakriti specific regions (mPSR) for genomic context and histone occupancy. Additional file 11: Table S4. Overall Enrichment for mPSRs in ENCODE regions. Additional file 12: Figure S7. Comparison analysis of methylation profiles of total blood (n=147) and lymphocyte (n=11) MeDIP microarray (A) Venn diagram representing differentially methylated probes (#3542) among total blood and lymphocyte DNA MeDIP microarray data. The significant methylated probes (p≤ 0.05 and Fold change of ≥1.5) of was assessed with the significant Unmethylated probes (p≤ 0.05 and Fold change of ≤−1.5) of lymphocyte. Nearly 16% of variation in methylation was observed between two different type of data. (B) Uniquely represented *prakriti* methylated probes form inter-*prakriti* analysis compared with differential methylated probes. The observed variable number of probes was very minimal. (C) Correlation analysis of M-values for the mPSRs among total blood and lymphocyte microarray data. The distribution and positive correlation with Spearman correlation R=0.15 was observed to be significant at p = 0.0004. Additional file 13: Figure S8. Association of BMI and *prakriti*. Hierarchical cluster analysis of significant differentially methylated probes in high BMI and low BMI. The rows represent the probes and columns represent the arrays with BMI and *prakriti* phenotype. Additional file 14: Table S5. Gene ontology and pathway analysis of prakriti methylated genes. Additional file 15: Figure S9. Overview of methylation status of *LHX1* in *prakriti*. A: Genomic position of amplicon, GC ratio and CpG density is shown at the top of the graphs. Green bar represents the CpG Island and arrow indicates the position of transcription start site. CpG density across a window size of 20bp is drawn. The red dotted box represents the amplicon for which bisulfite specific sequencing is performed. B: Average absolute methylation at individual CpG sites in three *prakriti*. The significant difference in methylation across CpG site is evaluated by two-way ANOVA and aster sign was represented for the significant (p*≤*0.05) CpG sites. C: Absolute methylation in individual CpG sites across three *prakriti*. A methylation cut off >10% was considered to call methylated sites and are represented in black circles. The circles in white were considered as unmethylated with respect to the cut-off given. The significant differentially methylated region is shown in red bordered circles. D: Quantitation of methylated CpG sites in the differentially methylated region with the same cut-off, highlighting the CpG sites with higher frequency of methylation in *Vata prakriti*. Cumulative methylation analysis for region of interest by averaging all CpG sites in the *prakriti* population showed higher level of methylation in *Vata* as compared to other *prakriti*. Additional file 16: Figure S10. Overview of methylation status of *SOX11* in *prakriti*. A: Genomic position of amplicon, GC ratio and CpG density is shown at the top of the graphs. Green bar represents the CpG Island and arrow indicates the position of transcription start site. CpG density across a window size of 20bp is drawn. The red dotted box represents the amplicon for which bisulfite specific sequencing was performed. B: Average absolute methylation at individual CpG sites in three *prakriti*. The significant difference in methylation across CpG site was evaluated by two-way ANOVA and aster sign was represented for the significant (p < 0.05) CpG sites. C: Absolute methylation in individual CpG sites across three *prakriti*. A methylation cut off >15% was considered to call methylated sites and are represented in black circles. The circles in white were considered as unmethylated with respect to the cut-off given. The significant differentially methylated region is shown in red bordered circles. D: Quantitation of methylated CpG sites in the differentially methylated region with the same cut-off, highlighting the CpG sites with higher frequency of methylation in *Vata prakriti*. Cumulative methylation analysis for region of interest by averaging all CpG sites in the *prakriti* population showed higher level of methylation in *Pitta* as compared to other *prakriti*.

## Abbreviations

BMI: Body Mass Index
ANOVA: Analysis of Variance
mPSRs: methylated *Prakriti* specific regions
TSS: Transcription Start Site
5′UTR: 5′Un-translated Region
GO: Gene Ontology
SNPs: Single Nucleotide Polymorphisms
5-mC: 5′-methyl cytosine
FDR: False Discovery Rate
KEGG: Kyoto Encyclopedia of Genes and Genomes
DAVID: Database for Annotation Visualization and Integrated Discovery
ESME: Epigenetic Sequencing methylation analysis

## References

[1] Suzuki MM, Bird A (2008). DNA methylation landscapes: provocative insights from epigenomics. Nat Rev Genet.

[2] Cedar H, Bergman Y (2012). Programming of DNA methylation patterns. Annu Rev Biochem.

[3] Jones PA (2012). Functions of DNA methylation: islands, start sites, gene bodies and beyond. Nat Rev Genet.

[4] Bjornsson HT, Daniele Fallin M, Feinberg AP (2004). An integrated epigenetic and genetic approach to common human disease. Trends Genet.

[5] Costello JF, Plass C (2001). Methylation matters. J Med Genet.

[6] You JS, Jones PA (2012). Cancer genetics and epigenetics: two sides of the same coin?. Cancer Cell.

[7] Pembrey M, Saffery R, Bygren LO, Carstensen J, Edvinsson S, Faresjö T, et al. Human transgenerational responses to early-life experience: potential impact on development, health and biomedical research. J Med Genet. 2014:jmedgenet-2014-102577. 10.1136/jmedgenet-2014-102577 PMC4157403 25062846

[8] Goll MG, Bestor TH (2005). Eukaryotic cytosine methyltransferases. Annu Rev Biochem.

[9] Caldecott T (2006). Ayurveda: The divine science of life.

[10] Valiathan M (2003). The Legacy of Caraka.

[11] Hankey A (2005). A test of the systems analysis underlying the scientific theory of Ayurveda’s Tridosha. J Altern Complement Med.

[12] Valiathan M (2009). Legacy of Vāgbhaμa.

[13] Jayasundar R (2010). Ayurveda: a distinctive approach to health and disease. Curr Sci.

[14] Bhushan P, Kalpana J, Arvind C (2005). Classification of human population based on HLA gene polymorphism and the concept of Prakriti in Ayurveda. J Altern Complement Med.

[15] Ghodke Y, Joshi K, Patwardhan B (2011). Traditional Medicine to Modern Pharmacogenomics: Ayurveda Prakriti Type and CYP2C19 Gene Polymorphism Associated with the Metabolic Variability. Evid Based Complement Alternat Med.

[16] Juyal RC, Negi S, Wakhode P, Bhat S, Bhat B, Thelma B (2012). Potential of ayurgenomics approach in complex trait research: Leads from a pilot study on rheumatoid arthritis. PLoS One.

[17] Aggarwal S, Negi S, Jha P, Singh PK, Stobdan T, Pasha MQ (2010). EGLN1 involvement in high-altitude adaptation revealed through genetic analysis of extreme constitution types defined in Ayurveda. Proc Natl Acad Sci USA.

[18] Mahalle NP, Kulkarni MV, Pendse NM, Naik SS (2012). Association of constitutional type of Ayurveda with cardiovascular risk factors, inflammatory markers and insulin resistance. J Ayurveda Integr Med.

[19] Bhalerao S, Deshpande T, Thatte U (2012). Prakriti (Ayurvedic concept of constitution) and variations in platelet aggregation. BMC Complement Altern Med.

[20] Prasher B, Negi S, Aggarwal S, Mandal AK, Sethi TP, Deshmukh SR (2008). Whole genome expression and biochemical correlates of extreme constitutional types defined in Ayurveda. J Transl Med.

[21] Rotti H, Guruprasad K, Nayak J, Kabekkodu S, Kukreja H, Mallya S (2014). Immunophenotyping of normal individuals classified on the basis of human dosha prakriti. J Ayurveda Integr Med.

[22] Heyn H, Moran S, Hernando-Herraez I, Sayols S, Gomez A, Sandoval J (2013). DNA methylation contributes to natural human variation. Genome Res.

[23] Rotti H, Raval R, Anchan S, Bellampalli R, Bhale S, Bharadwaj R (2014). Determinants of prakriti, the human constitution types of Indian traditional medicine and its correlation with contemporary science. J Ayurveda Integr Med.

[24] Kumar A, Rai PS, Upadhya R, Shama Prasada K, Satish Rao B, Satyamoorthy K (2011). γ-radiation induces cellular sensitivity and aberrant methylation in human tumor cell lines. Int J Radiat Biol.

[25] Magaña AA, Wrobel K, Caudillo YA, Zaina S, Lund G, Wrobel K (2008). High-performance liquid chromatography determination of 5-methyl-2′-deoxycytidine, 2′-deoxycytidine, and other deoxynucleosides and nucleosides in DNA digests. Anal Biochem.

[26] Weber M, Davies JJ, Wittig D, Oakeley EJ, Haase M, Lam WL (2005). Chromosome-wide and promoter-specific analyses identify sites of differential DNA methylation in normal and transformed human cells. Nat Genet.

[27] Pälmke N, Santacruz D, Walter J (2011). Comprehensive analysis of DNA-methylation in mammalian tissues using MeDIP-chip. Methods.

[28] Jia J, Pekowska A, Jaeger S, Benoukraf T, Ferrier P, Spicuglia S (2010). Assessing the efficiency and significance of Methylated DNA Immunoprecipitation (MeDIP) assays in using in vitro methylated genomic DNA. BMC Res Notes.

[29] Yan PS, Efferth T, Chen H-L, Lin J, Rödel F, Fuzesi L (2002). Use of CpG island microarrays to identify colorectal tumors with a high degree of concurrent methylation. Methods.

[30] Mah WC, Thurnherr T, Chow PK, Chung AY, Ooi LL, Toh HC (2014). Methylation profiles reveal distinct subgroup of hepatocellular carcinoma patients with poor prognosis. PLoS One.

[31] Lynn EG, McLeod CJ, Gordon JP, Bao J, Sack MN (2008). SIRT2 is a negative regulator of anoxia-reoxygenation tolerance via regulation of 14-3-3 zeta and BAD in H9c2 cells. FEBS Lett.

[32] Maratou K, Wallace VC, Hasnie FS, Okuse K, Hosseini R, Jina N (2009). Comparison of dorsal root ganglion gene expression in rat models of traumatic and HIV-associated neuropathic pain. Eur J Pain..

[33] Halachev K, Bast H, Albrecht F, Lengauer T, Bock C (2012). EpiExplorer: live exploration and global analysis of large epigenomic datasets. Genome Biol.

[34] Huang DW, Sherman BT, Lempicki RA (2008). Systematic and integrative analysis of large gene lists using DAVID bioinformatics resources. Nat Protoc.

[35] Engemann S, El-Maarri O, Hajkova P, Oswald J, Walter J, Ward A (2002). Bisulfite-based methylation analysis of imprinted genes. Genomic Imprinting.

[36] Lewin J, Schmitt AO, Adorján P, Hildmann T, Piepenbrock C (2004). Quantitative DNA methylation analysis based on four-dye trace data from direct sequencing of PCR amplificates. Bioinformatics.

[37] Rajendram R, Ferreira JC, Grafodatskaya D, Choufani S, Chiang T, Pu S (2011). Assessment of methylation level prediction accuracy in methyl-DNA immunoprecipitation and sodium bisulfite based microarray platforms. Epigenetics.

[38] Takai D, Jones PA (2002). Comprehensive analysis of CpG islands in human chromosomes 21 and 22. Proc Natl Acad Sci USA.

[39] Su AI, Wiltshire T, Batalov S, Lapp H, Ching KA, Block D (2004). A gene atlas of the mouse and human protein-encoding transcriptomes. Proc Natl Acad Sci USA.

[40] Vaissière T, Sawan C, Herceg Z (2008). Epigenetic interplay between histone modifications and DNA methylation in gene silencing. Mutat Res.

[41] Lam LL, Emberly E, Fraser HB, Neumann SM, Chen E, Miller GE (2012). Factors underlying variable DNA methylation in a human community cohort. Proc Natl Acad Sci USA.

[42] Huh I, Zeng J, Park T, Yi S (2013). DNA methylation and transcriptional noise. Epigenetics Chromatin.

[43] Hankey A (2001). Ayurvedic physiology and etiology: Ayurvedo Amritanaam. The doshas and their functioning in terms of contemporary biology and physical chemistry. J Altern Complement Med.

[44] Mukerji M, Prasher B (2011). Ayurgenomics: A new approach in personalized and preventive medicine. Sci Cult.

[45] Kobayashi A, Shawlot W, Kania A, Behringer RR (2004). Requirement of Lim1 for female reproductive tract development. Development.

[46] Bedont JL, LeGates TA, Slat EA, Byerly MS, Wang H, Hu J (2014). Lhx1 controls terminal differentiation and circadian function of the suprachiasmatic nucleus. Cell reports.

[47] Cizelsky W, Hempel A, Metzig M, Tao S, Hollemann T, Kühl M (2013). sox4 and sox11 function during Xenopus laevis Eye development. PLoS One.

[48] Hide T, Takezaki T, Nakatani Y, Nakamura H, Kuratsu J-I, Kondo T (2009). Sox11 prevents tumorigenesis of glioma-initiating cells by inducing neuronal differentiation. Cancer Res.

[49] Lewis JP, Palmer ND, Ellington JB, Divers J, Ng MC, Lu L (2010). Analysis of candidate genes on chromosome 20q12-13.1 reveals evidence for BMI mediated association of PREX1 with type 2 diabetes in European Americans. Genomics.

[50] Feinberg AP (2007). Phenotypic plasticity and the epigenetics of human disease. Nature.

[51] Feinberg AP (2010). Epigenomics reveals a functional genome anatomy and a new approach to common disease. Nat Biotechnol.

[52] Tripathi N (2011). Concept of formation of “Prakriti” in ayurveda. Ind J Res.

[53] Bock C, Walter J, Paulsen M, Lengauer T (2008). Inter-individual variation of DNA methylation and its implications for large-scale epigenome mapping. Nucleic Acids Res.

[54] Maunakea AK, Nagarajan RP, Bilenky M, Ballinger TJ, D’Souza C, Fouse SD (2010). Conserved role of intragenic DNA methylation in regulating alternative promoters. Nature.

[55] Zhang Y, Rohde C, Tierling S, Jurkowski TP, Bock C, Santacruz D (2009). DNA methylation analysis of chromosome 21 gene promoters at single base pair and single allele resolution. PLoS Genet.

[56] Kulis M, Heath S, Bibikova M, Queirós AC, Navarro A, Clot G (2012). Epigenomic analysis detects widespread gene-body DNA hypomethylation in chronic lymphocytic leukemia. Nat Genet.

[57] Lister R, Pelizzola M, Dowen RH, Hawkins RD, Hon G, Tonti-Filippini J (2009). Human DNA methylomes at base resolution show widespread epigenomic differences. Nature.

[58] Aran D, Toperoff G, Rosenberg M, Hellman A (2011). Replication timing-related and gene body-specific methylation of active human genes. Hum Mol Genet.

[59] Hahn MA, Wu X, Li AX, Hahn T, Pfeifer GP (2011). Relationship between gene body DNA methylation and intragenic H3K9me3 and H3K36me3 chromatin marks. PLoS One.

[60] Reynolds RM, Jacobsen GH, Drake AJ (2013). What is the evidence in humans that DNA methylation changes link events in utero and later life disease?. Clin Endocrinol (Oxf).

[61] Waterland RA, Kellermayer R, Laritsky E, Rayco-Solon P, Harris RA, Travisano M (2010). Season of conception in rural gambia affects DNA methylation at putative human metastable epialleles. PLoS Genet.

