# Supplementary material for: DNA methylation analysis of phenotype specific stratified Indian population
Source: J Transl Med. 2015 May 8;13:151. doi: 10.1186/s12967-015-0506-0 (PMC4438459; doi:10.1186/s12967-015-0506-0)
Supplement: Additional file 1: — Background information and supporting methods. [file 12967_2015_506_MOESM1_ESM.pdf]

Additional File 1

Background and Methods

Background for *Prakriti*

Genomic catalogue of human population based on the geographical distribution, ethnicity, religion and caste are limited. The Ayurveda, an Indian traditional medicine defined as science or knowledge of life, offers personalized medicine by classifying humans based on the body constitution or *prakriti*. Ayurveda uses them as a cornerstone guiding lifestyle, dietary and treatment modality [1, 2]. Various DNA methylation studies have shown the association with population specific human variation exhibited due to differences in maternal diet [3] and socio-economic condition [4]. Thus the determinants of *prakriti* may have similar relationships and investigations on DNA methylation signatures in a *prakriti* classified population may delineate the scientific molecular basis for *prakriti* classification and contribute significantly to the translational research in future.

Ayurvedic human *dosha prakriti* classifies the human population into seven groups which are manifested by combination of three different body types. The dominant *prakriti* characteristics has been listed in the below table. In the present study ≥60% of *prakriti* was considered as dominant and selected for sampling. Advances in technology have provided extensive information on genes and gene expression, resulting in precise mapping of genes associated with various biological functions. Such advancement in technology has now established better way of understanding different components of human biology such as classical inheritance patterns, and more recently, genetic basis of numerous diseases and even physiological pathways. Thus, we have made an attempt to understand the molecular basis of human classification of Ayurveda by using high throughput technology which may contribute by providing scientific evidence to the ancient knowledge of science.

Methods

Screening and selection of dominant *prakriti* subjects

The screening and sampling for dominant p*rakriti* was carried-out from three centers namely Institute of Ayurveda and Integrative Medicine (I-AIM), Bangalore (Centre1), Sinhgad College of Engineering (SCE), Pune (Centre 2) and Shri Dharmasthala Manjunatheshwara College of Ayurveda (SDMCA), Udupi (Centre 3). The protocol was approved by Institutional Ethics committees and samples were screened after obtaining written informed consent, from all the participants. A total of 3416 healthy male individuals of age group between 20-30 years were screened for their *prakriti*, after obtaining their informed written consent. The detailed *prakriti* assessment with inclusion, exclusion criteria was recently published [5]. Briefly, *prakriti* assessment of each subject was performed in three stages. In the first stage**,** senior ayurvedic physician assessed the *prakriti* of the subject based on his clinical skill and experience, applying classical ayurvedic parameters of *prakriti* determination [2, 6]. In the second stage**,** the same subjects were assessed through Ayusoft *prakriti* assessment by a ayurvedic physician, which contain a comprehensive questionnaire; formulated based on information from original Ayurvedic literature and used for qualitative and quantitative determination of *prakriti*. In the third stage another ayurvedic physician who is unaware of the outcome of both senior physician and ayusoft, compares the *prakriti* analysis and those subjects who have same *prakriti* determined by both, with greater than 60% single *prakriti* dominance were considered in this study. Subjects with equal to or greater than 60% of dominant *prakriti* were selected and blood was drawn according to standard procedure. The blood sample blinding sheet was provided with percentage values of *prakriti*, and sample code with date of collection. All subject's information sheet with proper code and also Ayusoft *prakriti* percentage from each center was collected and Body Mass Index (BMI) were tabulated in Microsoft Office Excel 2007. The concordance of 80% was observed in the determination of *prakriti*. Additionally, *Kappa* statistics revealed a substantial agreement with the value of 0.77 ($p < 0.001$) between Ayurvedic physician and Ayusoft. The data suggests fair agreement between the two methods. A total of 1311 subjects showed a predominant *prakriti* of greater than or equal to 60%, out of which 971 subjects were selected and whole blood genomic DNA was isolated by standard procedure.

Methylated DNA immunoprecipitation

Rrandomly chosen, unrelated dominant *prakriti* DNA samples were subjected to genome wide methylation analysis using methylated DNA immunoprecipitation (MeDIP) microarray analysis, as published previously [7]. Briefly 10μg of whole blood genomic DNA was sonicated by 2 mm probe at 40 amplitude for 30 cycles with 15 seconds on and off, to obtain fragment size of 100-800 base pairs. About 3 μg of sheared DNA (INPUT) was allowed to immunoprecipitated by anti-5-methyl cytosine antibody (Diagenode, Belgium) for overnight along with positive and negative control DNA provided in the Diagenode kit. Enriched methylated fragments were purified by standard phenol-chloroform-ethanol precipitation [8]. The immunoprecipitated (IP) enriched methylated fragments quality analysis was performed systematically and evaluated by real time PCR with positive, negative, *GAPDH* and *AX1* primers (Diagenode MeDIP Kit). The samples with % of INPUT/IP for positive and *AX1* >10, whereas for negative and GAPDH it is <1 was considered for the labeling process [9] and Diagenode, Belgium).

INPUT and IP were separately labeled with cyanine3 and cyanine5 (Amersham Biosciences, USA) by indirect method using Bio-prime Array CGH kit (Invitrogen, USA). Briefly, about 250ng INPUT and IP DNA were mixed separately with 1.25x random primers, which was denatured at 95^0C for 5 minutes. Immediately the INPUT and IP sample were placed on ice, to which 5μl of 10XdNTP mixture (2 mM each dATP, dCTP, and dGTP, and 0.35 mM dTTP), 1.8μl of 10 mM aminoallyl-dUTP (Ambion, Invitrogen), and 40 U of high-concentration Klenow$^{3'\rightarrow 5' exo-}$(New England Biolabs, USA) were added and reactions were kept at 37^0C for overnight and purification was performed using Qiagen PCR purification columns. Equal concentration of amino-ally-dUTP labeled INPUT and IP DNA was coupled with cyanine3 and cyanine5 (Amersham, GE Healthcare, USA) respectively. The optimum labelling of INPUT and IP was carried by maintaining base to dye ratio (40-80), amount of labeled targets (>2.5μg) and amount of total dye incorporated (>250 pico mole) ensured the quality of prepared targets [10, 11].

Equal concentration of labeled INPUT and IP DNA was co-hybridized onto the Agilent 244k Human CpG Island, high density oligonucleotide array as per the Agilent MeDIP protocol version 1.1 (Agilent Technology, USA) at 65^0C for 40 hours with the continuous rotation at 18 rpm. The slide was washed using wash buffers (Agilent Technologies, Santa Clara, CA, USA),

immediately dried and scanned using G2505B DNA microarray scanner (Agilent Technologies, USA) with Sure Scan High resolution technology. Feature extraction was performed by using Feature extraction software v10.1 to obtain background corrected, loess normalized, and logarithmic converted intensity values for green and red channels. Logarithmic difference of red signal to the green signal termed as log ratio or (INPUT/IP) or "M-value" (methylation) for a given probe was further selected for genome wide methylation analysis.

Microarray Analysis

The differential methylated regions were identified at the probe level by both intra and inter-group analysis using relevant statistical measure. A total of 147 randomly selected male samples of dominant *prakriti* (*Kapha*=52, *Pitta*=48 and *Vata*=47) were hybridized. Within the *prakriti*, all samples were quantile normalized. Unlike differential methylation hybridization, by employing MeDIP it is possible to identify methylated regions qualitatively based on the M-values. Therefore probe level analysis was performed within *prakriti* using single sample t-statistics with Benjamini and Hochberg false discovery rate correction at $p \leq 0.05$ to filter, most significant probes with fold change of ≥1.5. The approach of use of FDR with 5% error rate and fold change of cut-off of ≥1.5 ensured the consistency of methylation in each *prakriti* across samples. This approach has been widely used in various published literature [4, 12] and is also based on our preliminary results microarray validation by bisulfite genomic sequencing (Figure S4, Additional File 6). Venn diagrams were used to find the *prakriti* specific significant methylated probes. Further the probes coordinates were mapped to whole genome CpG Island using galaxy genome browser. Next, significant multiprobe methylated associated CpG islands were analysed to consider potential methylated CpG islands in *prakriti*.

Inter *Prakriti* analysis was performed to find out the differentially methylated probes using analysis of variance with Tukey-post hoc test, 20% Benjamini and Hochberg FDR correction and fold change difference of ±1.2. Further, the differential methylated probes of *prakriti* were compared with the significant methylated probes of intra-*prakriti* analysis to find the *prakriti* specific methylation varying regions (mPSR). The differential methylated significant probes from the comparative analysis were hierarchically clustered using Pearson centered, ward's distance matrix and showed a distinct clusters enriched with specific *prakriti*. The identified mPSRs were analyzed across *prakriti* using Manhattan linkage and ward's distance matric.

Benjamini-Hochberg false discovery rate correction (FDR) correction has been implemented to control the spurious errors from multiple comparison tests and threshold of 0.05 and 0.2 are robust to call *Prakriti* differentially methylated regions. Intra-*prakriti* analysis, by t-statistics with FDR correction and fold change analysis helped to find potential, consistent methylated probes, whereas the ANOVA analysis identified differential methylated probes. The identified mPSRs were analyzed in reference to CpG Islands and we designated a CpG Islands is differentially methylated if it possess at least one significant probe which fulfill both criteria of probe level analysis and secondly it should contain two or more methylated probes with fold change of $\geq$1.5 and P-value $\leq$ 0.05 in one of the *prakriti* group. For validation we have considered such differential methylated CpG islands.

The probe annotations from Agilent e-array technology file and analyzed manually using galaxy genome browser [13] into different genomic regions based on the distance from TSS of the gene. The probes were mapped to 5'-UTR and 3'-UTR regions using RefSeq gene coordinates. The enrichment of mPSRs at chromosomes, CpG Island, CpG shores and different genomic position was statistically analyzed by Chi-square test ($p < 0.05$). CpG shores are the regions which span 200 bp from either side of the CpG islands and the methylation variation at these sites showed attribute for the phenotypic variation. The annotation of whole genome CpG island microarray probes revealed that 87% (165,826) of the probes were associated with CpG islands and remaining 13% (33,573) were overlapping with CpG shores. Further to assess the crosstalk between the DNA methylation and histone modification EpiExplorer [14] has been used. The methylated promoter regions were fetched from DBTSS and TRED databases.

Table for prominent characteristic features of the three extreme *Prakriti* types: *Vata, Pitta,* and *Kapha*

**Main features**	**Features**	***Vata***	***Pitta***	***Kapha***
**Physical Appearance**	**Face**	Small, Irregular	Medium	Large, Attractive
	**Weight**	Lean	Average	Hefty
	**Structure**	Tall	Medium	Short, Stocky
	**Skin**	Rough tendency to darken	Smooth, Copper colored acne/Pimple	Large attractive thick eyebrows
	**Nails**	Rough, Dry, Irregular Cracked	Glossy copper colored	Long, white strong, thick smooth
	**Teeth**	Cracked, Irregular, Dull White	Moderate yellow plaques	Thick, Full, Glossy and Smooth
	**Hands**	Short	Medium	Long
	**Body hair**	Scanty, Irregular	Moderate Faintly colored	Plenty, Thick
	**Scalp Hair**	Rough, Dry, Irregular break	Scanty, Baldness and Graying	Plenty, Thick, Long and Glossy
	**Joints**	Prominent, Crackling Sound	Flabby	Strong well defined compact
	**Abdomen**	Thin	Medium	Large/Obese
	**Chest**	Thin and Prominent Ribs	Medium	Muscular strong
	**Tongue**	Dry and Dark spots	Thin copper colored	Clean, Thick
**Physical Characters**	**Strength**	Weak	Moderate	Strong
	**Vessels**	Prominent Reticulated	Less Prominent, Greenish	Deep seated
	**Temperat ure**	Cold less than normal temperature	Hot above normal	Normal
	**Pulse**	Irregular, Quick	Fast	Slow, Voluminous
	**Odor**	None	Foul Smell	Oily
	**Voice**	Fast, Irritating	Sharp Commanding	Soft, Sweet resonating
	**Sweating**	Seldom	Excessive foul odor	Slow Steady
	**Activities**	Quick activities	Moderate	Stagnant
**Metabolism**	**Digestion**	Irregular	Powerful	Weak
	**Hunger Control**	Irregular	Poor	Very Good
	**Thirst**	Irregular	Very often	Seldom
	**Food Quality**	Irregular Large and small	Heavy	Normal
	**Taste**	Sweet and salty	Sweet, Bitter astringent	Pungent, astringent bitter
	**Bowel**	Irregular	Loose, Some Solid	Regular, Well Formed
**Mental Characters**	**Grasping**	Very fast	Quick	Slow
	**Memory**	Irregular, Short term-Good, Long term-weak	Powerful	Short term-weak, Long term-strong
	**Emotions**	Wavering, Extreme happy or extreme sad	Angry	Calm Attracted
	**Sleep**	Interrupted, Less	Normal	Excessive deep
	**Dreams**	Flying, Climbing and Motion	Violence	Bodies of water and Romance
	**Sexual Desires**	Wavering	Moderate	Excessive
	**Speech**	Very fast, Stammering and Missing words	Sharp provocative	Slow, Resonant and Clear
	**Temperam ent**	Quick Lots of Initiative	Moderate	Very slow lethargic
	**Attitude**	Worrying, Instability	Angry, Easily Provoked	Peaceful, Slow, Steady

	**Food**	Warm	Cold	Moderate
	**Interests**	Travel	Sports/Politics	Relax, Water bodies, Flowering and Nature
	**Belief**	Radical changing	Goal oriented, Fanatics, Leaders	Loyal, Followers
	**Mind**	Quick adaptable	Penetrating Critical	Slow, Lethargic
**Diseases**	**Vitiated**	Emaciation, Black discoloration, desire for hot climate and hot substance, constipation, fatigue, insomnia, vertigo, pathetic look	Yellowish discoloration of stools, urine, skin and eyes, excessive hunger, polydipsia, insomnia, burning sensation	Indigestion, excessive salivation, laziness, feeling heaviness, feeling of coldness, dyspnea, cough,
	**Pathological manifestation**	Visceroptosis, dilation, numbness, gnawing pain, necrosis resulting in formation of holes, hyper aesthesis and tingling, developmental and neurological disorders, dementia	Burning sense, red discoloration, hyper metabolism, increased perspiration, Inflammation, Ulcers, gangrene, exhaustion, emaciation and fermentation	Excessive fat, swelling, indigestion, excessive sleep, white discoloration, Obesity, diabetes, atherosclerosis
	**Reduction**	Intolerance to sound, faintness, excessive sleep	Loss of appetite, coldness, fading of skin complexion	Vertigo, feeling as if chest and skull are vacant, palpitation, flaccidity

References

1. Patwardhan B, Mashelkar RA: **Traditional medicine-inspired approaches to drug discovery: can Ayurveda show the way forward?** *Drug Discov Today* 2009, **14:**804-811.

2. Valiathan M: *The Legacy of Caraka.* Chennai: Orient Blackswan; 2003.

3. Waterland RA, Kellermayer R, Laritsky E, Rayco-Solon P, Harris RA, Travisano M, Zhang W, Torskaya MS, Zhang J, Shen L: **Season of conception in rural gambia affects DNA methylation at putative human metastable epialleles.** *PLoS Genet* 2010, **6:**e1001252.

4. Borghol N, Suderman M, McArdle W, Racine A, Hallett M, Pembrey M, Hertzman C, Power C, Szyf M: **Associations with early-life socio-economic position in adult DNA methylation.** *Int J Epidemiol* 2012, **41:**62-74.

5. Rotti H, Raval R, Anchan S, Bellampalli R, Bhale S, Bharadwaj R, Bhat B, Dedge A, Dhumal V, Gangadharan G, et al: **Determinants of Prakriti, the Human Constitution Types of Indian Traditional Medicine and its Correlation with Contemporary Science.** *J Ayurveda Integr Med* 2014, **5:**167-175.

6. Caldecott T: *Ayurveda: The divine science of life.* Elsevier Health Sciences; 2006.

7. Keshet I, Schlesinger Y, Farkash S, Rand E, Hecht M, Segal E, Pikarski E, Young RA, Niveleau A, Cedar H: **Evidence for an instructive mechanism of de novo methylation in cancer cells.** *Nat Genet* 2006, **38:**149-153.

8. Weber M, Davies JJ, Wittig D, Oakeley EJ, Haase M, Lam WL, Schuebeler D: **Chromosome-wide and promoter-specific analyses identify sites of differential DNA methylation in normal and transformed human cells.** *Nat Genet* 2005, **37:**853-862.

9. Pälmke N, Santacruz D, Walter J: **Comprehensive analysis of DNA-methylation in mammalian tissues using MeDIP-chip.** *Methods* 2011, **53:**175-184.

10. Jia J, Pekowska A, Jaeger S, Benoukraf T, Ferrier P, Spicuglia S: **Assessing the efficiency and significance of Methylated DNA Immunoprecipitation (MeDIP) assays in using in vitro methylated genomic DNA.** *BMC Res Notes* 2010, **3:**240.

11. Yan PS, Efferth T, Chen H-L, Lin J, Rödel F, Fuzesi L, Huang TH-M: **Use of CpG island microarrays to identify colorectal tumors with a high degree of concurrent methylation.** *Methods* 2002, **27:**162-169.

12. Labonté B, Suderman M, Maussion G, Lopez JP, Navarro-Sánchez L, Yerko V, Mechawar N, Szyf M, Meaney MJ, Turecki G: **Genome-wide methylation changes in the brains of suicide completers.** *Am J Psychiatry* 2013, **170:**511-520.

13. Giardine B, Riemer C, Hardison RC, Burhans R, Elnitski L, Shah P, Zhang Y, Blankenberg D, Albert I, Taylor J: **Galaxy: a platform for interactive large-scale genome analysis.** *Genome Res* 2005, **15:**1451-1455.

14. Halachev K, Bast H, Albrecht F, Lengauer T, Bock C: **EpiExplorer: live exploration and global analysis of large epigenomic datasets.** *Genome Biol* 2012, **13:**R96.
