# Supplementary material for: DNA methylation analysis of phenotype specific stratified Indian population
Source: J Transl Med. 2015 May 8;13:151. doi: 10.1186/s12967-015-0506-0 (PMC4438459; doi:10.1186/s12967-015-0506-0)
Supplement: Additional file 2: Table S1. — Bisulfite Specific primers and PCR conditions. [file 12967_2015_506_MOESM2_ESM.pdf]

Table S2: Bisulfite Specific primers and PCR conditions

Gene Name	Status	Primer Sequence	Chromosomal Coordinates	PCR condition
NNAT **(neuronatin)**	Commonly Methylated	FP- 5' ATTTATTAGGGTTTGGGGG 3'<br>RP- 5' ATCATCTACCCCATAAAACAAA 3'	chr20:35,582,248-35,582,671	Step1- 95^0 C – 5'<br>Step2-95^0 C – 30<br>60^0 C – 1'<br>72^0 C – 1.30'<br>Step2 for 35 Cycle<br>Step3-72^0 C -10.00'
LHX1 **(LIM homeobox 1)**	Vata Methylated	FP- 5' AGTAGGAGGGGGTTTAGGTA 3'<br>RP- 5' TAAACTCTAAAAACCCCCTC 3'	chr17:32,367,978-32,368,193	Step1- 95^0 C – 5'<br>Step2-95^0 C – 30<br>60^0 C – 1'<br>72^0 C – 1.30'<br>Step2 for 35 Cycle<br>Step3-72^0 C -10.00'
SOX11 **(SRY (sex determining region Y)-box 11)**	Pitta Methylated	FP- 5' GGAGGTYGTYGTTTTAGGTT 3'<br>RP- 5' TTTTAAAAAATCCRCRTATATACAC 3'	chr2:5,749,505-5,749,799	Step1- 95^0 C – 5'<br>Step2-95^0 C – 30<br>57^0 C – 1'<br>72^0 C – 1.30'<br>Step2 for 35 Cycle<br>Step3-72^0 C -10.00'
CDH22 **(cadherin 22)**	Kapha and High BMI Methylated	FP- 5'GGTTGGGGTTTAGGAGTATG 3'<br>RP- 5'CTTCCCCRACTCTAAAACTATC 3'	chr20:44,313,355-44,313,655	Step1- 95^0 C – 5'<br>Step2-95^0 C – 30<br>60^0 C – 1'<br>72^0 C – 1.30'<br>Step2 for 35 Cycle<br>Step3-72^0 C -10.00'
