# Supplementary figures and images for: DNA methylation analysis of phenotype specific stratified Indian population
Source: J Transl Med. 2015 May 8;13:151. doi: 10.1186/s12967-015-0506-0 (PMC4438459; doi:10.1186/s12967-015-0506-0)

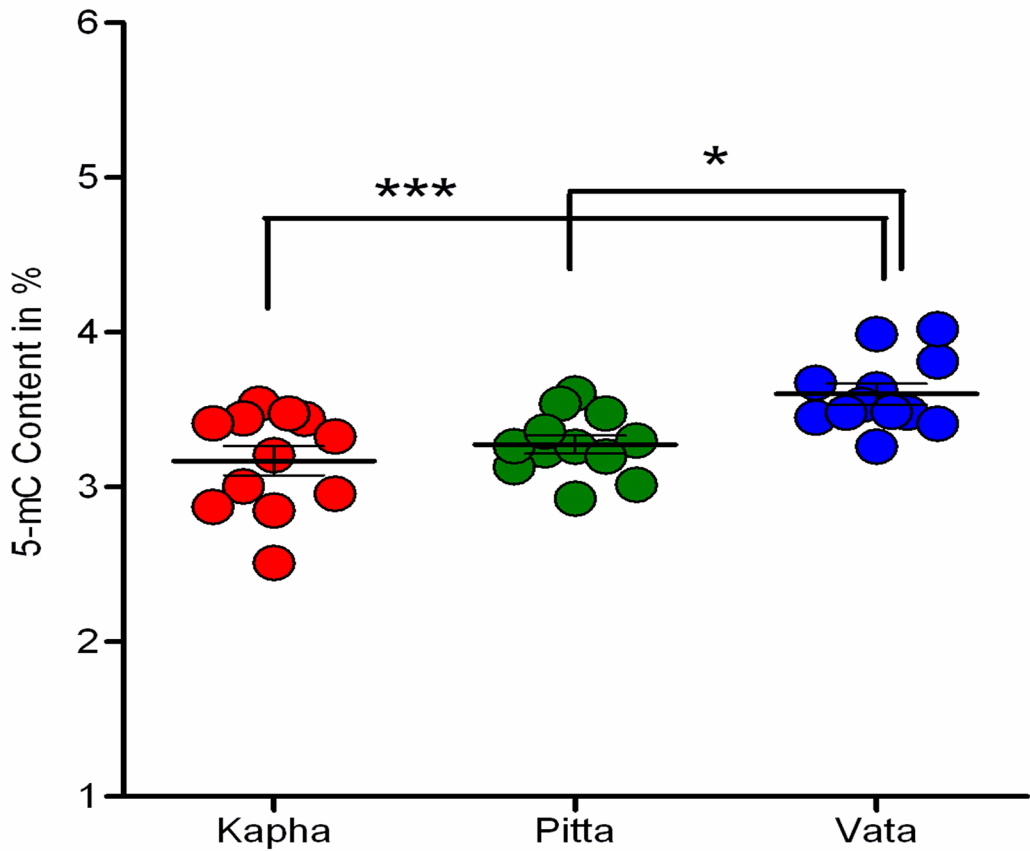

Supplement: Additional file 3: Figure S1. — Global methylation estimation (5-mC) using RP-HPLC method. Each dot represents the total methylation cytosine content of the individual prakriti sample. Within the prakriti groups mean ± standard error was shown in black lines. The aster sign indicates the P-value significance. ***p≤ 0.01, **p≤ 0.05. [file 12967_2015_506_MOESM3_ESM.pdf]

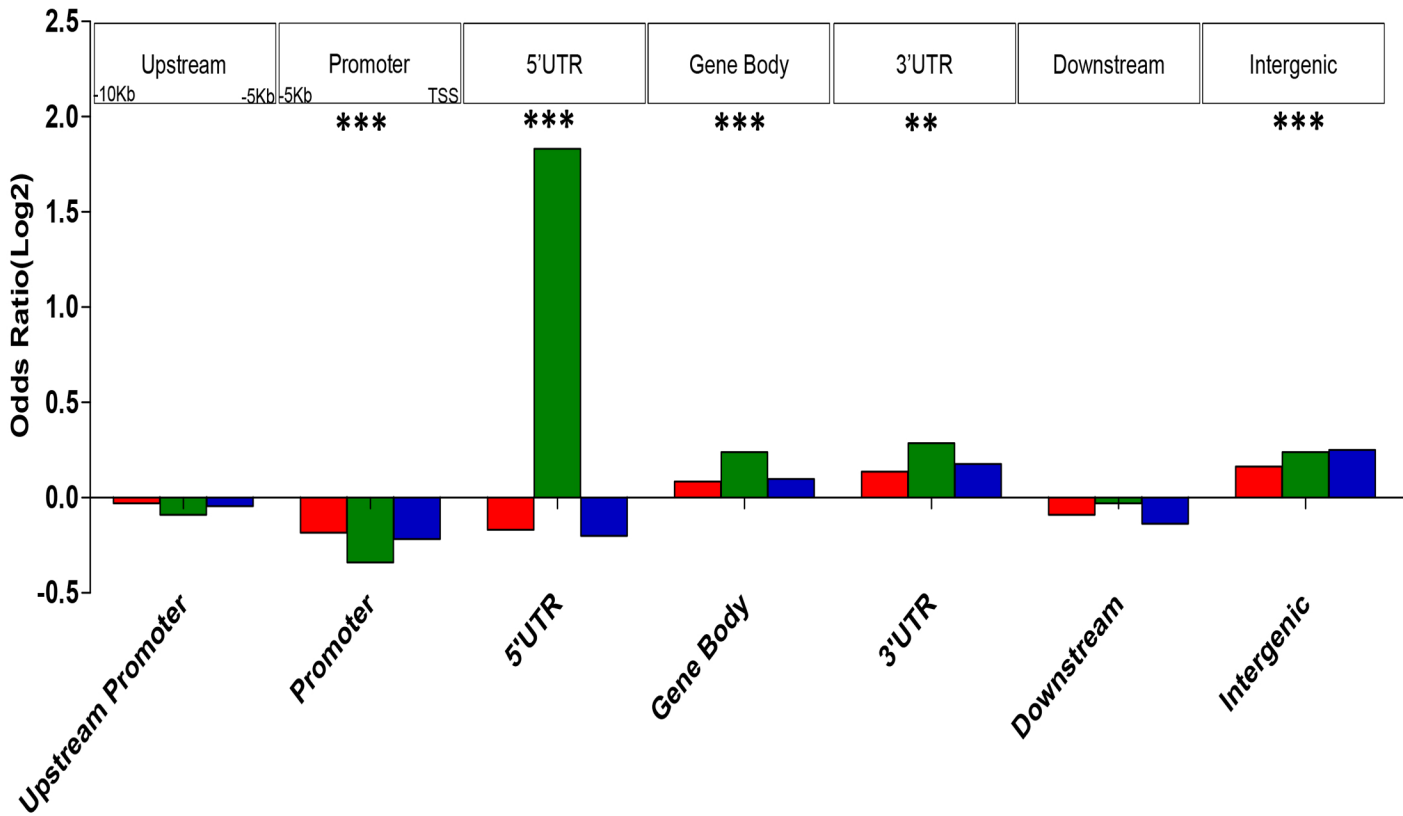

Supplement: Additional file 4: Figure S2. — Enrichment analysis of methylated prakriti associated regions in different genomic regions. Distribution of different genomic regions categorized based on the distance from TSS. Chi-square test was performed for the significance p ≤ 0.001 shown in three asters and p ≤ 0.01 shown in two aster signs respectively. [file 12967_2015_506_MOESM4_ESM.pdf]

**A**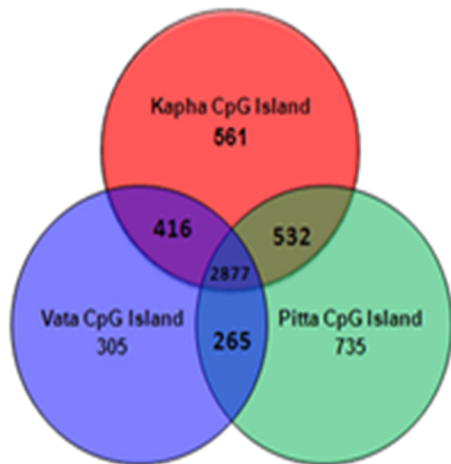**B**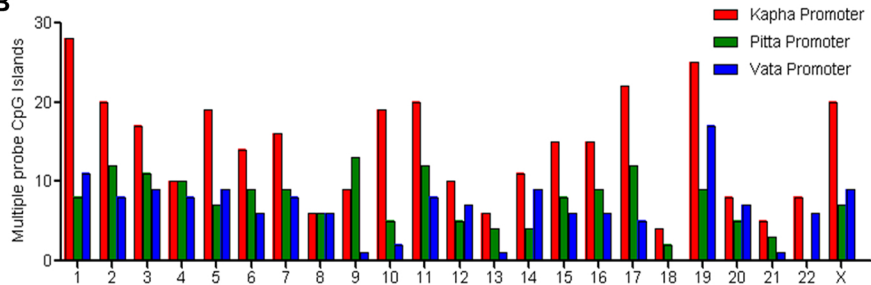**C**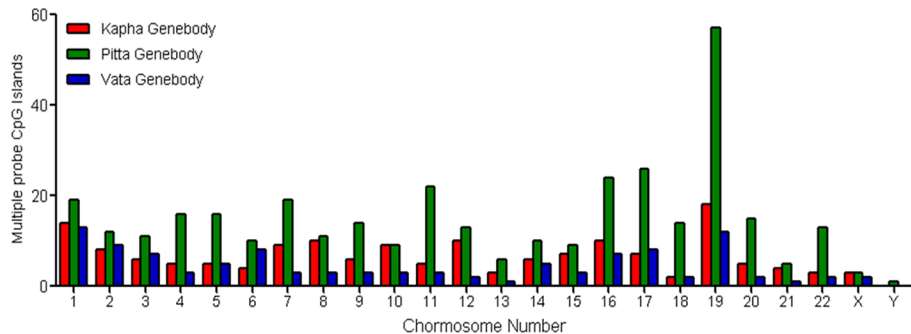

Supplement: Additional file 5: Figure S3. — Distribution of CpG islands represented with two or more significantly methylated probes in three prakriti. (A) Venn diagrams showing uniquely methylated CpG island represented by two or more significantly methylated probes. (B) and (C) is the distribution of identified unique CpG islands in promoter ( −10kb to +500bp) and gene body (within genic coordinates as per RefSeq gene coordinates). The promoter specific methylated CpG islands are more represented in Kapha prakriti and gene body associated CpG islands in Pitta prakriti. [file 12967_2015_506_MOESM5_ESM.pdf]

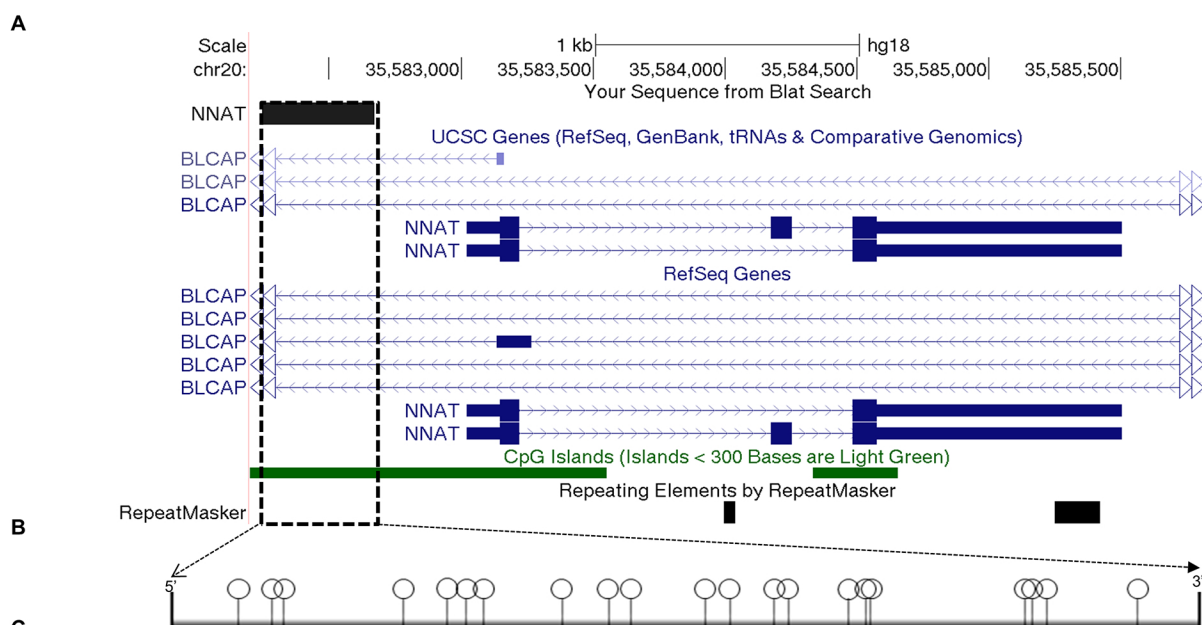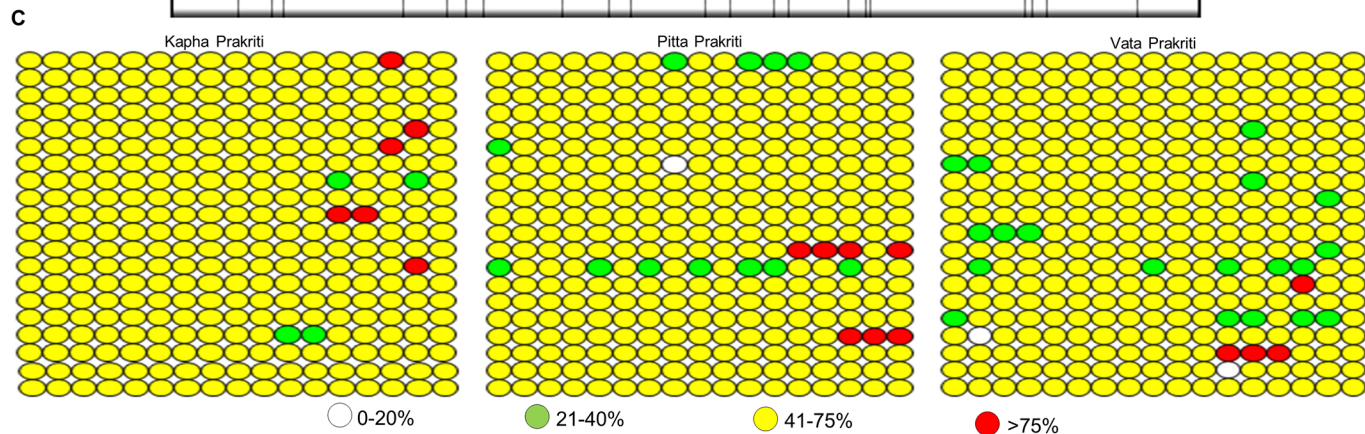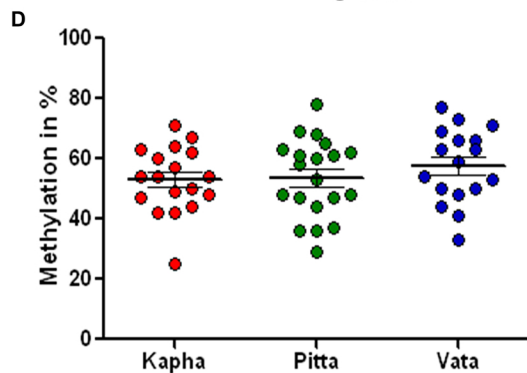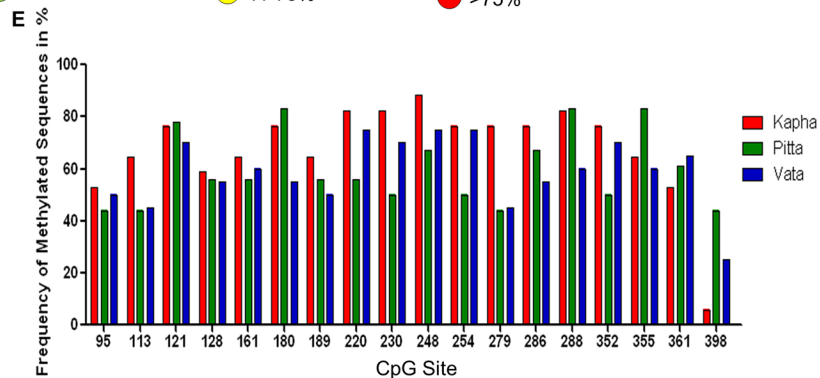

Supplement: Additional file 6: Figure S4. — Bisulfite genome sequence analysis of NNAT promoter, a commonly methylated region. (A) Genomic location of the region selected for bisulfite specific PCR. (B) The individual CpG sites and its position in NNAT promoter region is shown. (C) Individual CpG sites absolute methylation in three prakriti. The color indicates the extent of methylation at individual CpG sites. (D) Overall methylation of the whole amplicon in individuals represented by mean ± standard error. (E) Methylation level at individual CpG sites represented by mean ± standard error. [file 12967_2015_506_MOESM6_ESM.pdf]

**A**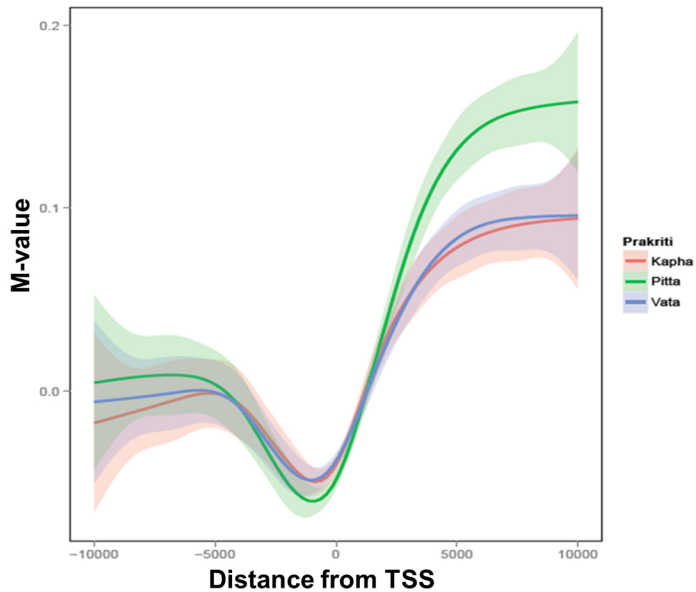**B**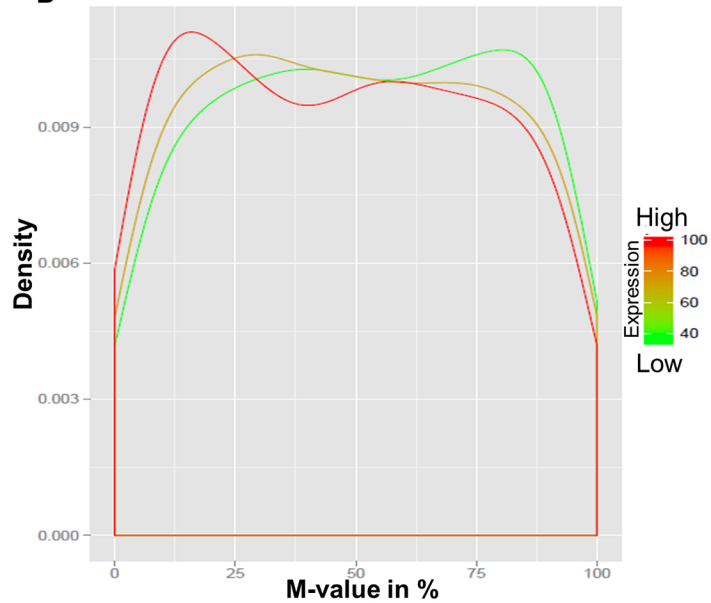

Supplement: Additional file 7: Figure S5. — Whole blood DNA methylation profile and its correlation with whole blood gene expression profile. (A) Variation in M-values with respect to distance from TSS. All significant methylated probes of within prakriti analysis were smoothened and represented in lines and the spread in the color to indicate the range of M-values in the prakriti. (B) Correlation analysis between M-value and whole blood expression. Whole blood expression values downloaded from BioGPS website and respective promoter associated genes M-values were converted to percentile values which is indicative of a fair negative correlation between DNA methylation and gene expression. [file 12967_2015_506_MOESM7_ESM.pdf]

**A**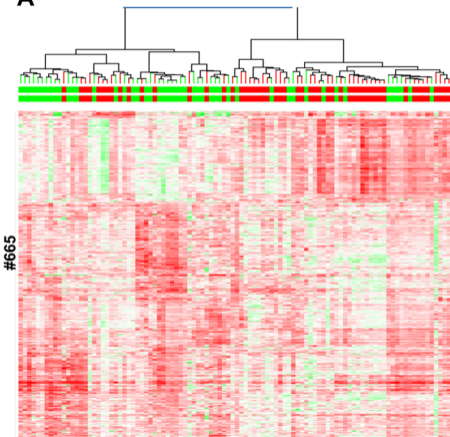**B**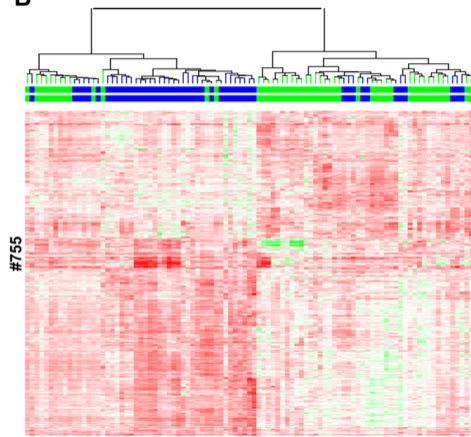**C**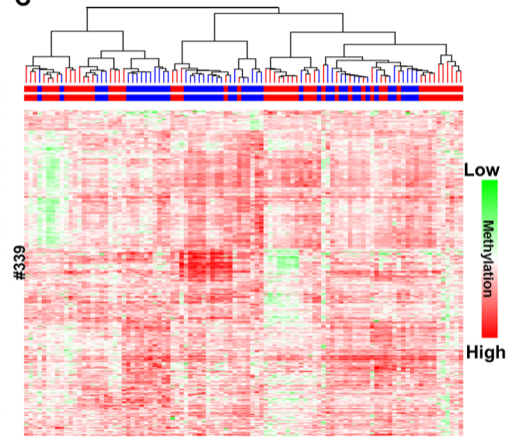**Kapha****Pitta****Vata****Low****Methylation****High**

Supplement: Additional file 8: Figure S6. — Heat map and clustering of prakriti differential methylation analysis (A), (B) and (C) are the supervised cluster analysis of significant (p≤0.2) identified specific to CpG loci, co-segregate as specific cluster in Kapha vs. Pitta, Pitta vs. Vata and Vata vs. Kapha respectively. Distinct co-segregation of differential methylated CpG sites in a prakriti specific manner was identified. [file 12967_2015_506_MOESM8_ESM.pdf]

**A**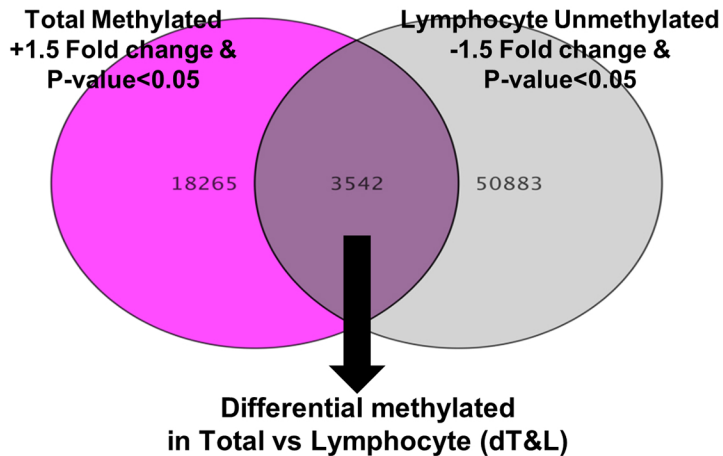**B**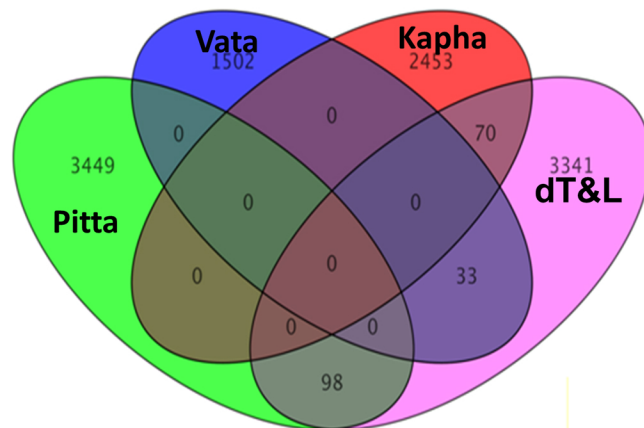**C**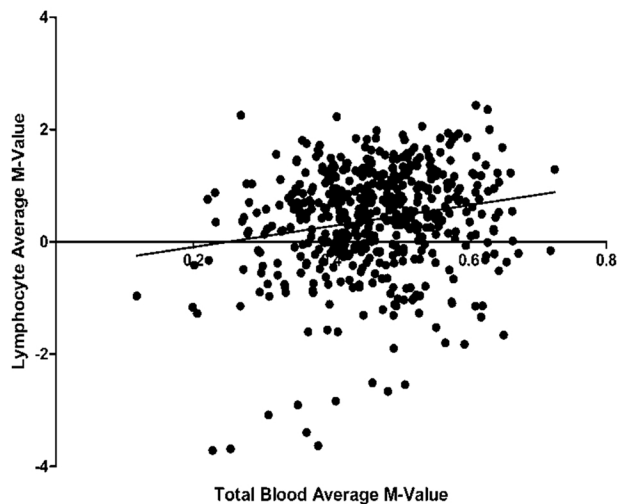

Supplement: Additional file 12: Figure S7. — Comparison analysis of methylation profiles of total blood (n=147) and lymphocyte (n=11) MeDIP microarray (A) Venn diagram representing differentially methylated probes (#3542) among total blood and lymphocyte DNA MeDIP microarray data. The significant methylated probes (p≤ 0.05 and Fold change of ≥1.5) of was assessed with the significant Unmethylated probes (p≤ 0.05 and Fold change of ≤−1.5) of lymphocyte. Nearly 16% of variation in methylation was observed between two different type of data. (B) Uniquely represented prakriti methylated probes form inter-prakriti analysis compared with differential methylated probes. The observed variable number of probes was very minimal. (C) Correlation analysis of M-values for the mPSRs among total blood and lymphocyte microarray data. The distribution and positive correlation with Spearman correlation R=0.15 was observed to be significant at p = 0.0004. [file 12967_2015_506_MOESM12_ESM.pdf]

#3989

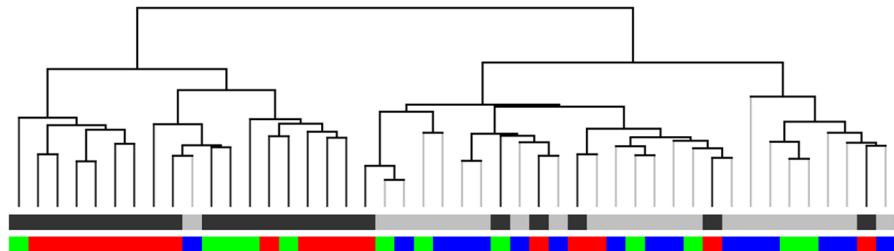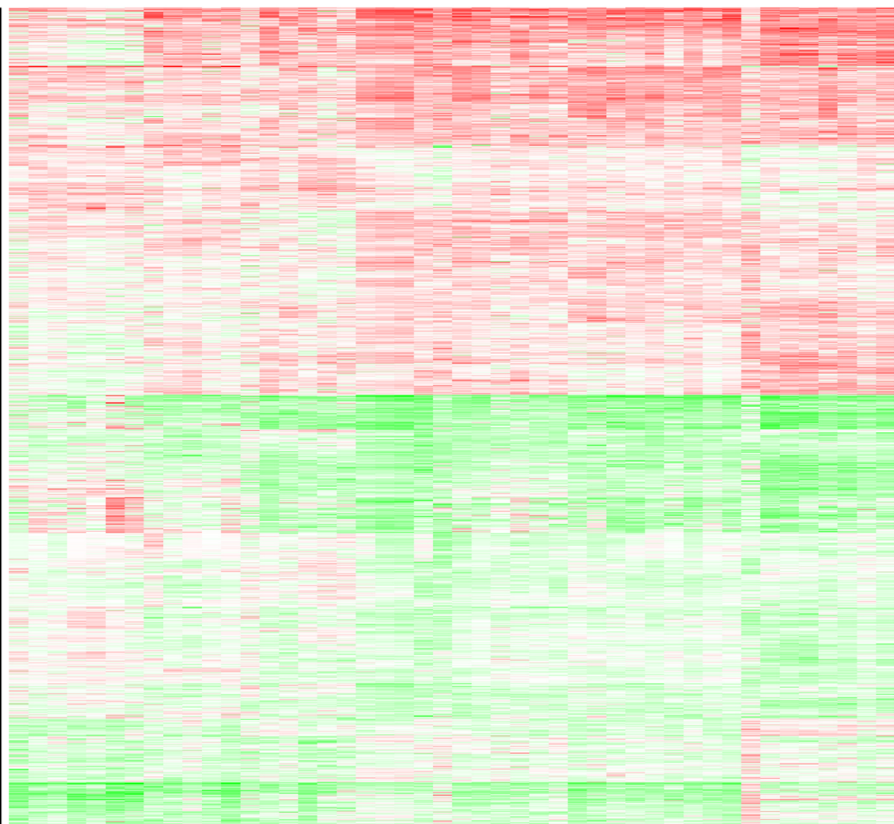

Low  
Methylation  
High

■ Kapha ■ Pitta ■ Vata ■ High BMI ■ Low BMI

Supplement: Additional file 13: Figure S8. — Association of BMI and prakriti. Hierarchical cluster analysis of significant differentially methylated probes in high BMI and low BMI. The rows represent the probes and columns represent the arrays with BMI and prakriti phenotype. [file 12967_2015_506_MOESM13_ESM.pdf]

A

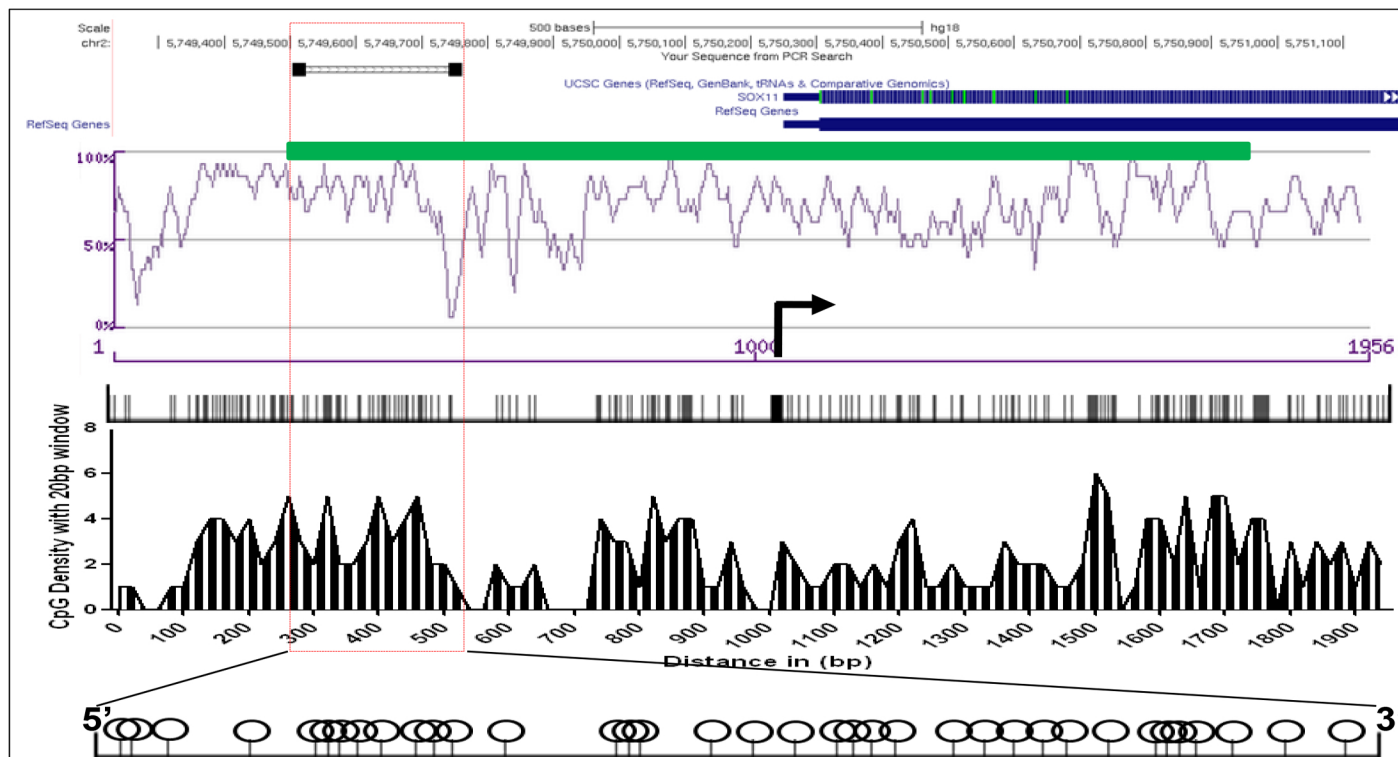

B

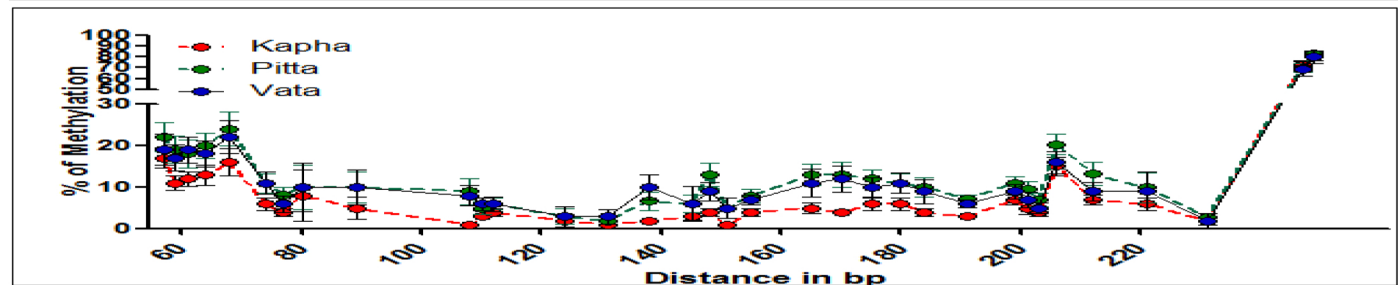

C

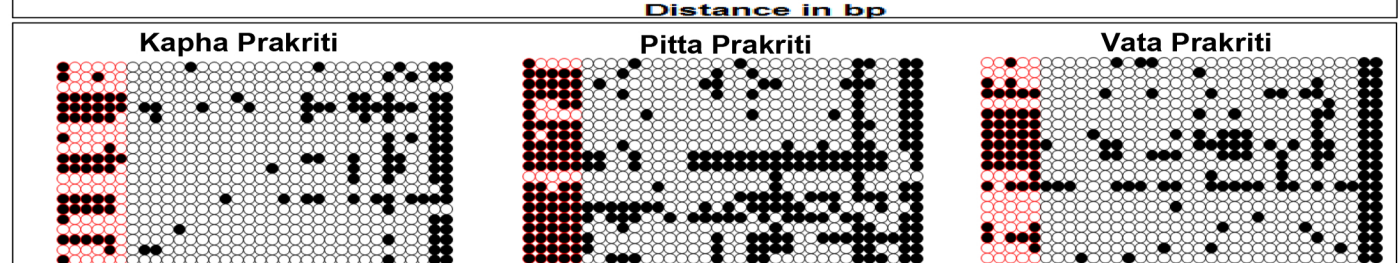

D

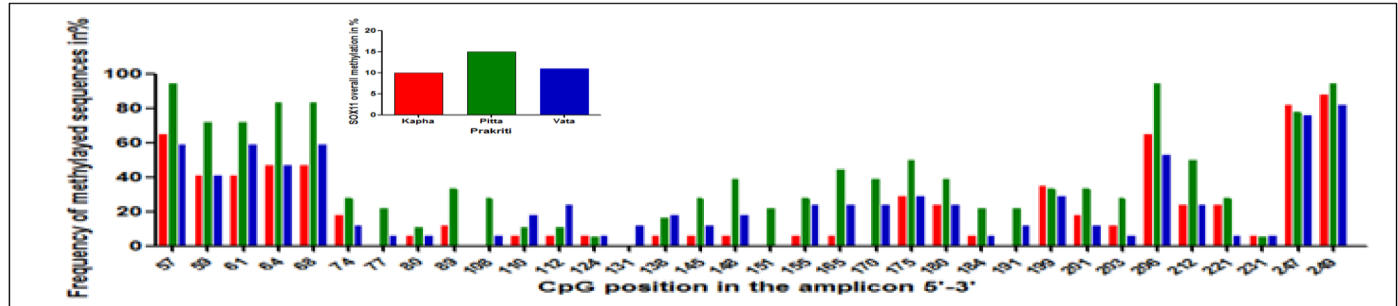

Supplement: Additional file 16: Figure S10. — Overview of methylation status of SOX11 in prakriti. A: Genomic position of amplicon, GC ratio and CpG density is shown at the top of the graphs. Green bar represents the CpG Island and arrow indicates the position of transcription start site. CpG density across a window size of 20bp is drawn. The red dotted box represents the amplicon for which bisulfite specific sequencing was performed. B: Average absolute methylation at individual CpG sites in three prakriti. The significant difference in methylation across CpG site was evaluated by two-way ANOVA and aster sign was represented for the significant (p < 0.05) CpG sites. C: Absolute methylation in individual CpG sites across three prakriti. A methylation cut off >15% was considered to call methylated sites and are represented in black circles. The circles in white were considered as unmethylated with respect to the cut-off given. The significant differentially methylated region is shown in red bordered circles. D: Quantitation of methylated CpG sites in the differentially methylated region with the same cut-off, highlighting the CpG sites with higher frequency of methylation in Vata prakriti. Cumulative methylation analysis for region of interest by averaging all CpG sites in the prakriti population showed higher level of methylation in Pitta as compared to other prakriti. [file 12967_2015_506_MOESM16_ESM.pdf]
