# Supplementary material for: DNA methylation analysis of phenotype specific stratified Indian population
Source: J Transl Med. 2015 May 8;13:151. doi: 10.1186/s12967-015-0506-0 (PMC4438459; doi:10.1186/s12967-015-0506-0)
Supplement: Additional file 9: Table S2. — List of 501mPSRs. [file 12967_2015_506_MOESM9_ESM.pdf]

Table S2: List of 501mPSRs.

ProbeName	p (Corr)	FC K vs P	FC V vs K	FC P vs V	Gene Symbol	Probe Coordinates	CpG Island	Prakriti More Methylated
**A_17_P00846129**	0.14	1.36	-1.78	1.31		chr1:226741256-226741315	chr1:226740969-226742375	Kapha
**A_17_P16504768**	0.19	1.26	-1.21	-1.04	CADM1	chr11:114880191-114880235	chr11:114878784-114881091	Kapha
**A_17_P07740806**	0.15	1.49	-1.39	-1.07	LMO2	chr11:33848096-33848155	#N/A	Kapha
**A_17_P16475089**	0.17	1.31	-1.10	-1.19	WNT11	chr11:75597316-75597360	chr11:75596389-75599732	Kapha
**A_17_P08336158**	0.14	1.33	-1.01	-1.32	COL2A1	chr12:46684852-46684896	chr12:46684156-46684998	Kapha
**A_17_P09319924**	0.19	1.29	-1.08	-1.19	ACTR10	chr14:57736659-57736703	chr14:57736320-57736951	Kapha
**A_17_P09673912**	0.17	1.52	-1.25	-1.21	NEDD4	chr15:54072461-54072520	#N/A	Kapha
**A_17_P09892937**	0.14	1.29	-1.05	-1.23	ZNF213	chr16:3139573-3139622	#N/A	Kapha
**A_17_P15238970**	0.14	1.27	-1.15	-1.11	SIX3	chr2:45023378-45023422	chr2:45023009-45025388	Kapha
**A_17_P11119673**	0.18	1.34	-1.20	-1.12	NXT1	chr20:23279261-23279305	chr20:23278636-23280046	Kapha
**A_17_P11190515**	0.16	1.21	-1.27	1.05	CDH22	chr20:44313625-44313669	chr20:44313106-44313695	Kapha
**A_17_P15621436**	0.18	1.38	-1.16	-1.19	GAB1	chr4:144477270-144477314	chr4:144476381-144477977	Kapha
**A_17_P03200932**	0.18	1.82	-1.21	-1.51	CNOT6L	chr4:78958631-78958690	#N/A	Kapha
**A_17_P15754367**	0.18	1.20	-1.11	-1.09	HAND1	chr5:153837838-153837882	chr5:153837231-153839306	Kapha
**A_17_P15126949**	0.15	1.24	-1.02	-1.22	BOLA1	chr1:148138518-148138562	chr1:148137703-148138570	Kapha
**A_17_P15162325**	0.11	1.21	1.01	-1.21	ARL8A	chr1:200380701-200380746	chr1:200379774-200380766	Kapha
**A_17_P00737825**	0.20	1.39	-1.04	-1.33	PRELP	chr1:201723448-201723492	chr1:201723407-201723682	Kapha
**A_17_P16910769**	0.16	1.23	-1.08	-1.13	FLJ11171	chr16:69880674-69880718	chr16:69880616-69881015	Kapha
**A_17_P10193689**	0.15	1.23	-1.04	-1.19	SMG6	chr17:2153411-2153455	chr17:2153267-2153998	Kapha
**A_17_P10310314**	0.19	1.28	1.01	-1.30		chr17:33838495-33838539	chr17:33837946-33838979	Kapha
**A_17_P17284236**	0.17	1.24	-1.07	-1.16	SYNGR1	chr22:38076505-38076549	chr22:38075929-38076580	Kapha
**A_17_P04299971**	0.16	1.39	-1.73	1.24	H2AFY	chr5:134762401-134762456	chr5:134761995-134763160	Kapha
**A_17_P16119569**	0.15	1.14	-1.21	1.07	STK3	chr8:99906957-99907001	chr8:99906637-99907112	Kapha
**A_17_P00001114**	0.12	-1.35	1.22	1.11		chr1:1090371-1090416	chr1:1087906-1090447	Pitta
**A_17_P15009603**	0.17	-1.43	1.19	1.20	C1orf70	chr1:1463095-1463139	chr1:1462970-1463205	Pitta
**A_17_P00524032**	0.12	-1.37	1.20	1.14	PKLR	chr1:153530892-153530940	#N/A	Pitta
**A_17_P00530664**	0.18	-1.43	1.37	1.04	PEAR1	chr1:155145339-155145383	#N/A	Pitta
**A_17_P00543247**	0.15	-1.44	1.02	1.42	IGSF9	chr1:158166102-158166146	chr1:158166056-158166477	Pitta
**A_17_P15138082**	0.19	-1.47	1.13	1.30	UAP1	chr1:160798275-160798319	chr1:160797480-160798477	Pitta
**A_17_P00567027**	0.11	-1.43	1.09	1.31	LMX1A	chr1:163471724-163471774	chr1:163471125-163472131	Pitta
**A_17_P15160669**	0.12	-1.23	-1.01	1.24	NR5A2	chr1:198383747-198383791	chr1:198383319-198383827	Pitta
**A_17_P00004298**	0.18	-1.24	1.15	1.08	SKI	chr1:2228551-2228599	chr1:2226302-2228929	Pitta
**A_17_P15011753**	0.15	-1.26	-1.02	1.29	PEX10	chr1:2333600-2333644	chr1:2333245-2334577	Pitta
**A_17_P00928574**	0.15	-1.32	-1.02	1.34	ZNF496	chr1:245530957-245531001	chr1:245530471-245531333	Pitta
**A_17_P00933088**	0.12	-1.34	1.09	1.23	ZNF672	chr1:247108287-247108332	chr1:247108186-247109419	Pitta
**A_17_P00933090**	0.07	-1.24	-1.06	1.31	ZNF672	chr1:247108836-247108880	chr1:247108186-247109419	Pitta

ProbeName	p (Corr)	FC K vs P	FC V vs K	FC P vs V	Gene Symbol	Probe Coordinates	CpG Island	Prakriti More Methylated
**A_17_P00096245**	0.18	-1.53	1.27	1.21	RUNX3	chr1:25130245-25130292	chr1:25128114-25131592	Pitta
**A_17_P15015273**	0.14	-1.47	1.27	1.15	MEGF6	chr1:3490389-3490433	#N/A	Pitta
**A_17_P00166514**	0.13	-1.33	-1.02	1.37	TIE1	chr1:43543507-43543552	#N/A	Pitta
**A_17_P15021110**	0.10	-1.19	-1.08	1.29	RERE	chr1:8686037-8686081	chr1:8685592-8686136	Pitta
**A_17_P15007146**	0.18	-1.33	1.10	1.21	AGRN	chr1:968669-968713	chr1:965359-970168	Pitta
**A_17_P07424748**	0.18	-1.41	1.31	1.08		chr10:101271423-101271467	chr10:101271171-101272106	Pitta
**A_17_P07424753**	0.15	-1.33	1.03	1.29		chr10:101271995-101272039	chr10:101271171-101272106	Pitta
**A_17_P16293228**	0.19	-1.26	1.06	1.19	FRMD4A	chr10:13738976-13739020	chr10:13738568-13739546	Pitta
**A_17_P07091300**	0.13	-1.62	1.21	1.34	C10orf38	chr10:15294762-15294810	chr10:15294604-15294858	Pitta
**A_17_P16305236**	0.13	-1.23	1.11	1.11	WAC	chr10:28862311-28862355	chr10:28861195-28863098	Pitta
**A_17_P07201551**	0.17	-1.35	1.04	1.30	GDF10	chr10:48049501-48049552	#N/A	Pitta
**A_17_P07997664**	0.19	-1.31	1.11	1.17	DDI1	chr11:103412884-103412928	chr11:103412615-103413200	Pitta
**A_17_P07997665**	0.19	-1.39	1.12	1.24	DDI1	chr11:103412956-103413003	chr11:103412615-103413200	Pitta
**A_17_P07594600**	0.19	-1.40	1.03	1.36	PKP3	chr11:390092-390136	chr11:390096-390757	Pitta
**A_17_P07824609**	0.08	-1.54	1.00	1.54		chr11:60171510-60171566	#N/A	Pitta
**A_17_P16459875**	0.19	-1.47	1.23	1.19	RTN3	chr11:63286681-63286732	chr11:63286464-63288410	Pitta
**A_17_P07836887**	0.18	-1.36	1.20	1.14	MACRO D1	chr11:63523032-63523076	chr11:63522634-63525242	Pitta
**A_17_P16469119**	0.19	-1.19	-1.17	1.40	PPFIA1	chr11:69794649-69794693	chr11:69793885-69795145	Pitta
**A_17_P07880572**	0.14	-1.46	1.35	1.08	FLJ33790	chr11:74814331-74814378	chr11:74814055-74814337	Pitta
**A_17_P08615336**	0.14	-1.33	1.12	1.19	CUX2	chr12:110232793-110232840	#N/A	Pitta
**A_17_P08615357**	0.13	-1.57	1.27	1.23	CUX2	chr12:110242856-110242900	chr12:110242171-110242904	Pitta
**A_17_P16596446**	0.12	-1.07	-1.32	1.41	KIAA185 3	chr12:118076423-118076467	chr12:118076239-118076559	Pitta
**A_17_P08656985**	0.18	-1.36	1.13	1.20		chr12:120750022-120750068	chr12:120749757-120750337	Pitta
**A_17_P08686329**	0.09	-1.75	1.17	1.50	GLT1D1	chr12:127995802-127995856	#N/A	Pitta
**A_17_P08152900**	0.18	-1.37	1.04	1.32	CACNA2 D4	chr12:1844712-1844762	chr12:1844000-1845219	Pitta
**A_17_P16523230**	0.16	-1.32	1.06	1.25	CACNA2 D4	chr12:1844899-1844944	chr12:1844000-1845219	Pitta
**A_17_P08375769**	0.13	-1.19	-1.27	1.51	NAB2	chr12:55768480-55768524	chr12:55767064-55769383	Pitta
**A_17_P08378773**	0.16	-1.31	1.16	1.13	CENTG1	chr12:56406914-56406958	chr12:56406176-56407818	Pitta
**A_17_P09137227**	0.16	-1.28	-1.01	1.30		chr13:110543469-110543513	chr13:110543494-110543698	Pitta
**A_17_P09145246**	0.15	-1.54	1.20	1.28	ATP11A	chr13:112473149-112473201	#N/A	Pitta
**A_17_P16673700**	0.09	-1.52	1.18	1.29		chr13:112600395-112600450	chr13:112600382-112600774	Pitta
**A_17_P16674248**	0.08	-1.24	1.11	1.11	MCF2L	chr13:112798471-112798515	chr13:112798487-112799566	Pitta
**A_17_P08706322**	0.09	-1.42	1.13	1.26		chr13:19290595-19290639	chr13:19290430-19290707	Pitta
**A_17_P08708559**	0.08	-1.45	1.19	1.22	CRYL1	chr13:19887090-19887134	chr13:19887007-19887836	Pitta
**A_17_P09520408**	0.19	-1.29	1.02	1.25		chr14:101171025-101171069	chr14:101170918-101171349	Pitta
**A_17_P09521535**	0.12	-1.50	1.26	1.20	DYNC1H 1	chr14:101491870-101491922	chr14:101491904-101492109	Pitta
**A_17_P09522590**	0.13	-1.44	1.16	1.24	WDR20	chr14:101750198-101750242	chr14:101750040-101750272	Pitta

ProbeName	p (Corr)	FC K vs P	FC V vs K	FC P vs V	Gene Symbol	Probe Coordinates	CpG Island	Prakriti More Methylated
**A_17_P09523668**	0.14	-1.19	-1.12	1.34	ANKRD9	chr14:102043809-102043853	chr14:102043070-102046419	Pitta
**A_17_P09477529**	0.17	-1.17	-1.04	1.22	RIN3	chr14:92224541-92224587	#N/A	Pitta
**A_17_P09546690**	0.19	-1.43	1.05	1.37	ATP10A	chr15:23532244-23532293	chr15:23532269-23532485	Pitta
**A_17_P09556472**	0.19	-1.30	-1.02	1.32	OCA2	chr15:25922652-25922696	chr15:25922164-25922761	Pitta
**A_17_P16796512**	0.15	-1.30	1.23	1.06		chr15:60303931-60303975	chr15:60303616-60304097	Pitta
**A_17_P16813767**	0.09	-1.49	-1.03	1.53	MEX3B	chr15:80126104-80126148	chr15:80122527-80126992	Pitta
**A_17_P09926896**	0.11	-1.25	1.01	1.23	CLEC16 A	chr16:11179857-11179902	chr16:11179747-11180004	Pitta
**A_17_P09932325**	0.10	-1.31	1.15	1.14	SNX29	chr16:12570069-12570113	chr16:12569863-12570178	Pitta
**A_17_P09885810**	0.18	-1.17	-1.05	1.24	BAIAP3	chr16:1338140-1338184	chr16:1337681-1338246	Pitta
**A_17_P09885980**	0.15	-1.46	1.35	1.09	UNKL	chr16:1375095-1375152	#N/A	Pitta
**A_17_P09886266**	0.17	-1.31	1.20	1.09	CLCN7	chr16:1447656-1447700	#N/A	Pitta
**A_17_P09886437**	0.11	-1.31	1.06	1.24	TELO2	chr16:1490627-1490671	chr16:1490145-1490830	Pitta
**A_17_P09881541**	0.05	-1.47	-1.01	1.48	AXIN1	chr16:350674-350718	chr16:349985-351492	Pitta
**A_17_P09998228**	0.13	-1.35	1.12	1.20		chr16:47088118-47088162	chr16:47088036-47088310	Pitta
**A_17_P09900174**	0.18	-1.46	1.16	1.26	PPL	chr16:4873755-4873799	chr16:4873624-4874564	Pitta
**A_17_P10041354**	0.15	-1.28	-1.08	1.38	ZNF319	chr16:56588771-56588815	chr16:56587715-56589134	Pitta
**A_17_P09882690**	0.13	-1.41	1.19	1.18	RAB40C	chr16:617462-617506	chr16:617368-618129	Pitta
**A_17_P10117975**	0.10	-1.34	1.30	1.03	CHST5	chr16:74120671-74120715	chr16:74120614-74121613	Pitta
**A_17_P16920147**	0.13	-1.46	1.09	1.34	KCNG4	chr16:82813985-82814030	#N/A	Pitta
**A_17_P10174435**	0.08	-1.36	1.22	1.11	FBXO31	chr16:85925280-85925324	chr16:85925035-85925352	Pitta
**A_17_P16924284**	0.17	-1.22	-1.03	1.25		chr16:86142149-86142193	chr16:86141860-86142211	Pitta
**A_17_P16926058**	0.16	-1.63	1.32	1.24		chr16:86722181-86722240	#N/A	Pitta
**A_17_P10178215**	0.16	-1.36	1.10	1.23	ZFPM1	chr16:87067937-87067987	chr16:87067361-87068033	Pitta
**A_17_P10178491**	0.04	-1.41	-1.07	1.51	ZFPM1	chr16:87126578-87126622	chr16:87125948-87126663	Pitta
**A_17_P09883933**	0.16	-1.50	1.20	1.24	LMF1	chr16:899622-899667	#N/A	Pitta
**A_17_P10256655**	0.10	-1.29	1.05	1.24	RAI1	chr17:17654371-17654415	chr17:17653636-17655181	Pitta
**A_17_P10191987**	0.18	-1.26	-1.04	1.31	RTN4RL 1	chr17:1787236-1787280	chr17:1786739-1787959	Pitta
**A_17_P16935789**	0.11	-1.38	-1.05	1.44	CLUH	chr17:2548509-2548557	chr17:2547725-2548730	Pitta
**A_17_P10284899**	0.16	-1.27	-1.06	1.34	COPRS	chr17:27209925-27209969	chr17:27209877-27210693	Pitta
**A_17_P10312245**	0.19	-1.48	1.13	1.31	LASP1	chr17:34328626-34328679	#N/A	Pitta
**A_17_P10490911**	0.13	-1.36	1.12	1.22	KIAA130 3	chr17:76536609-76536653	chr17:76536492-76536828	Pitta
**A_17_P10492548**	0.12	-1.30	-1.12	1.45	SLC38A1 0	chr17:76870672-76870716	chr17:76870555-76870918	Pitta
**A_17_P10495052**	0.15	-1.32	1.04	1.27	FASN	chr17:77630413-77630457	chr17:77630012-77630763	Pitta
**A_17_P17029220**	0.13	-1.56	1.26	1.23	FOXK2	chr17:78152456-78152500	chr17:78152314-78152739	Pitta
**A_17_P10499106**	0.20	-1.23	-1.01	1.24	B3GNTL 1	chr17:78609289-78609333	#N/A	Pitta
**A_17_P17040986**	0.19	-1.38	1.14	1.21		chr18:12902276-12902320	chr18:12901024-12902704	Pitta
**A_17_P10832406**	0.18	-1.23	1.19	1.04	ZNF236	chr18:72735973-72736017	chr18:72735931-72736156	Pitta

ProbeName	p (Corr)	FC K vs P	FC V vs K	FC P vs V	Gene Symbol	Probe Coordinates	CpG Island	Prakriti More Methylated
**A_17_P10840518**	0.18	-1.27	1.05	1.21		chr18:74224802-74224846	chr18:74224600-74224914	Pitta
**A_17_P10844231**	0.17	-1.48	1.10	1.34	SALL3	chr18:74854933-74854977	chr18:74852927-74856537	Pitta
**A_17_P10846177**	0.17	-1.22	-1.02	1.24	NFATC1	chr18:75272140-75272184	chr18:75271415-75272433	Pitta
**A_17_P17088715**	0.10	-1.34	1.12	1.20	NFATC1	chr18:75336811-75336855	chr18:75334314-75337386	Pitta
**A_17_P10848549**	0.15	-1.31	1.21	1.09	KCNG2	chr18:75760613-75760657	chr18:75759900-75760988	Pitta
**A_17_P17089734**	0.08	-1.28	-1.05	1.34	KCNG2	chr18:75760694-75760738	chr18:75759900-75760988	Pitta
**A_17_P10537274**	0.18	-1.35	1.14	1.18		chr18:8649730-8649779	#N/A	Pitta
**A_17_P10537880**	0.13	-1.28	1.04	1.23	KIAA080 2	chr18:8774459-8774503	chr18:8774147-8774583	Pitta
**A_17_P10853675**	0.18	-1.21	1.01	1.20	MUM1	chr19:1328846-1328890	chr19:1328844-1329266	Pitta
**A_17_P10890888**	0.16	-1.32	1.14	1.16	NANOS3	chr19:13845945-13845989	#N/A	Pitta
**A_17_P17112142**	0.19	-1.25	-1.04	1.30		chr19:14237746-14237790	chr19:14237688-14237961	Pitta
**A_17_P10901468**	0.11	-1.25	-1.01	1.27	NR2F6	chr19:17216338-17216382	chr19:17216181-17217471	Pitta
**A_17_P17115989**	0.16	-1.26	-1.01	1.27	UNC13A	chr19:17659722-17659766	chr19:17659679-17660329	Pitta
**A_17_P10855914**	0.20	-1.31	1.02	1.28	BTBD2	chr19:1936899-1936943	chr19:1936880-1937198	Pitta
**A_17_P10857699**	0.19	-1.14	-1.13	1.28	GNG7	chr19:2462720-2462764	chr19:2462392-2462782	Pitta
**A_17_P10859732**	0.08	-1.32	-1.02	1.35	NCLN	chr19:3158985-3159029	chr19:3158920-3159157	Pitta
**A_17_P10939464**	0.20	-1.36	1.25	1.09	WDR88	chr19:38315122-38315166	#N/A	Pitta
**A_17_P10950180**	0.10	-1.39	1.03	1.35	TBCB	chr19:41296433-41296485	chr19:41296199-41298746	Pitta
**A_17_P17137904**	0.09	-1.31	1.09	1.19	LRFN1	chr19:44496639-44496684	chr19:44496461-44497794	Pitta
**A_17_P17145805**	0.17	-1.30	1.23	1.05	TEX101	chr19:48604260-48604304	chr19:48604007-48604475	Pitta
**A_17_P10969500**	0.18	-1.33	1.19	1.12	IRGQ	chr19:48788894-48788938	chr19:48788063-48789348	Pitta
**A_17_P17101924**	0.18	-1.40	1.09	1.28	SAFB2	chr19:5544872-5544916	chr19:5544709-5545194	Pitta
**A_17_P10867937**	0.16	-1.24	1.05	1.18	DUS3L	chr19:5736616-5736660	chr19:5736399-5736819	Pitta
**A_17_P10868863**	0.12	-1.29	1.08	1.19	RFX2	chr19:5958853-5958897	chr19:5958829-5959225	Pitta
**A_17_P17163205**	0.20	-1.20	-1.07	1.28	ZNF524	chr19:60805908-60805952	chr19:60805639-60806044	Pitta
**A_17_P17163242**	0.20	-1.36	1.01	1.35	ZNF784	chr19:60819883-60819927	chr19:60817161-60819979	Pitta
**A_17_P11006756**	0.19	-1.39	1.36	1.03	ZNF71	chr19:61825981-61826025	chr19:61824970-61825996	Pitta
**A_17_P10873676**	0.08	-1.47	1.21	1.21	TRAPPC 5	chr19:7653481-7653525	chr19:7651490-7653714	Pitta
**A_17_P10852207**	0.19	-1.34	1.15	1.16	C19orf6	chr19:962408-962452	chr19:958551-963222	Pitta
**A_17_P17093236**	0.09	-1.31	-1.04	1.37	GRIN3B	chr19:958681-958725	chr19:958551-963222	Pitta
**A_17_P01384691**	0.15	-1.23	-1.12	1.37	NCK2	chr2:105864115-105864159	#N/A	Pitta
**A_17_P15314342**	0.15	-1.37	1.09	1.26		chr2:118333450-118333494	chr2:118333045-118334633	Pitta
**A_17_P00940555**	0.19	-1.20	-1.02	1.22	TPO	chr2:1523421-1523465	chr2:1523279-1523496	Pitta
**A_17_P01868408**	0.17	-1.18	-1.11	1.31	ERBB4	chr2:213110924-213110968	chr2:213110425-213112112	Pitta
**A_17_P01990112**	0.12	-1.53	1.33	1.15	RBM44	chr2:238372531-238372579	chr2:238371747-238372760	Pitta
**A_17_P02002024**	0.18	-1.14	-1.15	1.31		chr2:240911465-240911511	#N/A	Pitta
**A_17_P15242925**	0.14	-1.19	-1.01	1.20	NRXN1	chr2:51108187-51108231	chr2:51108026-51109002	Pitta

Probe Name	p (Corr)	FC K vs P	FC V vs K	FC P vs V	Gene Symbol	Probe Coordinates	CpG Island	Prakriti More Methylated
**A_17_P00960791**	0.13	-1.29	-1.01	1.31	SOX11	chr2:5749737-5749795	chr2:5749492-5751659	Pitta
**A_17_P15250661**	0.17	-1.39	-1.09	1.51	SPRED2	chr2:65511939-65511983	chr2:65511272-65513490	Pitta
**A_17_P15290121**	0.11	-1.40	1.12	1.26	ZAP70	chr2:97706958-97707002	chr2:97706829-97707391	Pitta
**A_17_P11110627**	0.15	-1.25	1.05	1.18	NKX2-2	chr20:21444544-21444588	chr20:21433932-21444714	Pitta
**A_17_P11130647**	0.18	-1.44	1.03	1.39	HM13	chr20:29598662-29598714	#N/A	Pitta
**A_17_P11132298**	0.18	-1.30	1.20	1.09	XKR7	chr20:30048135-30048180	chr20:30047981-30048439	Pitta
**A_17_P11182714**	0.10	-1.55	1.31	1.19	JPH2	chr20:42222597-42222647	#N/A	Pitta
**A_17_P11207731**	0.07	-1.45	1.14	1.27	KCNG1	chr20:49059951-49059995	chr20:49059470-49060250	Pitta
**A_17_P11259427**	0.09	-1.53	1.15	1.33	GTPBP5	chr20:60214914-60214960	#N/A	Pitta
**A_17_P17212817**	0.12	-1.23	-1.15	1.41	NPBWR2	chr20:62207925-62207969	chr20:62207826-62208515	Pitta
**A_17_P11399818**	0.12	-1.30	-1.00	1.30	RIPK4	chr21:42034331-42034375	chr21:42033985-42035017	Pitta
**A_17_P11414026**	0.19	-1.43	1.11	1.29	ITGB2	chr21:45131072-45131117	chr21:45130936-45131223	Pitta
**A_17_P11417076**	0.11	-1.22	1.10	1.10	COL18A1	chr21:45720186-45720230	chr21:45719244-45720740	Pitta
**A_17_P11418747**	0.18	-1.44	1.33	1.08	PCBP3	chr21:46158437-46158481	chr21:46158375-46158877	Pitta
**A_17_P17244824**	0.13	-1.42	1.20	1.19	COL6A1	chr21:46248236-46248280	chr21:46246272-46248311	Pitta
**A_17_P11430476**	0.07	-1.39	1.13	1.23	GP1BB	chr22:18089382-18089426	chr22:18089081-18095311	Pitta
**A_17_P17255881**	0.20	-1.29	1.12	1.15	ARVCF	chr22:18340472-18340516	#N/A	Pitta
**A_17_P11441809**	0.16	-1.33	1.21	1.09	GNAZ	chr22:21768363-21768407	chr22:21767921-21768431	Pitta
**A_17_P11472834**	0.07	-1.49	1.23	1.21	SMTN	chr22:29830570-29830626	chr22:29830396-29831239	Pitta
**A_17_P11532029**	0.08	-1.47	-1.02	1.51	CELSR1	chr22:45238469-45238513	chr22:45238279-45238893	Pitta
**A_17_P11549494**	0.09	-1.64	1.15	1.43	PANX2	chr22:48959791-48959835	chr22:48957405-48960027	Pitta
**A_17_P11549581**	0.06	-1.24	-1.08	1.34	TRABD	chr22:48975018-48975062	chr22:48974834-48975168	Pitta
**A_17_P02066591**	0.12	-1.34	1.05	1.28	IQSEC1	chr3:12952631-12952675	chr3:12951966-12953182	Pitta
**A_17_P15496120**	0.15	-1.26	1.01	1.25	CHST2	chr3:144323433-144323477	chr3:144320572-144323525	Pitta
**A_17_P02813541**	0.08	-1.43	1.05	1.37		chr3:182927687-182927731	chr3:182927100-182927691	Pitta
**A_17_P15527670**	0.18	-1.28	1.00	1.27	NCBP2	chr3:198154058-198154102	chr3:198153089-198154448	Pitta
**A_17_P02238805**	0.18	-1.17	-1.08	1.26	PPM1M	chr3:52255066-52255110	chr3:52254689-52255180	Pitta
**A_17_P03335750**	0.19	-1.30	1.11	1.17	LEF1	chr4:109308016-109308060	chr4:109307196-109309856	Pitta
**A_17_P02888915**	0.17	-1.27	-1.01	1.28	CTBP1	chr4:1209559-1209603	chr4:1208943-1209718	Pitta
**A_17_P15532581**	0.13	-1.35	1.04	1.29	CTBP1	chr4:1231416-1231460	chr4:1231412-1234111	Pitta
**A_17_P15619322**	0.09	-1.39	-1.07	1.49	CCRN4L	chr4:140155994-140156038	chr4:140155893-140156878	Pitta
**A_17_P15646178**	0.10	-1.24	-1.18	1.46		chr4:186285516-186285560	chr4:186285535-186287214	Pitta
**A_17_P02897080**	0.14	-1.17	-1.10	1.28	RGS12	chr4:3345745-3345789	chr4:3345629-3345943	Pitta
**A_17_P03113335**	0.15	-1.13	-1.07	1.21	KIAA121 1	chr4:56876262-56876306	chr4:56875714-56876904	Pitta
**A_17_P02910611**	0.13	-1.39	1.21	1.15	TBC1D14	chr4:6976398-6976442	chr4:6976112-6976492	Pitta
**A_17_P03196043**	0.18	-1.38	1.20	1.15	SHROO M3	chr4:77881114-77881158	chr4:77880473-77882108	Pitta
**A_17_P15584199**	0.19	-1.24	1.03	1.21	CNOT6L	chr4:78959593-78959637	chr4:78958731-78960579	Pitta

Probe Name	p (Corr)	FC K vs P	FC V vs K	FC P vs V	Gene Symbol	Probe Coordinates	CpG Island	Prakriti More Methylated
**A_17_P03712024**	0.09	-1.21	-1.15	1.39	SLC12A7	chr5:1113821-1113865	#N/A	Pitta
**A_17_P15752776**	0.17	-1.37	1.05	1.31	TNIP1	chr5:150440629-150440673	chr5:150440279-150441057	Pitta
**A_17_P15755953**	0.14	-1.18	-1.03	1.22		chr5:157050279-157050323	chr5:157049727-157050957	Pitta
**A_17_P04450856**	0.16	-1.30	-1.01	1.31		chr5:167889718-167889762	chr5:167888797-167889949	Pitta
**A_17_P04483518**	0.13	-1.36	1.02	1.33	ARL10	chr5:175726163-175726207	#N/A	Pitta
**A_17_P04498479**	0.15	-1.30	1.10	1.18	FLT4	chr5:179981320-179981364	chr5:179978395-179981503	Pitta
**A_17_P03716237**	0.09	-1.27	1.07	1.18		chr5:2003748-2003792	chr5:2003624-2003922	Pitta
**A_17_P03709970**	0.16	-1.31	1.18	1.11	SLC9A3	chr5:541437-541481	chr5:541250-541710	Pitta
**A_17_P03710805**	0.12	-1.22	-1.12	1.36	TPPP	chr5:715737-715781	chr5:715283-716402	Pitta
**A_17_P15712789**	0.19	-1.18	-1.04	1.23	OTP	chr5:76970756-76970800	chr5:76970337-76971052	Pitta
**A_17_P05138648**	0.15	-1.31	1.11	1.17	STX11	chr6:144549992-144550036	chr6:144549519-144550390	Pitta
**A_17_P15869463**	0.10	-1.66	1.09	1.52	STX11	chr6:144550181-144550225	chr6:144549519-144550390	Pitta
**A_17_P05196806**	0.16	-1.24	-1.04	1.29		chr6:156993826-156993870	chr6:156993848-156994138	Pitta
**A_17_P05207112**	0.10	-1.28	-1.09	1.39	FNDC1	chr6:159574383-159574427	chr6:159573223-159575387	Pitta
**A_17_P05225506**	0.19	-1.39	1.26	1.10	PACRG	chr6:163490524-163490568	chr6:163490152-163490804	Pitta
**A_17_P05229467**	0.14	-1.30	1.08	1.21		chr6:164175647-164175692	chr6:164175440-164176052	Pitta
**A_17_P15893756**	0.14	-1.43	1.14	1.25		chr6:169166539-169166583	chr6:169166157-169167446	Pitta
**A_17_P15895099**	0.10	-1.53	1.27	1.21		chr6:170407093-170407142	#N/A	Pitta
**A_17_P15791645**	0.11	-1.21	1.01	1.21	HDGFL1	chr6:22678025-22678069	chr6:22677563-22678684	Pitta
**A_17_P04666282**	0.18	-1.39	1.07	1.29	PI16	chr6:37035262-37035314	#N/A	Pitta
**A_17_P04700837**	0.15	-1.28	1.21	1.06	NFKBIE	chr6:44351348-44351392	chr6:44351063-44352459	Pitta
**A_17_P04534649**	0.15	-1.35	-1.04	1.40	RREB1	chr6:7192294-7192341	chr6:7191664-7192571	Pitta
**A_17_P05653226**	0.18	-1.58	1.19	1.33	CUX1	chr7:101678400-101678448	chr7:101678385-101679354	Pitta
**A_17_P05262329**	0.09	-1.44	1.31	1.10		chr7:1337053-1337109	chr7:1336912-1337389	Pitta
**A_17_P05864221**	0.06	-1.40	1.05	1.33	ZNF425	chr7:148433011-148433055	#N/A	Pitta
**A_17_P05864622**	0.10	-1.36	1.12	1.21	ZNF282	chr7:148552292-148552336	chr7:148551954-148552770	Pitta
**A_17_P05864623**	0.20	-1.41	1.08	1.30	ZNF282	chr7:148552376-148552420	chr7:148551954-148552770	Pitta
**A_17_P05864921**	0.18	-1.30	1.11	1.18	ZNF783	chr7:148622813-148622857	chr7:148622588-148623114	Pitta
**A_17_P05263201**	0.18	-1.29	1.10	1.17	TMEM18 4A	chr7:1553963-1554007	chr7:1553766-1554219	Pitta
**A_17_P05410823**	0.18	-1.20	1.05	1.15	BMPER	chr7:34085275-34085319	chr7:34084988-34085284	Pitta
**A_17_P05480876**	0.16	-1.43	1.10	1.30		chr7:50169259-50169313	#N/A	Pitta
**A_17_P15905115**	0.12	-1.42	1.06	1.33	TNRC18	chr7:5367932-5367976	chr7:5367137-5368136	Pitta
**A_17_P05280895**	0.20	-1.21	-1.10	1.34	DAGLB	chr7:6453899-6453943	chr7:6453831-6454275	Pitta
**A_17_P15898198**	0.07	-1.49	1.23	1.22	C7orf20	chr7:902065-902109	chr7:901557-902174	Pitta
**A_17_P06533874**	0.15	-1.34	-1.00	1.35	FAM83H	chr8:144880329-144880373	chr8:144880209-144882966	Pitta
**A_17_P16148040**	0.08	-1.28	1.15	1.11	PARP10	chr8:145123956-145124000	chr8:145119281-145124293	Pitta
**A_17_P16054821**	0.11	-1.24	-1.01	1.25	DLGAP2	chr8:1484630-1484674	chr8:1484138-1485291	Pitta
**A_17_P06095117**	0.06	-1.59	1.28	1.25	SLC20A2	chr8:42413874-42413918	chr8:42413667-42414216	Pitta

Probe Name	p (Corr)	FC K vs P	FC V vs K	FC P vs V	Gene Symbol	Probe Coordinates	CpG Island	Prakriti More Methylated
**A_17_P16097951**	0.13	-1.31	-1.07	1.41	FAM110B	chr8:59221602-59221646	chr8:59220721-59221968	Pitta
**A_17_P16066643**	0.18	-1.55	1.47	1.05	CLDN23	chr8:8598063-8598107	chr8:8596541-8598277	Pitta
**A_17_P06946485**	0.10	-1.38	1.25	1.10	LHX6	chr9:124028917-124028961	chr9:124027564-124030907	Pitta
**A_17_P06993482**	0.18	-1.38	1.08	1.27	ABO	chr9:135121351-135121395	chr9:135120822-135121652	Pitta
**A_17_P07008897**	0.12	-1.21	-1.02	1.24	C9orf163	chr9:138497957-138498001	chr9:138496931-138498995	Pitta
**A_17_P07011231**	0.18	-1.24	-1.05	1.30	C9orf139	chr9:139032130-139032174	chr9:139031330-139032607	Pitta
**A_17_P07012367**	0.15	-1.14	-1.10	1.26	TOR4A	chr9:139293731-139293775	chr9:139292434-139295926	Pitta
**A_17_P06550494**	0.14	-1.26	1.05	1.21	KCNV2	chr9:2708646-2708690	chr9:2707903-2708953	Pitta
**A_17_P06694132**	0.19	-1.35	1.22	1.11	FAM219A	chr9:34390785-34390837	#N/A	Pitta
**A_17_P16175582**	0.18	-1.31	1.05	1.25	PAX5	chr9:37027000-37027044	chr9:37024135-37028341	Pitta
**A_17_P06832275**	0.18	-1.32	1.15	1.15		chr9:97586849-97586893	#N/A	Pitta
**A_17_P15000032**	0.11	-1.31	1.06	1.25	PPP2R3B	chrX:225449-225493	chrX:221585-228671	Pitta
**A_17_P17419852**	0.18	-1.29	-1.02	1.31	PPP2R3B	chrX:231389-231433	chrX:231035-232001	Pitta
**A_17_P17419854**	0.18	-1.35	1.04	1.31	PPP2R3B	chrX:231556-231600	chrX:231035-232001	Pitta
**A_17_P17419896**	0.11	-1.20	-1.10	1.32	PPP2R3B	chrX:238903-238947	chrX:238668-239872	Pitta
**A_17_P17419900**	0.07	-1.41	1.07	1.32	PPP2R3B	chrX:239206-239250	chrX:238668-239872	Pitta
**A_17_P15007885**	0.09	-1.30	-1.04	1.34	SDF4	chr1:1153635-1153679	chr1:1153365-1153758	Pitta
**A_17_P00002704**	0.13	-1.44	1.09	1.32	KIAA1751	chr1:1876477-1876524	chr1:1876228-1876730	Pitta
**A_17_P15169007**	0.12	-1.23	-1.09	1.35		chr1:206199428-206199472	chr1:206198950-206199740	Pitta
**A_17_P15176882**	0.15	-1.32	1.21	1.09		chr1:219135440-219135484	chr1:219135405-219135782	Pitta
**A_17_P00932638**	0.09	-1.35	1.09	1.24	OR5BU1	chr1:246921821-246921865	chr1:246921653-246922093	Pitta
**A_17_P00105407**	0.18	-1.23	1.05	1.17	AHDC1	chr1:27747150-27747194	chr1:27747004-27747225	Pitta
**A_17_P00011630**	0.16	-1.24	-1.11	1.38	TP73	chr1:3624691-3624735	chr1:3624493-3624961	Pitta
**A_17_P00022928**	0.16	-1.16	-1.07	1.24	RNF207	chr1:6191529-6191573	chr1:6191417-6192175	Pitta
**A_17_P07509808**	0.12	-1.29	-1.07	1.38	VAX1	chr10:118875778-118875822	chr10:118875555-118875874	Pitta
**A_17_P08126709**	0.14	-1.45	1.23	1.18	HNT	chr11:131066329-131066374	chr11:131066147-131066823	Pitta
**A_17_P16440976**	0.13	-1.30	1.06	1.23	PRDM11	chr11:45190895-45190939	chr11:45190798-45191004	Pitta
**A_17_P16472903**	0.10	-1.35	-1.02	1.38	P2RY2	chr11:72623864-72623910	chr11:72622927-72623876	Pitta
**A_17_P07596156**	0.19	-1.52	1.19	1.28	PNPLA2	chr11:814167-814211	chr11:813556-814878	Pitta
**A_17_P16675768**	0.15	-1.24	-1.18	1.47	RASA3	chr13:113785144-113785188	chr13:113784812-113785325	Pitta
**A_17_P16620214**	0.12	-1.29	-1.02	1.31	ATP8A2	chr13:25484386-25484430	chr13:25484287-25484761	Pitta
**A_17_P08800927**	0.18	-1.56	1.28	1.21		chr13:40007650-40007700	chr13:40007489-40007748	Pitta
**A_17_P09532891**	0.14	-1.22	-1.11	1.36	AKT1	chr14:104339605-104339649	chr14:104339530-104339808	Pitta
**A_17_P09765013**	0.18	-1.34	1.18	1.14	CSPG4	chr15:73767245-73767289	chr15:73766984-73767451	Pitta
**A_17_P09867028**	0.20	-1.26	1.12	1.13	IGF1R	chr15:97252312-97252356	chr15:97252088-97252436	Pitta
**A_17_P09886209**	0.07	-1.26	-1.00	1.27	CLCN7	chr16:1437880-1437924	chr16:1437451-1437938	Pitta
**A_17_P09892884**	0.07	-1.34	-1.05	1.42	ZNF213	chr16:3130994-3131038	chr16:3130766-3131390	Pitta
**A_17_P10101968**	0.04	-1.57	1.28	1.22	ZNF821	chr16:70451853-70451897	chr16:70451608-70451895	Pitta

Probe Name	p (Corr)	FC K vs P	FC V vs K	FC P vs V	Gene Symbol	Probe Coordinates	CpG Island	Prakriti More Methylated
**A_17_P10116687**	0.19	-1.48	1.10	1.35	LDHD	chr16:73706206-73706250	chr16:73705899-73706365	Pitta
**A_17_P10183796**	0.14	-1.35	1.22	1.11	CDK10	chr16:88288626-88288670	chr16:88288517-88288719	Pitta
**A_17_P10184743**	0.17	-1.21	1.13	1.07	MC1R	chr16:88513680-88513724	chr16:88513414-88513857	Pitta
**A_17_P16955656**	0.15	-1.26	1.03	1.22	B9D1	chr17:19188414-19188458	chr17:19188378-19188662	Pitta
**A_17_P10301478**	0.19	-1.22	1.23	-1.01	RASL10B	chr17:31086492-31086536	chr17:31086281-31086526	Pitta
**A_17_P16994403**	0.14	-1.18	-1.04	1.23		chr17:45334458-45334502	chr17:45334464-45334739	Pitta
**A_17_P10494722**	0.07	-1.22	-1.11	1.35	ASPSCR1	chr17:77560292-77560336	chr17:77560143-77560427	Pitta
**A_17_P10498050**	0.16	-1.32	1.03	1.28	TBCD	chr17:78366186-78366230	chr17:78366081-78366292	Pitta
**A_17_P17066943**	0.20	-1.30	-1.05	1.36		chr18:44756486-44756530	chr18:44755622-44756974	Pitta
**A_17_P17088496**	0.14	-1.24	-1.03	1.28	NFATC1	chr18:75272864-75272908	chr18:75272692-75273106	Pitta
**A_17_P10884206**	0.13	-1.40	1.24	1.13	LPPR2	chr19:11334118-11334162	chr19:11334094-11334354	Pitta
**A_17_P17094493**	0.11	-1.54	1.27	1.22	NDUFS7	chr19:1344235-1344279	chr19:1344155-1344470	Pitta
**A_17_P17094854**	0.16	-1.24	-1.06	1.32	REEP6	chr19:1447362-1447406	chr19:1447346-1447681	Pitta
**A_17_P10855796**	0.11	-1.27	1.13	1.13	C19orf34	chr19:1904943-1904987	chr19:1904778-1905023	Pitta
**A_17_P10855834**	0.20	-1.29	1.00	1.28	C19orf34	chr19:1912125-1912169	chr19:1912001-1912325	Pitta
**A_17_P10856369**	0.15	-1.18	-1.07	1.26	AP3D1	chr19:2071941-2071985	chr19:2071873-2072167	Pitta
**A_17_P10860755**	0.17	-1.27	1.00	1.27	C19orf28	chr19:3497112-3497156	chr19:3497144-3497382	Pitta
**A_17_P10861671**	0.09	-1.41	1.17	1.21	ZFR2	chr19:3773121-3773165	chr19:3772974-3773278	Pitta
**A_17_P17133363**	0.18	-1.48	1.20	1.24	LGI4	chr19:40309081-40309125	chr19:40309093-40309480	Pitta
**A_17_P10947254**	0.11	-1.54	1.11	1.38	MAG	chr19:40492729-40492773	chr19:40492558-40492871	Pitta
**A_17_P17099961**	0.13	-1.22	1.07	1.14	MAP2K2	chr19:4053366-4053410	chr19:4053299-4054041	Pitta
**A_17_P17100106**	0.07	-1.31	1.13	1.16	ANKRD24	chr19:4169026-4169070	chr19:4168518-4169068	Pitta
**A_17_P10975602**	0.15	-1.30	1.01	1.29	FBXO46	chr19:50928372-50928416	chr19:50928268-50928797	Pitta
**A_17_P17150377**	0.18	-1.29	1.09	1.19	GLTSCR1	chr19:52897218-52897262	chr19:52896341-52897588	Pitta
**A_17_P10997510**	0.09	-1.17	-1.13	1.31	BIRC8	chr19:58486423-58486467	chr19:58486223-58486544	Pitta
**A_17_P10871051**	0.13	-1.59	1.04	1.53	TRIP10	chr19:6697135-6697179	chr19:6697023-6697292	Pitta
**A_17_P10876473**	0.09	-1.28	1.13	1.13	ADAMTS10	chr19:8567234-8567278	chr19:8567080-8567287	Pitta
**A_17_P15309826**	0.12	-1.14	-1.17	1.33	PSD4	chr2:113673434-113673478	chr2:113672814-113673513	Pitta
**A_17_P01440558**	0.14	-1.30	-1.05	1.37		chr2:121215917-121215961	chr2:121215698-121216048	Pitta
**A_17_P01471357**	0.13	-1.29	1.24	1.04	UGCGL1	chr2:128565533-128565577	chr2:128565133-128565680	Pitta
**A_17_P15387418**	0.14	-1.27	1.18	1.07	AP1S3	chr2:224410872-224410916	chr2:224410206-224411034	Pitta
**A_17_P15227294**	0.14	-1.33	1.04	1.27		chr2:26249877-26249921	chr2:26249606-26250708	Pitta
**A_17_P01313303**	0.13	-1.56	1.25	1.25		chr2:81547830-81547874	chr2:81547833-81548192	Pitta
**A_17_P11199158**	0.05	-1.41	1.17	1.21	PREX1	chr20:46686588-46686632	chr20:46686341-46686625	Pitta
**A_17_P17203741**	0.08	-1.49	1.17	1.27	APCDD1L	chr20:56474833-56474877	chr20:56474855-56475072	Pitta
**A_17_P11261936**	0.15	-1.07	-1.17	1.26	NTSR1	chr20:60810690-60810734	chr20:60810303-60811665	Pitta
**A_17_P17209347**	0.10	-1.55	1.03	1.51	COL9A3	chr20:60942504-60942556	chr20:60942348-60942701	Pitta
**A_17_P11265853**	0.18	-1.44	1.25	1.14	KCNQ2	chr20:61555158-61555202	chr20:61554911-61555347	Pitta

Probe Name	p (Corr)	FC K vs P	FC V vs K	FC P vs V	Gene Symbol	Probe Coordinates	CpG Island	Prakriti More Methylated
**A_17_P11420951**	0.14	-1.38	1.20	1.14	PCNT	chr21:46673458-46673502	chr21:46673477-46673818	Pitta
**A_17_P17288601**	0.13	-1.34	1.10	1.22	SCUBE1	chr22:41953592-41953636	chr22:41953312-41953611	Pitta
**A_17_P11531748**	0.16	-1.40	1.08	1.29	CELSR1	chr22:45184339-45184386	chr22:45184277-45184670	Pitta
**A_17_P17297392**	0.11	-1.26	1.02	1.23	BRD1	chr22:48555592-48555636	chr22:48555616-48555868	Pitta
**A_17_P02880906**	0.16	-1.11	-1.15	1.28	PIGZ	chr3:198180526-198180570	chr3:198179713-198181459	Pitta
**A_17_P15434112**	0.10	-1.25	1.00	1.25	TESSP5	chr3:46767452-46767496	chr3:46766865-46767522	Pitta
**A_17_P02915134**	0.17	-1.29	1.00	1.29	AFAP1	chr4:7825426-7825470	chr4:7825395-7825669	Pitta
**A_17_P15730885**	0.15	-1.04	-1.22	1.27	STARD4	chr5:110875974-110876018	chr5:110875719-110876344	Pitta
**A_17_P15655103**	0.09	-1.47	1.04	1.41	SLC12A7	chr5:1138478-1138522	chr5:1138140-1138847	Pitta
**A_17_P04488388**	0.11	-1.26	1.01	1.25	B4GALT 7	chr5:176964086-176964130	chr5:176963771-176964190	Pitta
**A_17_P04496675**	0.18	-1.50	1.12	1.33	RASGEF 1C	chr5:179496008-179496052	chr5:179495805-179496385	Pitta
**A_17_P15893891**	0.18	-1.25	1.02	1.23		chr6:169281836-169281880	chr6:169281426-169281958	Pitta
**A_17_P05257546**	0.17	-1.25	1.08	1.16	DLL1	chr6:170434759-170434803	chr6:170434309-170435004	Pitta
**A_17_P15839073**	0.19	-1.15	-1.09	1.25	GJA10	chr6:90654067-90654111	chr6:90653928-90654374	Pitta
**A_17_P05647941**	0.20	-1.08	-1.12	1.21	POP7	chr7:100141117-100141161	chr7:100140950-100142015	Pitta
**A_17_P05884923**	0.09	-1.55	1.27	1.22	DPP6	chr7:154315500-154315544	chr7:154315335-154315563	Pitta
**A_17_P05899392**	0.19	-1.35	1.06	1.27	PTPRN2	chr7:157260939-157260983	chr7:157260924-157261165	Pitta
**A_17_P05277806**	0.16	-1.39	1.33	1.04	TNRC18	chr7:5380242-5380286	chr7:5380271-5380736	Pitta
**A_17_P06529446**	0.13	-1.34	1.05	1.28	BAI1	chr8:143606456-143606500	chr8:143606380-143606604	Pitta
**A_17_P06532403**	0.05	-1.40	-1.00	1.40	RHPN1	chr8:144534023-144534067	chr8:144533773-144534097	Pitta
**A_17_P06536119**	0.19	-1.42	1.07	1.33	VPS28	chr8:145620418-145620462	chr8:145620242-145620522	Pitta
**A_17_P16250088**	0.14	-1.26	-1.12	1.40	NIPSNAP 3B	chr9:106566459-106566503	chr9:106565993-106566600	Pitta
**A_17_P06996512**	0.12	-1.33	1.08	1.23	VAV2	chr9:135748521-135748565	chr9:135748282-135748723	Pitta
**A_17_P16277133**	0.16	-1.27	1.04	1.22	NOTCH1	chr9:138522512-138522556	chr9:138522509-138522722	Pitta
**A_17_P07012863**	0.18	-1.23	-1.05	1.29	ENTPD8	chr9:139449475-139449519	chr9:139449275-139449531	Pitta
**A_17_P11902586**	0.20	-1.20	-1.45	1.73	PLS3	chrX:114702099-114702158	chrX:114701670-114702484	Pitta
**A_17_P17459458**	0.09	-1.44	1.14	1.26		chrY:18180933-18180977	chrY:18180718-18180981	Pitta
**A_17_P15002717**	0.17	-1.31	1.04	1.27	DAZ2	chrY:25389269-25389313	chrY:25389277-25389504	Pitta
**A_17_P00000918**	0.15	-1.51	2.55	-1.69	C1orf159	chr1:1041667-1041711	chr1:1041041-1042308	Vata
**A_17_P00476257**	0.17	-1.04	1.36	-1.31	NHLH2	chr1:116182853-116182897	chr1:116181882-116183887	Vata
**A_17_P00520487**	0.16	1.05	1.20	-1.25	UBE2Q1	chr1:152798232-152798276	#N/A	Vata
**A_17_P15133805**	0.14	1.09	1.29	-1.41	ARHGEF 11	chr1:155281943-155281987	chr1:155281773-155282853	Vata
**A_17_P15133808**	0.18	1.07	1.28	-1.38	ARHGEF 11	chr1:155282388-155282432	chr1:155281773-155282853	Vata
**A_17_P15148753**	0.16	1.18	1.17	-1.37	KIAA161 4	chr1:179190346-179190390	#N/A	Vata
**A_17_P00836529**	0.20	-1.01	1.51	-1.50		chr1:224376687-224376731	chr1:224375582-224377099	Vata
**A_17_P15188278**	0.18	1.10	1.17	-1.29	WNT3A	chr1:226313367-226313411	chr1:226313254-226313820	Vata
**A_17_P15190822**	0.18	1.01	1.19	-1.20	COG2	chr1:228845169-228845213	chr1:228844700-228845216	Vata

Probe Name	p (Corr)	FC K vs P	FC V vs K	FC P vs V	Gene Symbol	Probe Coordinates	CpG Island	Prakriti More Methylated
**A_17_P00096243**	0.15	-1.06	1.51	-1.42	RUNX3	chr1:25130037-25130082	chr1:25128114-25131592	Vata
**A_17_P15062196**	0.10	1.17	1.29	-1.51		chr1:54726430-54726474	#N/A	Vata
**A_17_P00378169**	0.15	1.43	1.10	-1.57	GFI1	chr1:92720692-92720736	chr1:92718495-92725197	Vata
**A_17_P07424750**	0.17	-1.07	1.37	-1.29		chr10:101271673-101271726	chr10:101271171-101272106	Vata
**A_17_P07056470**	0.12	1.15	1.18	-1.35	GATA3	chr10:8137972-8138017	chr10:8131380-8138335	Vata
**A_17_P08066878**	0.13	1.07	1.23	-1.31	CBL	chr11:118582256-118582300	chr11:118581851-118582487	Vata
**A_17_P07598567**	0.14	1.09	1.23	-1.34	BRSK2	chr11:1387430-1387474	#N/A	Vata
**A_17_P07722805**	0.14	1.08	1.33	-1.44	KCNA4	chr11:29994525-29994570	#N/A	Vata
**A_17_P07796099**	0.08	1.28	1.14	-1.47	DGKZ	chr11:46310618-46310663	#N/A	Vata
**A_17_P07813513**	0.16	1.04	1.23	-1.28	RTN4RL 2	chr11:56988832-56988876	#N/A	Vata
**A_17_P07838279**	0.09	1.28	1.14	-1.45	BAD	chr11:63793258-63793302	#N/A	Vata
**A_17_P07844041**	0.09	1.16	1.21	-1.41	MAP3K1 1	chr11:65131933-65131977	#N/A	Vata
**A_17_P16585768**	0.12	1.24	1.20	-1.48	ARL1	chr12:100325553-100325597	chr12:100325178-100326284	Vata
**A_17_P08643060**	0.16	1.23	1.11	-1.36	FLJ20674	chr12:117025343-117025388	#N/A	Vata
**A_17_P16602848**	0.19	1.20	1.14	-1.37		chr12:127317263-127317307	chr12:127316994-127319104	Vata
**A_17_P09148066**	0.19	-1.05	1.36	-1.29	LAMP1	chr13:113024165-113024211	#N/A	Vata
**A_17_P16694876**	0.06	1.21	1.23	-1.48	PAX9	chr14:36196526-36196570	chr14:36196537-36198025	Vata
**A_17_P09369128**	0.19	-1.14	1.30	-1.15	GALNTL 1	chr14:68796561-68796605	chr14:68796003-68798146	Vata
**A_17_P09709091**	0.14	-1.15	1.89	-1.64	RPS27L	chr15:61236701-61236745	chr15:61236471-61236927	Vata
**A_17_P09853755**	0.16	1.14	1.27	-1.45	NR2F2	chr15:94677097-94677141	chr15:94674412-94678725	Vata
**A_17_P09929182**	0.19	1.12	1.27	-1.42		chr16:11797758-11797802	#N/A	Vata
**A_17_P09890597**	0.16	1.07	1.32	-1.42	NTN2L	chr16:2463409-2463453	chr16:2461087-2465930	Vata
**A_17_P16879782**	0.13	1.16	1.10	-1.28	FUS	chr16:31098515-31098559	chr16:31098321-31099726	Vata
**A_17_P16899937**	0.14	1.20	1.21	-1.46	CPNE2	chr16:55684599-55684643	chr16:55683545-55684611	Vata
**A_17_P10048393**	0.11	1.19	1.29	-1.53		chr16:58346589-58346633	#N/A	Vata
**A_17_P16904676**	0.16	1.33	1.18	-1.56	CDH11	chr16:63712294-63712338	chr16:63712157-63715165	Vata
**A_17_P16920502**	0.14	1.24	1.15	-1.43	USP10	chr16:83290852-83290896	chr16:83290664-83291529	Vata
**A_17_P10170217**	0.15	1.29	1.23	-1.59		chr16:85088191-85088235	#N/A	Vata
**A_17_P10178117**	0.19	-1.05	1.31	-1.26	ZFPM1	chr16:87049587-87049631	chr16:87046230-87050015	Vata
**A_17_P10305343**	0.14	1.24	1.20	-1.49	LHX1	chr17:32367938-32367982	chr17:32366012-32374988	Vata
**A_17_P10323687**	0.17	1.12	1.20	-1.35		chr17:37076012-37076057	#N/A	Vata
**A_17_P16938072**	0.17	1.15	1.15	-1.32	TM4SF5	chr17:4632710-4632754	chr17:4632443-4633062	Vata
**A_17_P10392473**	0.15	1.13	1.20	-1.35	OR4D1	chr17:53589241-53589286	#N/A	Vata
**A_17_P16940879**	0.09	1.07	1.21	-1.29	DNAH2	chr17:7584440-7584484	#N/A	Vata
**A_17_P10497112**	0.15	1.01	1.43	-1.45	FOXK2	chr17:78135453-78135512	#N/A	Vata
**A_17_P10497379**	0.11	1.29	1.09	-1.41	WDR45L	chr17:78198551-78198595	chr17:78198590-78199613	Vata
**A_17_P10222988**	0.13	1.28	1.22	-1.56	USP43	chr17:9489360-9489404	chr17:9489114-9490341	Vata
**A_17_P10223507**	0.18	1.09	1.20	-1.31	DHRS7C	chr17:9615521-9615565	#N/A	Vata

Probe Name	p (Corr)	FC K vs P	FC V vs K	FC P vs V	Gene Symbol	Probe Coordinates	CpG Island	Prakriti More Methylated
**A_17_P10888170**	0.18	1.09	1.25	-1.36	NFIX	chr19:12986307-12986360	#N/A	Vata
**A_17_P10855951**	0.07	1.03	1.23	-1.27	BTBD2	chr19:1944329-1944373	#N/A	Vata
**A_17_P17098918**	0.14	1.18	1.15	-1.36	GIPC3	chr19:3536846-3536890	chr19:3536357-3536947	Vata
**A_17_P17091498**	0.17	1.14	1.17	-1.33	SHC2	chr19:373110-373154	chr19:373147-373629	Vata
**A_17_P10865529**	0.16	1.18	1.12	-1.32	JMJD2B	chr19:5027923-5027967	#N/A	Vata
**A_17_P10975722**	0.12	1.05	1.31	-1.37	DMPK	chr19:50966235-50966279	chr19:50962539-50967007	Vata
**A_17_P10983703**	0.16	-1.08	1.26	-1.17	GRWD1	chr19:53647740-53647784	#N/A	Vata
**A_17_P17152528**	0.10	1.27	1.13	-1.43	SLC17A7	chr19:54629127-54629171	#N/A	Vata
**A_17_P10996520**	0.18	1.22	1.11	-1.35	ZNF320	chr19:58092228-58092272	#N/A	Vata
**A_17_P10851888**	0.16	1.26	1.19	-1.50	KISS1R	chr19:870293-870337	chr19:868034-871787	Vata
**A_17_P01402648**	0.09	-1.08	1.45	-1.34	BCL2L11	chr2:111595110-111595154	chr2:111591677-111597436	Vata
**A_17_P01433015**	0.14	1.18	1.17	-1.38		chr2:119485563-119485607	#N/A	Vata
**A_17_P15383296**	0.18	1.15	1.15	-1.33	ARPC2	chr2:218790220-218790264	chr2:218789898-218790277	Vata
**A_17_P01965004**	0.11	1.04	1.29	-1.35	NGEF	chr2:233474291-233474335	chr2:233474093-233474363	Vata
**A_17_P02007683**	0.20	1.16	1.17	-1.36		chr2:242129619-242129663	chr2:242129117-242130491	Vata
**A_17_P01246053**	0.14	1.14	1.18	-1.34	MEIS1	chr2:66662181-66662225	chr2:66662072-66662908	Vata
**A_17_P01279947**	0.09	1.12	1.41	-1.58	BOLA3	chr2:74228236-74228280	#N/A	Vata
**A_17_P17194412**	0.17	1.10	1.13	-1.24	KCNK15	chr20:42812144-42812188	chr20:42812082-42812852	Vata
**A_17_P11328864**	0.19	1.10	1.12	-1.24	ADAMTS 1	chr21:27139853-27139897	chr21:27138429-27139988	Vata
**A_17_P11436538**	0.16	1.20	1.23	-1.47	PPIL2	chr22:20341644-20341688	chr22:20341565-20342516	Vata
**A_17_P11458450**	0.14	-1.13	1.47	-1.30		chr22:26365187-26365231	chr22:26364987-26365310	Vata
**A_17_P15483610**	0.17	1.13	1.17	-1.33	OSBPL11	chr3:126797105-126797149	chr3:126796673-126797296	Vata
**A_17_P15608440**	0.12	1.07	1.20	-1.28	PDE5A	chr4:120769369-120769413	chr4:120767651-120769698	Vata
**A_17_P02890618**	0.19	1.17	1.13	-1.32	SLBP	chr4:1682969-1683013	#N/A	Vata
**A_17_P03045453**	0.08	1.16	1.26	-1.47	RELL1	chr4:37364557-37364601	chr4:37363796-37365008	Vata
**A_17_P15582787**	0.11	1.21	1.17	-1.42	DKFZP56 4O0823	chr4:76078358-76078402	chr4:76077324-76078955	Vata
**A_17_P03191580**	0.20	-1.12	1.27	-1.14	G3BP2	chr4:76816875-76816919	chr4:76816659-76817944	Vata
**A_17_P15729201**	0.18	1.11	1.44	-1.59	EFNA5	chr5:107035013-107035057	chr5:107033029-107036090	Vata
**A_17_P03716472**	0.12	-1.12	1.76	-1.57		chr5:2060871-2060915	chr5:2060315-2060879	Vata
**A_17_P04030065**	0.20	1.09	1.35	-1.48	ZBED3	chr5:76418965-76419024	#N/A	Vata
**A_17_P04109493**	0.18	1.04	1.19	-1.23	NR2F1	chr5:92942625-92942669	chr5:92940526-92943093	Vata
**A_17_P15863433**	0.16	1.12	1.22	-1.37	EYA4	chr6:133604893-133604937	chr6:133603779-133605279	Vata
**A_17_P05146839**	0.11	1.29	1.16	-1.51	SHPRH	chr6:146326796-146326840	chr6:146326488-146327394	Vata
**A_17_P05164207**	0.13	1.08	1.31	-1.41	ZC3H12D	chr6:149819859-149819909	chr6:149819485-149819882	Vata
**A_17_P15888696**	0.20	1.02	1.24	-1.27	RPS6KA2	chr6:166960688-166960732	chr6:166959952-166961669	Vata
**A_17_P04818976**	0.19	1.22	1.17	-1.43	EEF1A1	chr6:74290271-74290315	#N/A	Vata
**A_17_P15838736**	0.13	-1.13	1.74	-1.54	RRAGD	chr6:90178308-90178352	chr6:90177593-90179089	Vata
**A_17_P05647572**	0.13	1.13	1.19	-1.35	PCOLCE	chr7:100039906-100039950	chr7:100039550-100041588	Vata

Probe Name	p (Corr)	FC K vs P	FC V vs K	FC P vs V	Gene Symbol	Probe Coordinates	CpG Island	Prakriti More Methylated
**A_17_P16038423**	0.20	-1.02	1.28	-1.25	CENTG3	chr7:150414936-150414980	chr7:150414413-150415973	Vata
**A_17_P05264209**	0.16	-1.04	1.56	-1.50	MAD1L1	chr7:1851970-1852014	#N/A	Vata
**A_17_P05277983**	0.16	1.14	1.37	-1.56	TNRC18	chr7:5429732-5429777	chr7:5425029-5435782	Vata
**A_17_P16122483**	0.16	1.16	1.25	-1.44	RIMS2	chr8:104580687-104580731	chr8:104580046-104583089	Vata
**A_17_P16150180**	0.19	1.24	1.10	-1.37	KIFC2	chr8:145669090-145669134	chr8:145668687-145669592	Vata
**A_17_P16087694**	0.14	1.21	1.18	-1.43	DDHD2	chr8:38208922-38208966	chr8:38207892-38209113	Vata
**A_17_P06095618**	0.18	1.03	1.28	-1.32	SLC20A2	chr8:42516359-42516406	#N/A	Vata
**A_17_P16101526**	0.19	-1.07	1.34	-1.25	CYP7B1	chr8:65873654-65873698	chr8:65873544-65874276	Vata
**A_17_P06925033**	0.18	-1.04	1.39	-1.34	ASTN2	chr9:119215732-119215778	chr9:119215074-119217317	Vata
**A_17_P06965577**	0.14	1.19	1.21	-1.44	LMX1B	chr9:128412573-128412617	chr9:128412558-128417927	Vata
**A_17_P16267381**	0.15	-1.15	1.30	-1.13	USP20	chr9:131676727-131676771	chr9:131676607-131677042	Vata
**A_17_P16269977**	0.16	1.12	1.16	-1.30	BARHL1	chr9:134446985-134447029	chr9:134444985-134448407	Vata
**A_17_P07007311**	0.18	1.19	1.15	-1.36	C9orf69	chr9:138154350-138154394	#N/A	Vata
**A_17_P07014509**	0.18	-1.05	1.28	-1.22	CACNA1 B	chr9:139896936-139896980	chr9:139896952-139897228	Vata
**A_17_P06755157**	0.18	-1.04	1.35	-1.29	GNA14	chr9:79452440-79452484	chr9:79452266-79453521	Vata
**A_17_P06830103**	0.16	1.16	1.31	-1.52	FANCC	chr9:97120060-97120119	#N/A	Vata
**A_17_P11973292**	0.15	-1.05	1.33	-1.26	TMEM32	chrX:134883586-134883630	chrX:134883337-134883824	Vata
**A_17_P12022540**	0.18	1.20	1.07	-1.28		chrX:149280672-149280716	chrX:149280198-149284748	Vata
**A_17_P11635969**	0.18	1.16	1.16	-1.34	SMS	chrX:21868429-21868473	#N/A	Vata
**A_17_P11644240**	0.18	1.18	1.19	-1.40	APOO	chrX:23835445-23835501	#N/A	Vata
**A_17_P11713525**	0.11	1.06	1.23	-1.30		chrX:39890124-39890168	chrX:39889984-39892010	Vata
**A_17_P11741961**	0.15	1.29	1.16	-1.50	PCSK1N	chrX:48575703-48575747	chrX:48574533-48575771	Vata
**A_17_P15010312**	0.19	1.27	1.15	-1.47	GNB1	chr1:1811237-1811281	chr1:1811265-1813132	Vata
**A_17_P00079016**	0.17	1.03	1.34	-1.38		chr1:20542163-20542207	chr1:20542153-20542605	Vata
**A_17_P16309726**	0.12	1.14	1.20	-1.37	PARD3	chr10:35143712-35143756	chr10:35143130-35145249	Vata
**A_17_P08041135**	0.13	1.13	1.34	-1.51	USP28	chr11:113251783-113251827	chr11:113251026-113251903	Vata
**A_17_P08409909**	0.17	1.03	1.30	-1.33	WIF1	chr12:63801988-63802032	chr12:63801145-63802130	Vata
**A_17_P16583091**	0.09	1.15	1.22	-1.40	NTN4	chr12:94709136-94709180	chr12:94708004-94709330	Vata
**A_17_P10494646**	0.17	-1.21	1.62	-1.34	ASPSCR1	chr17:77545486-77545530	chr17:77545430-77545783	Vata
**A_17_P01899846**	0.16	1.20	1.15	-1.38	IHH	chr2:219631194-219631238	chr2:219630398-219631374	Vata
**A_17_P11157507**	0.19	1.16	1.15	-1.34		chr20:36708427-36708471	chr20:36708105-36708548	Vata
**A_17_P17259018**	0.15	1.15	1.21	-1.39	SCARF2	chr22:19121087-19121131	chr22:19120638-19122665	Vata
**A_17_P15439286**	0.12	-1.00	1.40	-1.40	GLT8D1	chr3:52715167-52715211	chr3:52714577-52715466	Vata
**A_17_P15439957**	0.18	1.07	1.15	-1.22	CACNA1 D	chr3:53502949-53502993	chr3:53502967-53505146	Vata
**A_17_P05180076**	0.18	1.13	1.16	-1.31	FBXO5	chr6:153345341-153345385	chr6:153345101-153346666	Vata
**A_17_P06511769**	0.16	-1.03	1.30	-1.26	COL22A1	chr8:139959531-139959575	chr8:139959063-139959629	Vata
**A_17_P17323405**	0.16	-1.12	1.30	-1.16	SCML2	chrX:18282590-18282634	chrX:18281818-18283069	Vata
**A_17_P15191185**	0.13	1.16	1.18	-1.37	TRIM67	chr1:229363607-229363651	chr1:229363182-229363968	Vata

Probe Name	p (Corr)	FC K vs P	FC V vs K	FC P vs V	Gene Symbol	Probe Coordinates	CpG Island	Prakriti More Methylated
**A_17_P15201854**	0.12	1.27	1.14	-1.45	AKT3	chr1:242079337-242079381	chr1:242079335-242079868	Vata
**A_17_P00022558**	0.15	-1.22	1.37	-1.12	CHD5	chr1:6111653-6111697	chr1:6111460-6111707	Vata
**A_17_P16456617**	0.19	1.24	1.15	-1.43	MS4A13	chr11:60039525-60039569	chr11:60039359-60039621	Vata
**A_17_P16407122**	0.10	-1.20	1.47	-1.22	RPLP2	chr11:799931-799975	chr11:799202-800509	Vata
**A_17_P16588950**	0.18	1.20	1.14	-1.36	CKAP4	chr12:105166389-105166433	chr12:105165010-105166504	Vata
**A_17_P16600578**	0.18	1.13	1.22	-1.37	NCOR2	chr12:123569412-123569456	chr12:123569170-123569435	Vata
**A_17_P16536936**	0.18	1.10	1.24	-1.36	STRAP	chr12:15926712-15926756	chr12:15926198-15927103	Vata
**A_17_P08266542**	0.15	1.21	1.22	-1.47	MRPS35	chr12:27755183-27755227	chr12:27754900-27755348	Vata
**A_17_P16555929**	0.19	1.06	1.22	-1.30	ARF3	chr12:47637333-47637377	chr12:47637106-47637654	Vata
**A_17_P16561981**	0.18	1.08	1.19	-1.29	DNAJC14	chr12:54510420-54510464	chr12:54510053-54510579	Vata
**A_17_P16584560**	0.18	1.38	1.06	-1.47	ANKS1B	chr12:97812635-97812679	chr12:97812353-97813505	Vata
**A_17_P16695793**	0.17	1.17	1.10	-1.28	CLEC14 A	chr14:37795067-37795111	chr14:37794005-37795288	Vata
**A_17_P16707602**	0.14	1.11	1.15	-1.27	PPM1A	chr14:59785909-59785953	chr14:59785118-59786512	Vata
**A_17_P16718560**	0.12	1.20	1.07	-1.29	C14orf4	chr14:76569729-76569773	chr14:76569457-76569777	Vata
**A_17_P09820204**	0.11	1.62	1.68	-2.73		chr15:87703760-87703804	chr15:87702918-87703789	Vata
**A_17_P17002498**	0.10	1.19	1.15	-1.37	C17orf64	chr17:55853506-55853550	chr17:55853493-55854114	Vata
**A_17_P16939971**	0.16	1.14	1.14	-1.30	SLC16A1 3	chr17:6880291-6880335	chr17:6879861-6880814	Vata
**A_17_P16940799**	0.18	1.01	1.23	-1.24	ATP1B2	chr17:7495453-7495497	chr17:7494864-7496063	Vata
**A_17_P17040620**	0.13	1.03	1.20	-1.23	SLMO1	chr18:12410847-12410891	chr18:12410135-12410863	Vata
**A_17_P10887678**	0.15	1.63	1.30	-2.12	DNASE2	chr19:12853345-12853389	chr19:12852690-12853508	Vata
**A_17_P17095719**	0.14	1.12	1.18	-1.33	KLF16	chr19:1808254-1808298	chr19:1807725-1808443	Vata
**A_17_P10858284**	0.15	1.03	1.25	-1.29	DIRAS1	chr19:2674338-2674382	chr19:2674168-2674390	Vata
**A_17_P10850641**	0.17	-1.19	1.30	-1.09	MADCA M1	chr19:449083-449127	chr19:448799-449135	Vata
**A_17_P01432349**	0.13	1.17	1.14	-1.33	EN1	chr2:119323218-119323262	chr2:119323039-119323296	Vata
**A_17_P15208304**	0.12	1.14	1.26	-1.44	SNTG2	chr2:936346-936390	chr2:935686-936774	Vata
**A_17_P01352412**	0.15	1.14	1.13	-1.30	TSGA10	chr2:99124068-99124112	chr2:99123787-99124652	Vata
**A_17_P01352586**	0.12	1.04	1.27	-1.33	MITD1	chr2:99163875-99163920	chr2:99163780-99164055	Vata
**A_17_P11028038**	0.17	1.15	1.14	-1.31	SMOX	chr20:4078248-4078292	chr20:4077482-4078412	Vata
**A_17_P11531364**	0.14	-1.02	1.33	-1.31	GTSE1	chr22:45106070-45106114	chr22:45106042-45106367	Vata
**A_17_P02075053**	0.18	1.17	1.15	-1.34	NR2C2	chr3:14964861-14964905	chr3:14964490-14964988	Vata
**A_17_P15533063**	0.14	1.07	1.15	-1.23		chr4:1392265-1392309	chr4:1392197-1392435	Vata
**A_17_P15584344**	0.08	1.26	1.12	-1.40	FRAS1	chr4:79198114-79198158	chr4:79197389-79198280	Vata
**A_17_P15530163**	0.15	1.23	1.11	-1.37	ZNF718	chr4:97528-97572	chr4:97146-97898	Vata
**A_17_P04385223**	0.18	1.28	1.16	-1.49	HAND1	chr5:153835959-153836003	chr5:153835383-153836321	Vata
**A_17_P04416265**	0.18	1.17	1.24	-1.45	GABRB2	chr5:160907816-160907862	chr5:160907270-160907962	Vata
**A_17_P04033238**	0.18	1.26	1.07	-1.35	TBCA	chr5:77108055-77108099	chr5:77107401-77108059	Vata
**A_17_P04561149**	0.20	1.02	1.25	-1.27	PHACTR 1	chr6:12857882-12857926	chr6:12857863-12858485	Vata
**A_17_P04628008**	0.18	1.05	1.17	-1.23	PRSS16	chr6:27336114-27336158	chr6:27336079-27336343	Vata

Probe Name	p (Corr)	FC K vs P	FC V vs K	FC P vs V	Gene Symbol	Probe Coordinates	CpG Island	Prakriti More Methylated
**A_17_P15799342**	0.13	1.17	1.18	-1.38	KIAA1949	chr6:30762556-30762600	chr6:30762371-30762913	Vata
**A_17_P15810223**	0.20	1.10	1.17	-1.28	TRERF1	chr6:42527631-42527677	chr6:42527488-42527721	Vata
**A_17_P16017219**	0.12	1.17	1.12	-1.31	IRF5	chr7:128365676-128365720	chr7:128365054-128365992	Vata
**A_17_P05779911**	0.18	1.10	1.20	-1.32	KIAA0265	chr7:129497455-129497510	chr7:129497328-129497729	Vata
**A_17_P16038488**	0.08	1.25	1.18	-1.48	CENTG3	chr7:150451141-150451185	chr7:150450852-150451697	Vata
**A_17_P05373122**	0.15	1.12	1.14	-1.27	HNRNPA2B1	chr7:26207162-26207206	chr7:26205943-26208587	Vata
**A_17_P15920322**	0.18	1.11	1.16	-1.28	EVX1	chr7:27241906-27241950	chr7:27241580-27242151	Vata
**A_17_P15922176**	0.17	1.16	1.07	-1.23		chr7:29691929-29691973	chr7:29690713-29691961	Vata
**A_17_P05276154**	0.15	1.30	1.16	-1.51	FOXK1	chr7:4731654-4731698	chr7:4731210-4731778	Vata
**A_17_P16264995**	0.18	-1.20	1.52	-1.27	FPGS	chr9:129605055-129605099	chr9:129604684-129605387	Vata
**A_17_P07009040**	0.13	-1.08	1.36	-1.27	NOTCH1	chr9:138524000-138524044	chr9:138523890-138524247	Vata
**A_17_P06702424**	0.16	-1.10	1.37	-1.24	GNE	chr9:36248436-36248480	chr9:36248170-36248886	Vata
**A_17_P16244144**	0.11	1.07	1.22	-1.31	ERCC6L2	chr9:97824117-97824161	chr9:97823037-97824185	Vata
**A_17_P12053580**	0.17	1.08	1.21	-1.31	NLGN4Y	chrY:15146002-15146046	chrY:15145684-15146385	Vata

'P(corr)' is the Benzamin Hotchberg FDR corrected P-value, ' FC' is the fold change difference
