# Supplementary material for: DNA methylation analysis of phenotype specific stratified Indian population
Source: J Transl Med. 2015 May 8;13:151. doi: 10.1186/s12967-015-0506-0 (PMC4438459; doi:10.1186/s12967-015-0506-0)
Supplement: Additional file 10: Table S3. — Enrichment analysis of 501 differential methylated prakriti specific regions (mPSR) for genomic context and histone occupancy. [file 12967_2015_506_MOESM10_ESM.pdf]

Table S3: Enrichment analysis of 501 differential methylated prakriti specific regions (mPSR) for genomic context and histone occupancy.

**Overall Distribution**				
**#501**	**No of Feature**	**Features in the array**	**Chi-square Test**	
			**P-value**	**Odds Ratio**
**Upstream Promoter**	21	5360	0.04	1.55
**Promoter**	75	49331	<0.0001	0.73
**5'UTR**	36	19790	0.06	0.69
**Gene Body**	301	99219	0.009	1.2
**3'UTR**	19	3967	0.005	1.01
**Downstream**	17	9083	0.23	0.62
**Non-Intergenic**	32	12649	0.97	0.97
**CpG Island Context**				
**Upstream Promoter**	17	4397	0.09	1.5
**Promoter**	67	40871	0.0005	0.63
**5'UTR**	34	18469	0.058	0.71
**Gene Body**	256	81611	0.012	1.21
**3'UTR**	16	3097	0.0054	2
**Downstream**	12	7402	0.111	0.62
**Non-Intergenic**	25	9979	0.89	0.97
**CpG shore context**				
**Upstream Promoter**	4	963	0.21	1.5
**Promoter**	8	8460	0.01	0.63
**5'UTR**	2	1321	0.59	0.71
**Gene Body**	45	17608	0.43	1.21
**3'UTR**	3	870	0.44	2
**Downstream**	5	1681	0.03	0.62
**Non-Intergenic**	7	2670	0.66	0.97
