# Supplementary material for: DNA methylation analysis of phenotype specific stratified Indian population
Source: J Transl Med. 2015 May 8;13:151. doi: 10.1186/s12967-015-0506-0 (PMC4438459; doi:10.1186/s12967-015-0506-0)
Supplement: Additional file 11: Table S4. — Overall Enrichment for mPSRs in ENCODE regions. [file 12967_2015_506_MOESM11_ESM.pdf]

Table S4: Overall Enrichment for mPSRs in ENCODE regions

Regions	# Features of mPSRs	# Features of Agilent 244K	Chi-Square Test		
			P-value	Odds Ratio	FDR(Corrected p-value)
**H3K9ac**	274	154700	<0.0001	0.75	0.0002
**H3K27ac**	203	129540	<0.0001	0.75	0.0002
**H3H4me1**	344	168840	0.0031	0.85	0.0062
**H3K4me2**	320	174521	<0.0001	0.78	0.0002
**H3K4me3**	286	166666	<0.0001	0.73	0.0002
**H3K9me1**	361	144508	0.9611	0.96	0.9961
**H3K20me1**	467	184525	0.936	0.99	0.9961
**H3K27me3**	371	152724	0.6472	0.95	0.3208
**H3K36me3**	340	135609	0.9961	1.03	0.9961
**Polycomb repression**	180	64857	0.2717	1.03	0.4075
**CTCF**	139	72387	0.0057	0.78	0.0097
**DNAseI**	124	85540	<0.0001	0.63	0.0002
