# Supplementary material for: DNA methylation analysis of phenotype specific stratified Indian population
Source: J Transl Med. 2015 May 8;13:151. doi: 10.1186/s12967-015-0506-0 (PMC4438459; doi:10.1186/s12967-015-0506-0)
Supplement: Additional file 15: Figure S9. — Overview of methylation status of LHX1 in prakriti. A: Genomic position of amplicon, GC ratio and CpG density is shown at the top of the graphs. Green bar represents the CpG Island and arrow indicates the position of transcription start site. CpG density across a window size of 20bp is drawn. The red dotted box represents the amplicon for which bisulfite specific sequencing is performed. B: Average absolute methylation at individual CpG sites in three prakriti. The significant difference in methylation across CpG site is evaluated by two-way ANOVA and aster sign was represented for the significant (p≤0.05) CpG sites. C: Absolute methylation in individual CpG sites across three prakriti. A methylation cut off >10% was considered to call methylated sites and are represented in black circles. The circles in white were considered as unmethylated with respect to the cut-off given. The significant differentially methylated region is shown in red bordered circles. D: Quantitation of methylated CpG sites in the differentially methylated region with the same cut-off, highlighting the CpG sites with higher frequency of methylation in Vata prakriti. Cumulative methylation analysis for region of interest by averaging all CpG sites in the prakriti population showed higher level of methylation in Vata as compared to other prakriti. [file 12967_2015_506_MOESM15_ESM.pdf]

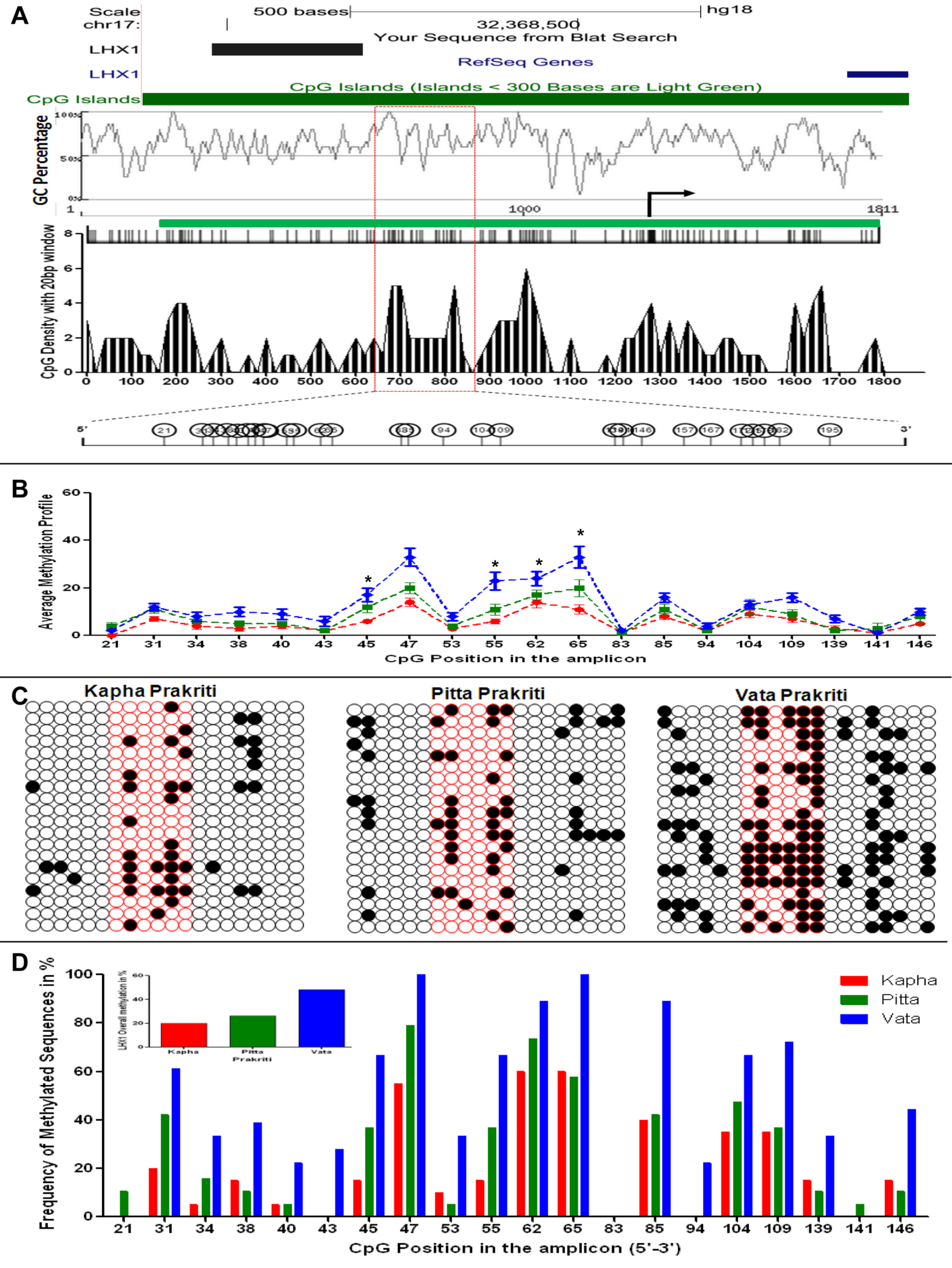

A
Scale
500 bases
hg18
chr17:
32,368,500
Your Sequence from Blat Search
LHX1
RefSeq Genes
LHX1
CpG Islands (Islands < 300 Bases are Light Green)
CpG Islands
GC Percentage
1000
1811
CpG Density with 20bp window
5'
3'
B
Average Methylation Profile
CpG Position in the amplicon
C
Kapha Prakriti
Pitta Prakriti
Vata Prakriti
D
Frequency of Methylated Sequences in %
Kapha
Pitta
Vata
Prakriti
CpG Position in the amplicon (5'-3')
