# Supplementary material for: DNA methylation analysis of phenotype specific stratified Indian population
Source: J Transl Med. 2015 May 8;13:151. doi: 10.1186/s12967-015-0506-0 (PMC4438459; doi:10.1186/s12967-015-0506-0)
Supplement: Additional file 14: Table S5. — Gene ontology and pathway analysis of prakriti methylated genes. [file 12967_2015_506_MOESM14_ESM.pdf]

Table S5: Gene ontology and pathway analysis of prakriti methylated genes.

Prakriti/Cluster/Category and Term	Count	%	P-Value	Fold Enrichment
**Kapha Prakriti**				
**Annotation Cluster 1:Enrichment Score: 1.83; Biological Process**				
GO:0046907~intracellular transport	5	25.00	9.78E-03	5.41
GO:0006913~nucleocytoplasmic transport	3	15.00	1.79E-02	13.69
GO:0051169~nuclear transport	3	15.00	1.83E-02	13.51
**Annotation Cluster 2:Enrichment Score: 1.65; Biological Process**				
GO:0006605~protein targeting	4	20.00	2.71E-03	13.25
GO:0046907~intracellular transport	5	25.00	9.78E-03	5.42
GO:0006886~intracellular protein transport	4	20.00	1.26E-02	7.61
GO:0034613~cellular protein localization	4	20.00	1.62E-02	6.93
GO:0070727~cellular macromolecule localization	4	20.00	1.65E-02	6.88
GO:0015031~protein transport	4	20.00	7.73E-02	3.74
GO:0045184~establishment of protein localization	4	20.00	7.90E-02	3.70
GO:0008104~protein localization	4	20.00	1.09E-01	3.23
**Annotation Cluster 3:Enrichment Score: 1.07; Cellular Component**				
GO:0044427~chromosomal part	3	15.00	5.70E-02	7.10
GO:0005694~chromosome	3	15.00	7.76E-02	5.95
GO:0043228~non-membrane-bounded organelle	6	30.00	1.04E-01	2.11
GO:0043232~intracellular non-membrane-bounded organelle	6	30.00	1.04E-01	2.11
**Annotation Cluster 4: Enrichment Score: 0.87; Cellular Component**				
GO:0005578~proteinaceous extracellular matrix	3	15.00	4.06E-02	8.56
GO:0031012~extracellular matrix	3	15.00	4.66E-02	7.94
GO:0044421~extracellular region part	3	15.00	2.55E-01	2.85
GO:0005576~extracellular region	3	15.00	6.30E-01	1.36
Annotation Cluster 5: Enrichment Score: 0.84; Cellular Component				
GO:0016337~cell-cell adhesion	3	15.00	5.12E-02	7.74
GO:0007155~cell adhesion	3	15.00	2.38E-01	3.05
GO:0022610~biological adhesion	3	15.00	2.39E-01	3.05
**Pitta Prakriti**				
**Annotation Cluster 1: Enrichment Score: 3.09; Cellular Component**				
GO:0044459~plasma membrane part	49	21.49	4.46E-06	1.91
GO:0031226~intrinsic to plasma membrane	25	10.96	6.47E-03	1.77

Prakriti/Cluster/Category and Term	Count	%	P-Value	Fold Enrichment
GO:0005887~integral to plasma membrane	23	10.09	1.83E-02	1.66
**Annotation Cluster 2: Enrichment Score: 2.44; Biological Process**				
GO:0030182~neuron differentiation	19	8.33	9.28E-06	3.45
GO:0048666~neuron development	15	6.58	9.07E-05	3.52
GO:0000904~cell morphogenesis involved in differentiation	11	4.82	9.80E-04	3.59
GO:0000902~cell morphogenesis	13	5.70	1.72E-03	2.91
GO:0030030~cell projection organization	13	5.70	2.27E-03	2.81
GO:0032989~cellular component morphogenesis	13	5.70	4.19E-03	2.61
GO:0007409~axonogenesis	8	3.51	1.07E-02	3.30
GO:0048858~cell projection morphogenesis	9	3.95	1.19E-02	2.92
GO:0032990~cell part morphogenesis	9	3.95	1.52E-02	2.80
GO:0031175~neuron projection development	9	3.95	1.52E-02	2.80
GO:0048667~cell morphogenesis involved in neuron differentiation	8	3.51	1.60E-02	3.05
GO:0048812~neuron projection morphogenesis	8	3.51	1.76E-02	2.99
GO:0007411~axon guidance	4	1.75	1.50E-01	2.97
**Annotation Cluster 3: Enrichment Score: 2.15; Biological Process**				
GO:0021537~telencephalon development	6	2.63	1.29E-03	7.35
GO:0030900~forebrain development	8	3.51	2.98E-03	4.19
GO:0021761~limbic system development	4	1.75	7.28E-03	9.95
GO:0021543~pallium development	4	1.75	1.96E-02	6.92
GO:0021766~hippocampus development	3	1.32	3.04E-02	10.85
**Annotation Cluster 4: Enrichment Score: 1.78; Molecular Function**				
GO:0046872~metal ion binding	70	30.70	4.38E-03	1.32
GO:0043167~ion binding	71	31.14	5.21E-03	1.31
GO:0043169~cation binding	70	30.70	5.61E-03	1.31
GO:0008270~zinc ion binding	38	16.67	7.50E-02	1.29
GO:0046914~transition metal ion binding	43	18.86	1.23E-01	1.21
**Annotation Cluster 5: Enrichment Score: 1.57; Biological process**				
GO:0045665~negative regulation of neuron differentiation	4	1.75	7.94E-03	9.65
GO:0051960~regulation of nervous system development	8	3.51	1.04E-02	3.32
GO:0060284~regulation of cell development	8	3.51	1.45E-02	3.11
GO:0050767~regulation of neurogenesis	7	3.07	1.79E-02	3.36
GO:0045596~negative regulation of cell differentiation	7	3.07	5.40E-02	2.58
GO:0045664~regulation of neuron differentiation	5	2.19	8.53E-02	2.99

Prakriti/Cluster/Category and Term	Count	%	P-Value	Fold Enrichment
GO:0045165~cell fate commitment	5	2.19	9.65E-02	2.86
**Annotation Cluster 6: Enrichment Score: 1.38; KEGG_Pathway**				
hsa04660:T cell receptor signaling pathway	7	3.07	1.20E-03	5.68
hsa04662:B cell receptor signaling pathway	5	2.19	9.56E-03	5.84
hsa04650:Natural killer cell mediated cytotoxicity	5	2.19	6.09E-02	3.30
hsa04722:Neurotrophin signaling pathway	4	1.75	1.61E-01	2.83
hsa04370:VEGF signaling pathway	3	1.32	2.05E-01	3.51
hsa04664:Fc epsilon RI signaling pathway	3	1.32	2.17E-01	3.37
Annotation Cluster 7:Enrichment Score: 1.37; Molecular function				
GO:0003700~transcription factor activity	24	10.53	2.88E-03	1.93
GO:0030528~transcription regulator activity	29	12.72	2.58E-02	1.50
GO:0003677~DNA binding	40	17.54	3.83E-02	1.34
GO:0043565~sequence-specific DNA binding	14	6.14	4.57E-02	1.80
GO:0006350~transcription	35	15.35	6.41E-02	1.33
GO:0006355~regulation of transcription, DNA-dependent	30	13.16	7.63E-02	1.35
GO:0051252~regulation of RNA metabolic process	30	13.16	9.47E-02	1.32
GO:0045449~regulation of transcription	39	17.11	1.62E-01	1.19
**Vata Prakriti**				
**Annotation Cluster 1:Enrichment Score: 2.57; Molecular Function**				
GO:0043565~sequence-specific DNA binding	17	11.64	4.45E-05	3.31
GO:0003700~transcription factor activity	21	14.38	1.57E-04	2.54
GO:0003677~DNA binding	35	23.97	5.37E-04	1.77
GO:0006355~regulation of transcription, DNA-dependent	27	18.49	3.41E-03	1.78
GO:0051252~regulation of RNA metabolic process	27	18.49	4.61E-03	1.74
GO:0030528~transcription regulator activity	23	15.75	6.77E-03	1.80
GO:0006357~regulation of transcription from RNA polymerase II promoter	13	8.90	2.06E-02	2.09
GO:0045449~regulation of transcription	32	21.92	2.65E-02	1.43
GO:0006350~transcription	27	18.49	2.83E-02	1.50
**Annotation Cluster 2:Enrichment Score: 1.44; Biological Process**				
GO:0031571~G1 DNA damage checkpoint	3	2.05	3.75E-03	31.81
GO:0007093~mitotic cell cycle checkpoint	4	2.74	5.77E-03	10.85
GO:0031575~G1/S transition checkpoint	3	2.05	8.96E-03	20.58
GO:0007346~regulation of mitotic cell cycle	5	3.42	4.06E-02	3.84
GO:0000075~cell cycle checkpoint	4	2.74	4.24E-02	5.13
GO:0000077~DNA damage checkpoint	3	2.05	6.27E-02	7.29
GO:0031570~DNA integrity checkpoint	3	2.05	7.22E-02	6.73

Prakriti/Cluster/Category and Term	Count	%	P-Value	Fold Enrichment
GO:0006974~response to DNA damage stimulus	7	4.79	9.83E-02	2.19
GO:0042770~DNA damage response, signal transduction	3	2.05	1.48E-01	4.37
GO:0051726~regulation of cell cycle	6	4.11	1.51E-01	2.11
**Annotation Cluster 3:Enrichment Score 1.40 Cellular component and KEGG Pathway**				
GO:0005891~voltage-gated calcium channel complex	3	2.05	1.19E-02	17.79
GO:0034704~calcium channel complex	3	2.05	1.77E-02	14.49
GO:0034702~ion channel complex	5	3.42	7.05E-02	3.18
hsa04010:MAPK signaling pathway	6	4.11	7.87E-02	2.54
GO:0034703~cation channel complex	4	2.74	7.90E-02	3.95
**Annotation Cluster 4:Enrichment Score: 1.37; Cellular component**				
GO:0030182~neuron differentiation	13	8.90	3.45E-04	3.46
GO:0048666~neuron development	9	6.16	8.32E-03	3.10
GO:0046658~anchored to plasma membrane	3	2.05	1.19E-02	17.79
GO:0007409~axonogenesis	6	4.11	2.44E-02	3.63
GO:0000902~cell morphogenesis	8	5.48	3.22E-02	2.62
GO:0048667~cell morphogenesis involved in neuron differentiation	6	4.11	3.29E-02	3.35
GO:0048812~neuron projection morphogenesis	6	4.11	3.52E-02	3.29
GO:0051301~cell division	7	4.79	4.00E-02	2.77
GO:0032989~cellular component morphogenesis	8	5.48	5.26E-02	2.35
GO:0000904~cell morphogenesis involved in differentiation	6	4.11	5.71E-02	2.87
GO:0048858~cell projection morphogenesis	6	4.11	5.79E-02	2.86
GO:0007163~establishment or maintenance of cell polarity	3	2.05	6.51E-02	7.14
GO:0032990~cell part morphogenesis	6	4.11	6.73E-02	2.73
GO:0031175~neuron projection development	6	4.11	6.73E-02	2.73
GO:0048729~tissue morphogenesis	5	3.42	6.74E-02	3.24
GO:0030030~cell projection organization	7	4.79	9.37E-02	2.22
GO:0031225~anchored to membrane	3	2.05	5.00E-01	1.78
GO:0031226~intrinsic to plasma membrane	8	5.48	8.27E-01	0.86
**Annotation Cluster 5:Enrichment Score: 1.35; Biological Process**				
GO:0006355~regulation of transcription, DNA-dependent	27	18.49	3.41E-03	1.78
GO:0051252~regulation of RNA metabolic process	27	18.49	4.61E-03	1.74
GO:0032583~regulation of gene-specific transcription	6	4.11	5.69E-03	5.22
GO:0045893~positive regulation of transcription, DNA-dependent	11	7.53	7.41E-03	2.69
GO:0051254~positive regulation of RNA metabolic process	11	7.53	7.84E-03	2.67

Prakriti/Cluster/Category and Term	Count	%	P-Value	Fold Enrichment
GO:0006357~regulation of transcription from RNA polymerase II promoter	13	8.90	2.06E-02	2.09
GO:0045941~positive regulation of transcription	11	7.53	2.18E-02	2.27
GO:0035239~tube morphogenesis	5	3.42	2.30E-02	4.59
GO:0010628~positive regulation of gene expression	11	7.53	2.61E-02	2.21
GO:0035295~tube development	6	4.11	3.96E-02	3.18
GO:0045935~positive regulation of nucleobase, nucleoside, nucleotide and nucleic acid metabolic process	11	7.53	3.97E-02	2.06
GO:0051173~positive regulation of nitrogen compound metabolic process	11	7.53	4.74E-02	1.99
GO:0010557~positive regulation of macromolecule biosynthetic process	11	7.53	5.16E-02	1.96
GO:0031328~positive regulation of cellular biosynthetic process	11	7.53	6.63E-02	1.87
GO:0009891~positive regulation of biosynthetic process	11	7.53	7.15E-02	1.85
GO:0010604~positive regulation of macromolecule metabolic process	12	8.22	1.12E-01	1.63
GO:0043193~positive regulation of gene-specific transcription	3	2.05	1.69E-01	4.02
GO:0045944~positive regulation of transcription from RNA polymerase II promoter	6	4.11	2.08E-01	1.89
